# Origin of Substituent-Modulated Regioselectivity in Phosphine-Catalyzed [3 + 2] Cyclization of Allenoates and Enones: A Kinetic Shift toward Curtin–Hammett Control

**DOI:** 10.1021/acs.joc.5c01466

**Published:** 2025-10-08

**Authors:** Gou-Tao Huang, Jen-Shiang K. Yu

**Affiliations:** † Department of Biological Science and Technology, 34914 National Yang Ming Chiao Tung University, Hsinchu City 300, Taiwan; ‡ Institute of Bioinformatics and Systems Biology, 34914 National Yang Ming Chiao Tung University, Hsinchu City 300, Taiwan; § Center for Intelligent Drug Systems and Smart Bio-Devices (IDS2B), 34914 National Yang Ming Chiao Tung University, Hsinchu City 300, Taiwan

## Abstract

The phosphine-catalyzed [3 + 2] cycloaddition of allenoates with enones provides an efficient route to five-membered carbocycles and exhibits regioselectivity that depends on the substituents of the substrates. To elucidate the origin of the substituent effects, density functional theory calculations and kinetic modeling are performed on the reactions of unsubstituted/substituted allenoates (**2**/**8**) with arylideneoxindoles (**e-iii**). Nucleophilic attack of PPh_3_ on the allenoate generates interconvertible *Z*-, *E*-, and twisted adducts: the former two participate in regioselective [3 + 2] cyclization. For **2**, the major γ-regioisomeric product forms via the *E*-adduct. Kinetic modeling predicts an α:γ ratio of 1:99, consistent with the experimentally observed 10:90 selectivity. By contrast, the reaction of **8** yields the α-regioisomer via the *Z*-adduct. The computed isomer ratio of 99:1 agrees with the experimental value of >95:5. The switch in regioselectivity is attributed to the interplay between electronic and steric effects. Secondary orbital interactions favor the γ-[3 + 2] pathway. Substituent-induced steric hindrance is found to elevate the activation barriers to cyclization, thereby shifting the kinetic regime toward Curtin–Hammett control and modulating regioselectivity. These findings highlight the pivotal role of adduct dynamics in phosphine catalysis and clarify the conditions under which Curtin–Hammett control governs product selectivity.

## Graphical abstract

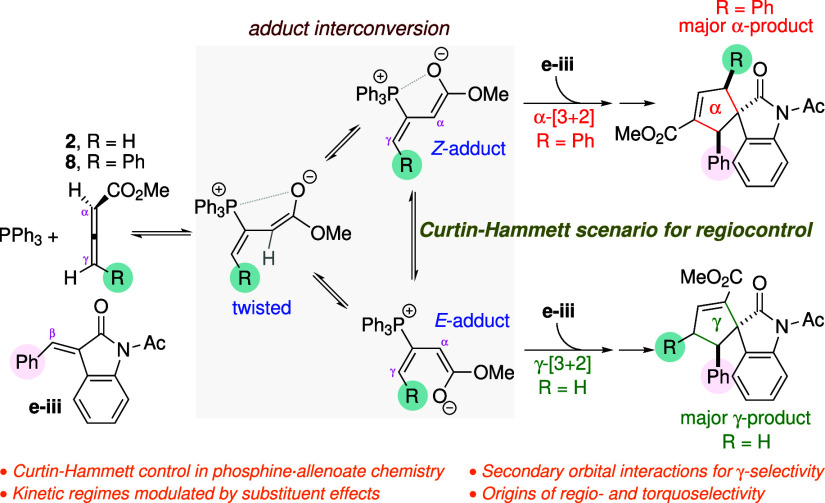

## 1 Introduction

Phosphine organocatalysis has emerged as a powerful strategy in modern synthetic chemistry, offering unique reactivity modes that enable the construction of complex molecular architectures under mild conditions.^1,2^ Owing to their nucleophilic nature, phosphine catalysts react with electron-deficient substrates such as allenoates and electron-poor alkenes to form reactive zwitterionic adducts.^3–7^ These intermediates undergo diverse annulation reactions, facilitating the efficient and selective formation of carbo- and heterocyclic ring systems.^1^ Phosphine-catalyzed [3 + 2] and [4 + 2] cycloaddition reactions have proven particularly valuable in the preparation of five- and six-membered ring skeletons, as well as bicyclic compounds.^1,8–13^ Lu’s [3 + 2] cyclization offers an efficient route to cyclopentene derivatives via the coupling of allenoates with electron-deficient alkenes (Scheme 1a).^14–16^ The methodology has also been successfully extended to the synthesis of spirocyclic scaffolds when cyclic enones bearing exocyclic double bonds are employed as substrates (Scheme 1b).^17–20^

**Scheme 1:**
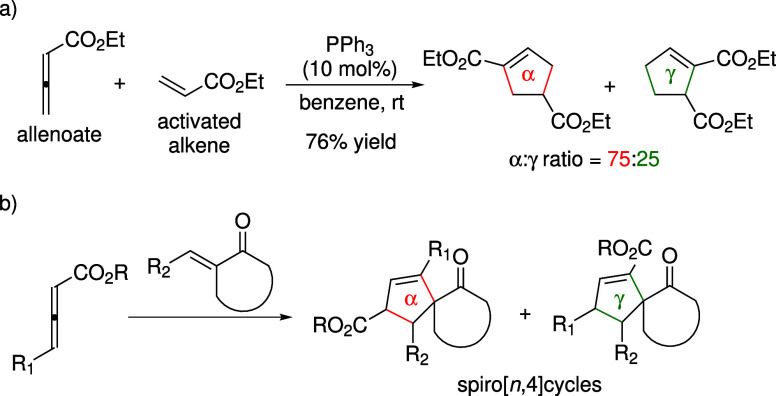
(a) Lu’s [3 + 2] Annulation, and (b) Cyclization between Allenoates and Cyclic Enones

Lu’s [3 + 2] annulation generally produces mixtures of α- and γ-regioisomers, and the regioselectivity is markedly modulated by the substituents on the allenoate/enone substrates (Schemes 2 and 3). For example, introducing an aryl substituent at the β-position of the exocyclic enone favors formation of the γ-product. When 2-methylene-2,3-dihydro-1*H*-inden-1-one (an unsubstituted enone) was used as the substrate, the α-regioisomer was obtained as the major product (Scheme 2a), with an α:γ ratio of 84:16.^21^ Bromine substitution on the arene ring slightly diminished selectivity, yielding a ratio of 78:22. The regioselectivity is similar to that reported in Lu’s original study shown in Scheme 1a. The Fu group later observed preferential generation of the γ-regioisomer when employing β-substituted enones such as chalcones in phosphepine-catalyzed asymmetric [3 + 2] cycloaddition.^22^ In particular, the utilization of enone **e-i** gave a single γ-regioisomer in 97% yield (Scheme 2b). Similar γ-selectivity was achieved using a chiral ferrocenophane-based catalyst developed by the Marinetti group (Scheme 2c).^23^ Furthermore, the effect of the β-substituent was also evident in the [3 + 2] cyclization between an allenyl methyl ketone and 2-methylene-3,4-dihydro-2*H*-naphthalen-1-one (**e-ii**, Scheme 2d), where the α:γ ratio reversed from 70:30 to 2:98 upon incorporation of a β-substituent on the enone.^24^ In the absence of the β-substituent, exocyclic enones preferentially afforded the α-product, with α:γ ratios ranging from 54:46 to 70:30.^24^ These observations demonstrate that the presence of the β-substituent enhances regioselectivity toward the γ-product.^25–32^

**Scheme 2:**
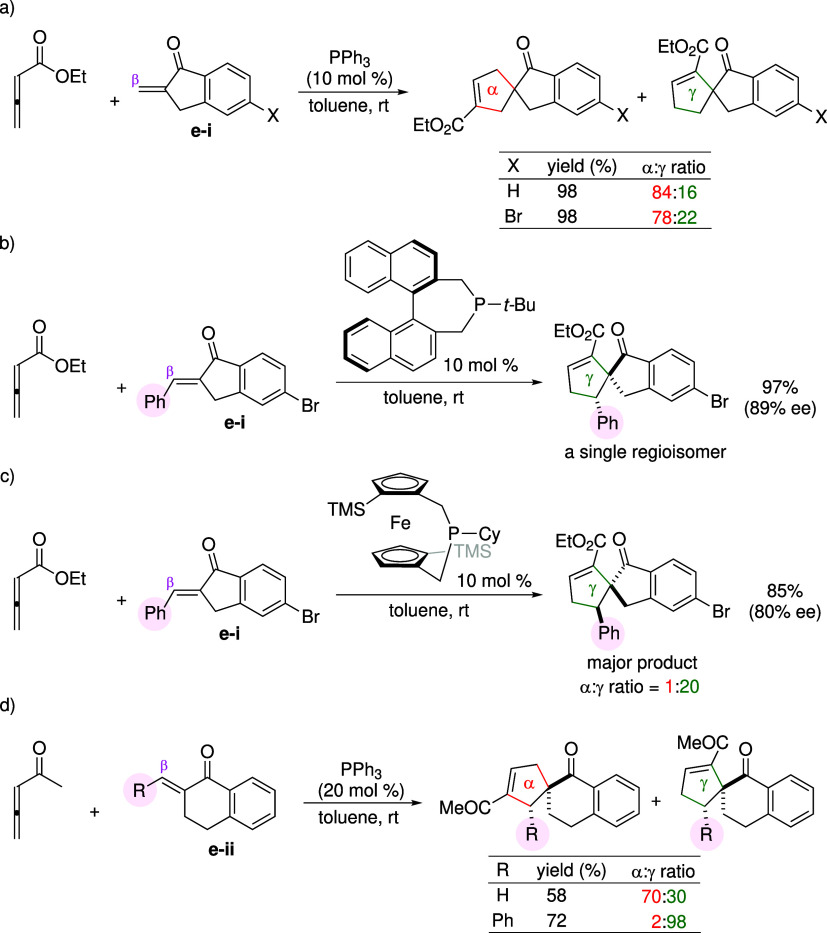
β-Substituent Effects of Enones on Regioselectivity

**Scheme 3:**
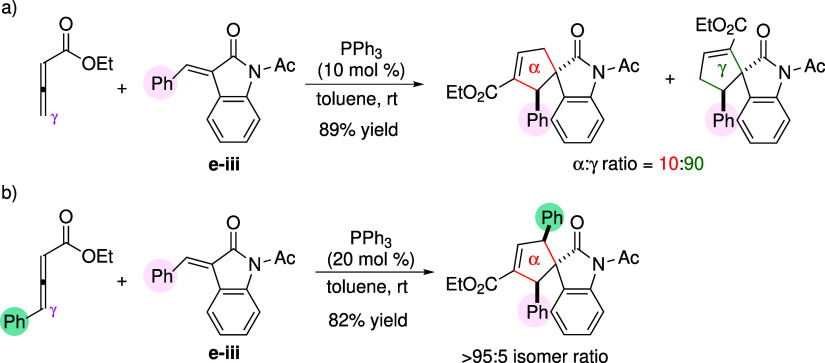
γ-Substituent Effects of Allenoates on Regioselectivity

The reactions presented in Scheme 2 were performed using unsubstituted allenoates. In addition to the substituent effect of the enone, a notable influence also arises from substitution at the γ-position of the allenoate. Scheme 3 shows a representative case in which the presence of the γ-substituent leads to an inversion of regioselectivity.^33,34^ When an unsubstituted allenoate reacted with arylideneoxindole **e-iii** shown in Scheme 3a, the γ-[3 + 2] cyclization was favored with an α:γ ratio of 10:90,^33^ in line with the regioselectivity trend observed in Scheme 2. However, introducing a phenyl group at the γ-position of the allenoate (Scheme 3b) favors the α-regioisomer as the major product, with a high isomer ratio of >95:5.^34^ These findings indicate that the γ-substituent effect of the allenoate overrides the β-substituent effect of the enone in determining product regiochemistry. Similar reversals in regioselectivity have been reported in other phosphine-catalyzed reactions involving γ-substituted allenoates and various enone substrates.^35,36^ Notably, the cyclization employing the γ-substituted allenoate also exhibited high levels of diastereoselectivity. In the major product, the two phenyl substituents were assigned as *trans* to the carbonyl group of the oxindole (Scheme 3b). This stereochemical feature has also been observed in the annulation reactions of γ-substituted allenoates and β-substituted enones.^37–39^

The mechanism for the phosphine-catalyzed [3 + 2] cycloaddition reaction has been studied through kinetic experiments and computations.^40–43^ Scheme 4 illustrates the generally accepted reaction mechanism. The catalytic cycle begins with nucleophilic addition of a phosphine to an allenoate, generating a zwitterionic adduct (**1** + **2** → **3**). Kinetic studies and ^31^P NMR monitoring have identified this initial addition as the rate-determining step.^43–45^ In regard to structural features, the adduct was proposed by Kwon to exist as interconverting *Z*- and *E*-isomers.^46^ The *Z*/*E*-isomerism was invoked to rationalize the formation of divergent products, dioxanylidenes^47^ and 6-substituted 2-pyrones,^46^ when aldehydes were used as reaction partners. The negatively charged part of the adducts shown in Scheme 4 is formally represented as an enolate, while an alternative resonance structure includes an allyl anion with a negative charge at either the α- or γ-position (referred to as a 1,3-dipole). The [3 + 2] annulation proceeds via a two-step sequence, consisting of Michael addition (**3** → **4**) followed by ring closure (**4** →**5**). In the Michael addition step, the adduct can regioselectively attack an activated alkene through either its α- or γ-carbon. Subsequent ring closure affords the five-membered phosphorus ylide **5**. Density functional theory (DFT) calculations have shown that Michael addition is the regiodetermining step.^40,42,43^ To regenerate the catalyst, the ylide undergoes [1,2] proton transfer (**5** → **6**), which was suggested to proceed through an intermolecular mechanism assisted by a trace amount of water.^41^ Finally, catalyst expulsion yields the cyclopentene products as the α- and γ-regioisomers.

**Scheme 4:**
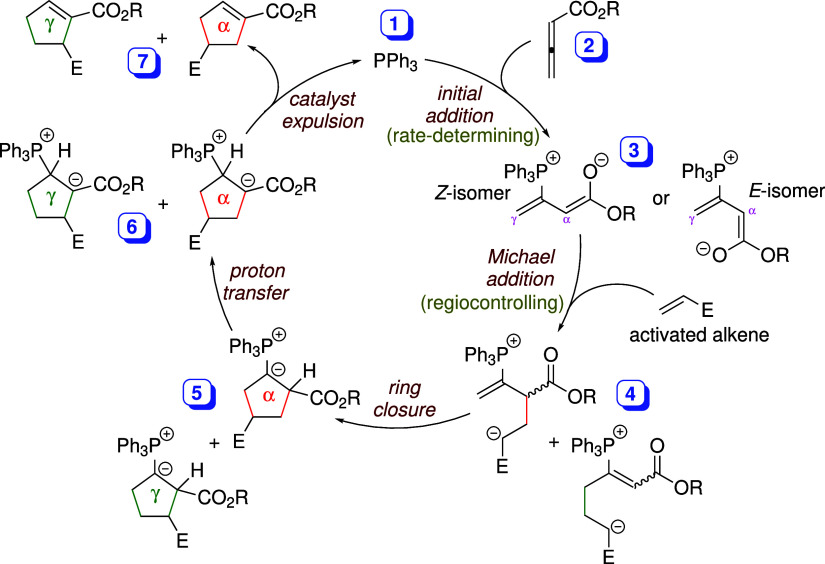
Phosphine-Catalyzed [3 + 2] Cyclization of Allenoates and Activated Alkenes

We now focus more closely on the regioselective Michael addition step, which can proceed via either the *Z*- or *E*-adduct. The extent of pre-equilibration between these two adducts is expected to influence the favorability of subsequent reactions. According to the Curtin–Hammett principle, when the starting conformers interconvert rapidly (with a pre-equilibrium established), product selectivity is governed by the difference in activation energies between the competing pathways.^48–53^ Figure 1a illustrates the free energy diagram representing the Curtin–Hammett scenario (*k*_1_, *k*_–1_ ≫ *k*_A_, *k*_B_, where *k*_1_ and *k*_–1_ denote the rate constants for interconversion, while *k*_A_ and *k*_B_ represent the rate constants for the subsequent reactions). Under this condition, the product distribution is independent of the population of the conformers and depends solely on the energy difference between the TSs of the two competing reactions (ΔΔ*G* ^‡^, depicted in Figure 1a).^50–52^ The ratio of the final products is determined by the equation shown in Figure 1a. Conversely, if the conformational exchange is slow compared to the subsequent reactions (*k*_1_, *k*_–1_ ≪ *k*_A_, *k*_B_; Figure 1b), the pre-equilibrium between the two conformers could not be established prior to reaction. The product distribution is instead determined by the relative populations of the conformers at the initial state. This scenario is termed the non-Curtin–Hammett condition (or kinetic quenching): the two conformers cannot be rapidly replenished from one another so that each species reacts independently without equilibration.^50,51^ Thus, a deep understanding of the regiochemical outcome of the [3 + 2] cyclization necessitates careful evaluation of the relative rates of adduct interconversion and the subsequent Michael addition step.

**Figure 1:**
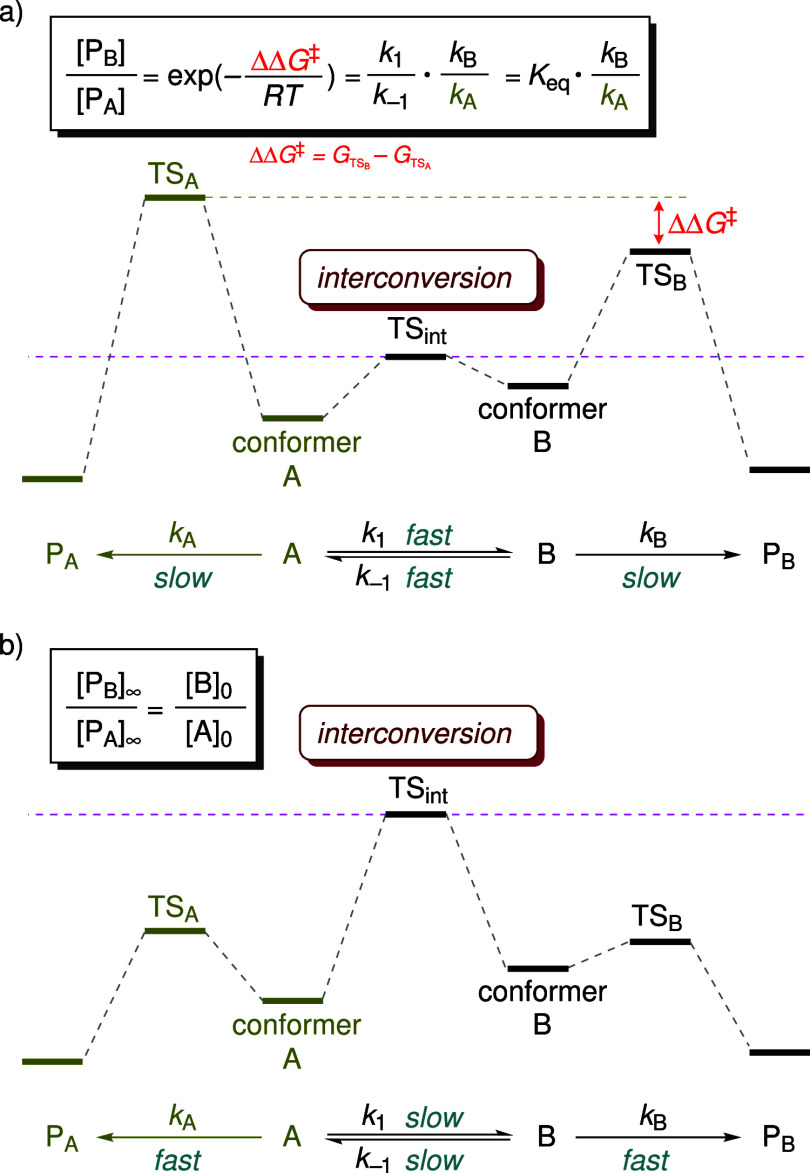
Free energy diagrams for (a) the Curtin–Hammett, and (b) the non-Curtin–Hammett scenarios.

Although the mechanism of the phosphine-catalyzed [3 + 2] cyclization has been extensively investigated,^41–43,54^ the origin of substituent-controlled regioselectivity, illustrated in Schemes 2 and 3, is still unclear. Furthermore, the mechanistic aspects of the *Z*/*E* isomerism^46^ in the adducts (originally proposed by Kwon to rationalize product selectivity) remain insufficiently explored in reactions involving other substrates such as enones. Therefore, the pathways diverging from the *Z*- and *E*-adducts should be considered in the catalytic mechanism of the [3 + 2] cycloaddition reaction. Once the *Z*/*E* isomerism is taken into account, the relative favorability of adduct interconversion versus cycloaddition could influence product selectivity, as illustrated in Figure 1. To our knowledge, the role of the *Z*/*E* isomerism in influencing regioselectivity has not been clearly elucidated. To address these key points, theoretical calculations and kinetic modeling are performed for the two reactions shown in Scheme 3. Kinetic modeling is particularly advantageous because it considers both rate constants and concentration effects simultaneously, enabling clearer identification of the dominant reaction pathway in complex network systems. Methyl allenoate is first employed as the substrate to cyclize with arylideneoxindole **e-iii**. Previous studies primarily employed PMe_3_ as a model catalyst.^40–43,54^ However, our preliminary calculations showed that the use of PMe_3_ limited the exploration of pathways leading to other isomeric adducts (see Scheme S1 in the ESI). To more closely reflect experimental conditions, the initial addition process is re-evaluated using PPh_3_. In the regioselective Michael addition step, both α- and γ-addition modes, derived from the *Z*- and *E*-adducts, are examined. The distortion/interaction activation strain model is applied to analyze these regioisomeric transition states (TSs).^55^ Subsequently, kinetic simulations based on the DFT-computed energetics are conducted to estimate the product ratio of α- to γ-regioisomers and to compare these results with experimental observations. The same procedure is then carried out on the annulation depicted in Scheme 3b to investigate substituent effects. Unlike the unsubstituted allenoate, the γ-substituted allenoate can form a greater number of adduct isomers, and possible reaction pathways are systematically analyzed. Further, the factors responsible for the reversal in regioselectivity and the observed *trans*-stereochemistry of the product are identified. Finally, we present a comprehensive picture of the kinetic interplay between adduct interconversion and Michael addition (modulated by substituent effects), delineating the conditions under which the Curtin–Hammett principle applies.

## 2 Results and Discussion

### 2.1 Reactions of Unsubstituted Allenoates

#### 2.1.1 Adduct Formation

We first explore the catalytic mechanism of the [3 + 2] cyclization between unsubstituted allenoate **2** and arylideneoxindole **e-iii**, shown in Scheme 3a. The catalytic cycle is initiated by nucleophilic addition of PPh_3_ to the allenoate. Calculations indicate that the initial addition can proceed through three isomeric pathways, as illustrated in Scheme 5a. The computed free energy profile is depicted in Scheme 5b, where the energies marked with double daggers (‡) represent the activation free energies. In addition to the previously reported *Z*- and *E*-configured adducts (denoted as **3-Z** and **3-E**, respectively), another intermediate, **3-int**, is identified. Structurally, **3-int** closely resembles **3-Z** (Figure 2), but with a key distinction: in **3-int**, the enolate moiety is twisted out of conjugation with the Cβ=Cγ double bond, with a C1–Cα–Cβ–Cγ dihedral angle of 140° (compared to 179° in **3-Z**). The twisted geometry similar to **3-int** was proposed in Lu’s early studies^56–58^ as an intermediate in allenoate-associated phosphine catalysis, although it received limited attention in discussions of the reaction mechanism thereafter.^59^

**Scheme 5:**
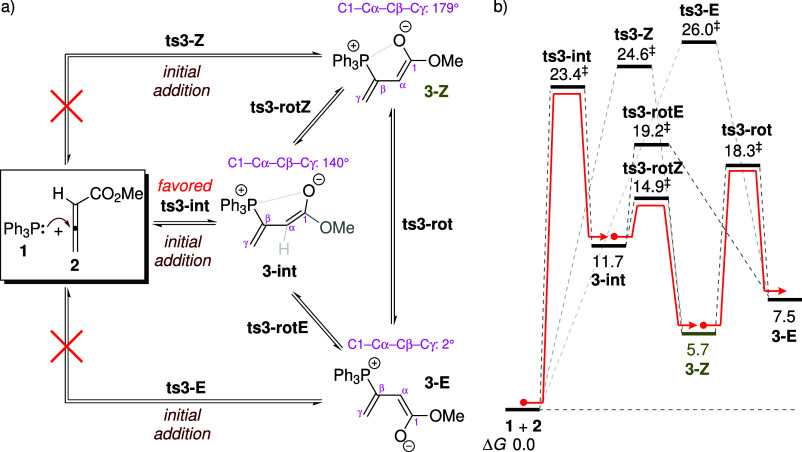
(a) Three Reaction Pathways for the Addition of PPh_3_ to Unsubstituted Allenoate **2**, (b) Free energy profiles (Δ*G* in kcal mol^–1^)­^a^

a The reported energies are relative to the energy of the separated reactants. The red line indicates the energetically preferred pathway leading to *Z*- and *E*-adducts.

**Figure 2:**
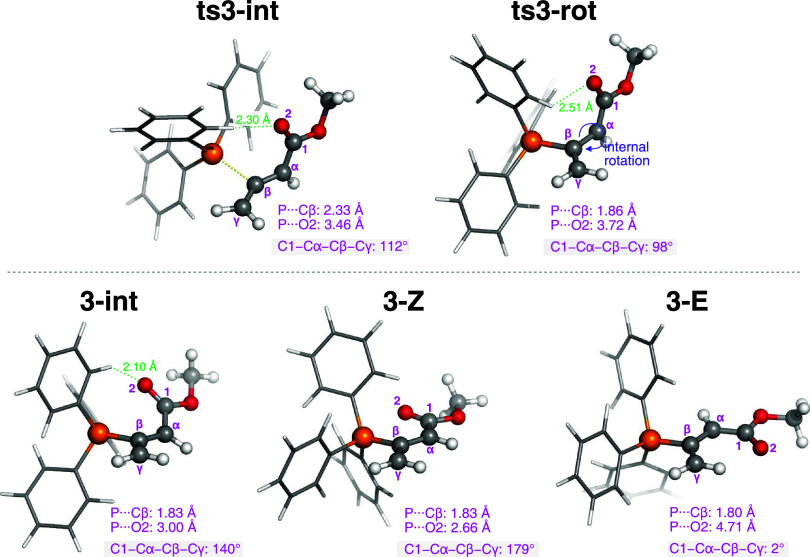
Optimized structures for adduct formation. Selected structural parameters are listed in magenta. The noncovalent interactions of CH···O are represented by green dashed lines.

Among the three possible pathways, formation of the twisted adduct is calculated to be the most energetically favored, exhibiting the lowest activation barrier of 23.4 kcal mol^–1^ (**ts3-int**). In contrast, the generation of the *Z*- and *E*-adducts requires higher activation energies of 24.6 and 26.0 kcal mol^–1^ (**ts3-Z** and **ts3-E**), respectively. The electrostatic interaction between the phosphorus center and the carbonyl oxygen is generally regarded as a key stabilizing factor in the initial addition step.^41,43,54,60^ Structural analysis shows that the favored TS **ts3-int** features the shortest P···O distance at 3.46 Å (Figure 2), compared to 3.56/4.31 Å in **ts3-Z**/**ts3-E** (Figure S7 in the ESI). In addition to electrostatic effects, noncovalent interactions involving the phenyl group of the catalyst are also observed: **ts3-int** shows a CH···O contact distance of 2.30 Å, shorter than 2.40/2.44 Å observed in **ts3-Z**/**ts3-E** (see Figures S7 and S8). This close contact further contributes to the stabilization of **ts3-int**.

Because the formation of all three adducts is endergonic, they are expected to exist as fleeting intermediates, consistent with ^31^P NMR spectroscopy showing that the predominant resting state of the catalyst is the free phosphine.^44,45^ The adducts follow a relative stability trend of **3-Z** > **3-E** > **3-int**, with **3-Z** being the most stable and **3-int** the least stable. The electron localization function^61^ analysis of the adducts reveals that the bonding patterns of the four atoms C1, Cα, Cβ, and Cγ are similar to those observed in a diene system (Figure S6 in the ESI). The poor stability of the twisted adduct is thus suggested to arise from weak resonance stabilization between the C1=Cα and Cβ=Cγ double bonds. In contrast, both **3-Z** and **3-E** adopt a near-coplanar arrangement of these two double bonds (Figure 2), which enhances their stability. A key factor differentiating the stability between **3-Z** and **3-E** (ΔΔ*G*: 1.8 kcal mol^–1^) is the presence of the stabilizing P···O interaction in the former. Notably, the three adduct isomers can interconvert through the internal rotation with respect to the phenyl ring and the enolate moiety. The TSs with respect to internal rotation (**ts3-rotZ**, **ts3-rotE**, and **ts3-rot**), whose energies range from 14.9 to 19.2 kcal mol^–1^, are significantly lower in free energy than **ts3-int** (23.4 kcal mol^–1^). Therefore, both **3-Z** and **3-E** can be accessed through conformational equilibration from **3-int**, bypassing the higher-energy TSs **ts3-Z** and **ts3-E**. The energetically favored pathway leading to the *Z*- and *E*-adducts is highlighted by the red line in Scheme 5b. Specifically, the generation of **3-E** proceeds primarily through interconversion from **3-Z** (via the rotational TS **ts3-rot**, shown in Figure 2).

#### 2.1.2 Michael Addition

Intrinsic reaction coordinate^62^ (IRC) calculations confirm that the low-energy TSs associated with Michael addition are connected to either adduct **3-Z** or **3-E**, rather than to **3-int**. Accordingly, the following analysis of the [3 + 2] cyclization focuses on the reaction of **3-Z** and **3-E** with the enone. Previous theoretical investigations highlighted two key factors that stabilize the regiocontrolling TSs: (i) frontier orbital overlap between the 1,3-dipole and the alkene, and (ii) noncovalent interactions between the catalyst moiety and the carbonyl oxygen atom of the allenoate.^40,42,43^ Guided by these features, four isomeric TSs shown in Figure 3 are examined: **ts4-Za** and **ts4-Zg** involve the α- and γ-mode Michael addition of **3-Z**, respectively, while **ts4-Ea** and **ts4-Eg** arise from **3-E**.

**Figure 3:**
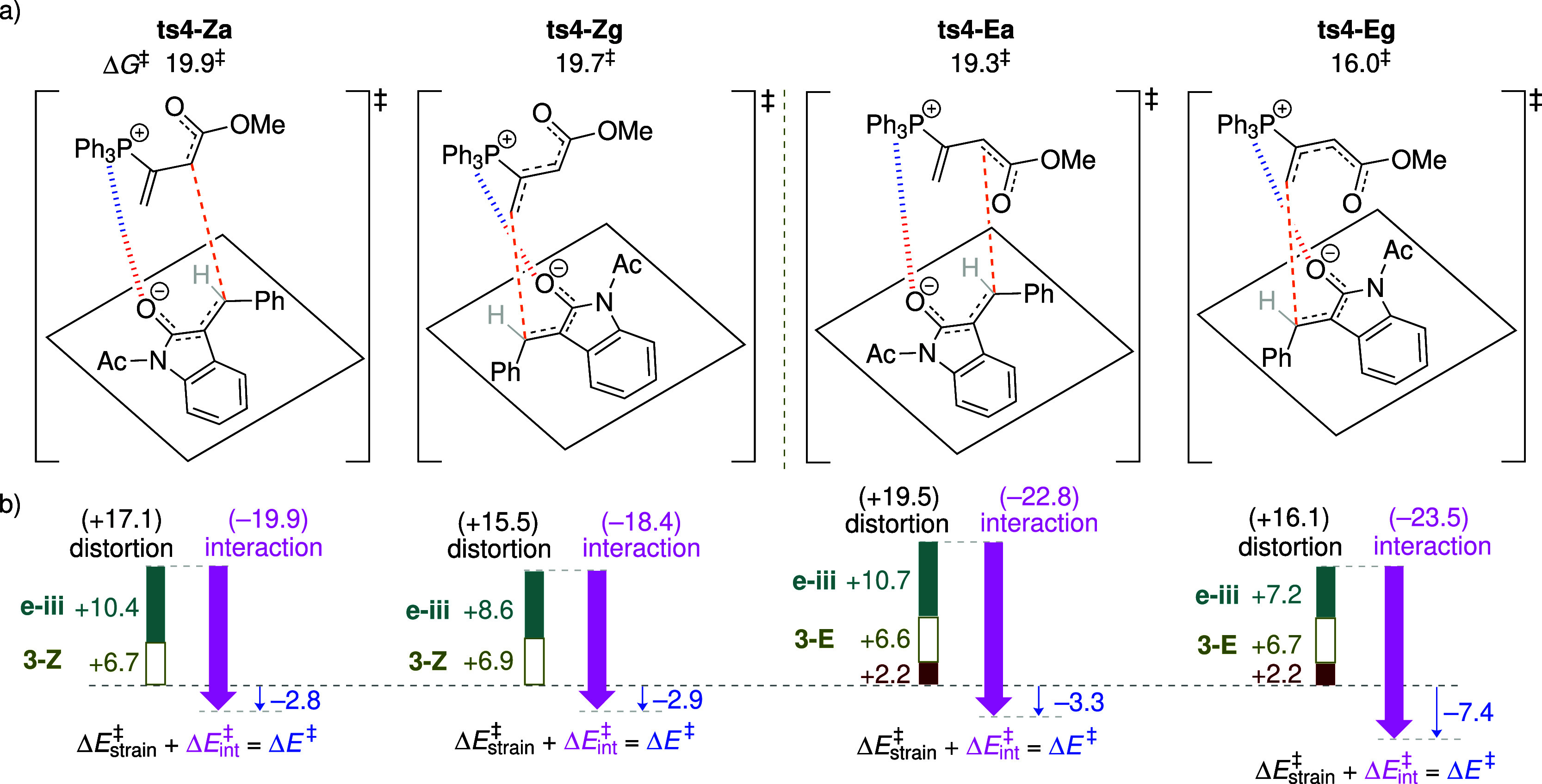
(a) Four isomeric TSs for the reaction of **3-Z**/**3-E** with enone **e-iii**. Computed activation free energies are relative to the energy of the separated reactants. The bond being formed is indicated by orange dashed lines, while the Ph_3_P···O interaction is shown as blue-red hashed lines. (b) Analysis of the distortion-interaction model based on electronic energies. All energies are given in units of kcal mol^–1^.

Calculations show that the relative ordering of free energies and enthalpies among the four Michael addition modes consistently follows the trend: **ts4-Eg** < **ts4-Ea** ≲ **ts4-Zg** ≲ **ts4-Za** (Figure S12). In terms of free energy, **ts4-Eg** is at least 3.3 kcal mol^–1^ lower than the other three TSs (Figure 3a). To elucidate the origin of the particularly low energy of **ts4-Eg**, the distortion/interaction model (based on electronic energies) is employed to analyze the four isomeric TSs. In this model, the activation energy is partitioned into distortion energy and interaction energy: Δ*E* ^‡^ = Δ*E*_strain_^‡^ + Δ*E*_int_^‡^. The distortion energy (Δ*E*_strain_^‡^) is the energy required to deform the equilibrium structure of the two reacting species into the TS geometry, while the interaction energy (Δ*E*_int_^‡^) is defined as the difference between the activation energy and the distortion energy. Taking **3-Z** as the reference point, the electronic energy difference of 2.2 kcal mol^–1^ between **3-Z** and **3-E** is included as part of the distortion energy in pathways involving **3-E**. The distortion energies in the four TSs **ts4-Za**, **ts4-Zg**, **ts4-Ea**, and **ts4-Eg** are +17.1, + 15.5, + 19.5, and +16.1 kcal mol^–1^, respectively (Figure 3b). In **ts4-Eg**, the moderate distortion energy is compensated by the strongest interaction energy of −23.5 kcal mol^–1^, rendering it the most favorable TS among the four examined. To gain deeper insight into the factors underlying this strong interaction, the frontier molecular orbitals (FMOs) of the adduct and enone are examined in terms of their HOMO–LUMO energy gap and orbital overlap. Because the HOMO of **3-E** (−6.07 eV) is higher in energy than that of **3-Z** (−6.22 eV), the reaction involving **3-E** benefits from a smaller HOMO–LUMO gap. Furthermore, the FMO analyses of the reacting species and the TS are conducted to recognize the pattern of orbital overlap (see Figure S11 in the ESI), and the identified orbital interactions are schematically depicted in Figure 4a. The primary orbital overlap occurs between the allenic moiety and the alkene of the enone. The secondary orbital interaction is observed between the enolate and the oxindole ring, which explains their nearly parallel alignment. The other three disfavored TSs, in contrast, are unable to engage in this type of second interaction. The FMO analyses thus suggest that the secondary orbital interaction plays a significant role in stabilizing **ts4-Eg**. Similar effects have also been proposed to explain the preference for *E*-dihydropyran products in the amine-catalyzed [4 + 2] annulation of allenoates with enones.^54^ In addition, two noncovalent CH···O contacts are identified in **ts4-Eg**: one is formed between the O5 atom and the phenyl group of the catalyst (*d*_CH···*O*5_ = 2.08 Å; Figure 4a), and the other between the O2 atom and the β-phenyl group (*d*_CH···*O*2_ = 2.43 Å). These noncovalent interactions are also likely to provide additional stabilization.

**Figure 4:**
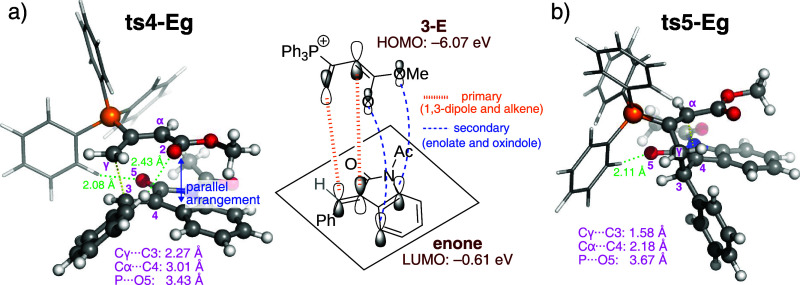
TS structures of (a) Michael addition and (b) ring closure, where the noncovalent interactions of CH···O are represented by green dashed lines. In **ts4-Eg**, the primary and secondary orbital interactions are depicted by orange and blue dashed lines, respectively.

#### 2.1.3 Ring Closure

During the cyclization, the γ-mode of Michael addition (via **ts4-Eg**) generates enolate **4-Eg**, as shown in Figure 5a. Subsequent ring closure proceeds with facial selectivity. If rotation around the Cα–Cβ bond is feasible in the enolate intermediate, the two carbon–carbon bond forming events in the [3 + 2] cyclization could occur on opposite faces of the alkene, resulting in *anti*-cycloaddition. However, the *anti* mode is calculated to be 8.3 kcal mol^–1^ higher in energy than the *syn* mode. This energetic penalty arises from repulsive electrostatic interactions between the ester group and the oxindole carbonyl oxygen, indicated by the electrostatic potential surface in Figure S13. Moreover, the noncovalent CH···O5 interaction observed in the *syn* TS (*d*_CH···*O*5_: 2.11 Å; Figure 4b) is absent in the disfavored *anti* mode. The combined effects of these factors thus render *syn* addition more favorable. Formation of ylide **5-Eg** is exergonic by 8.2 kcal mol^–1^ (Figure 5a), making the [3 + 2] annulation practically irreversible. The ylide then undergoes proton transfer followed by catalyst expulsion to complete the catalytic cycle. The final cyclic product (**7-Eg**) is 24.1 kcal mol^–1^ more stable than the ylide, and the overall reaction is highly exergonic by 32.3 kcal mol^–1^. The rate-determining step is the attack of the phosphine catalyst on the allenoate (with the highest activation energy of 23.4 kcal mol^–1^), in line with experimental observations.^43–45^

**Figure 5:**
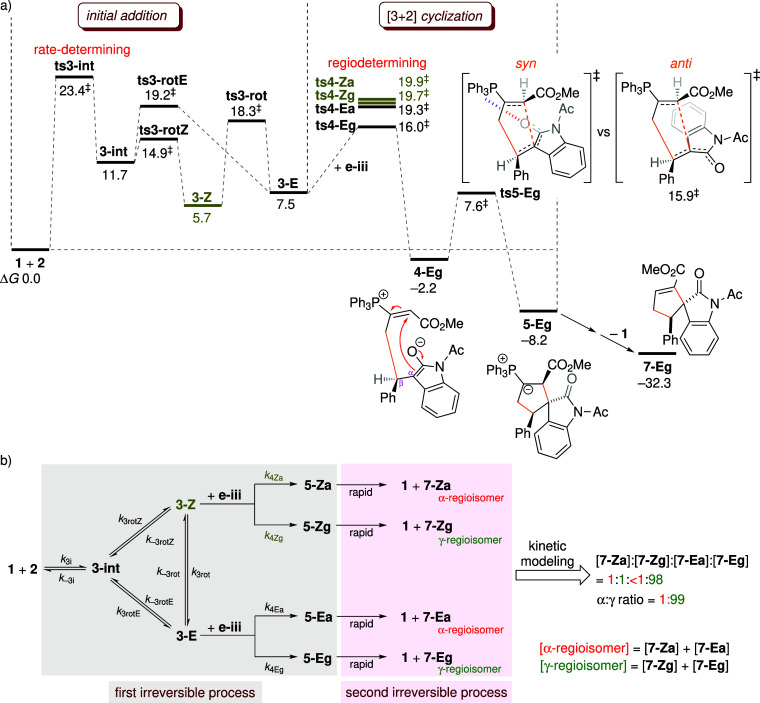
(a) Free energy profile (Δ*G* in kcal mol^–1^) for the [3 + 2] cycloaddition of allenoate **2** with enone **e-iii**. (b) Reaction network and the calculated α:γ product ratio.

#### 2.1.4 Kinetic Modeling

To clarify the kinetic scenario depicted in Figure 1, the key barrier heights for adduct interconversion and Michael addition are organized for comparison in Figure 6a. Only **ts4-Eg** lies lower in energy than **ts3-rot** (the TS for adduct interconversion), whereas **ts4-Za**, **ts4-Zg**, and **ts4-Ea** are energetically higher. This places the kinetic system in an intermediate regime between Curtin–Hammett and non-Curtin–Hammett control. The *E*-adduct is primarily formed from the *Z*-adduct (Scheme 5b) so that prior adduct interconversion is required for the cyclization of the *E*-adduct to proceed. To verify that the [3 + 2] cyclization proceeds predominantly via **ts4-Eg**, kinetic modeling is performed based on the reaction network shown in Figure 5b. Since the formation of the ylide is exergonic and practically irreversible, the catalytic cycle can be simplified into two irreversible stages: the generation of the ylide (**1** + **2** + **e-iii** → **5-Eg**) and its subsequent conversion to the final product (**5-Eg** → **1** + **7-Eg**). The first stage comprises initial addition and [3 + 2] annulation, while the second includes proton transfer and catalyst expulsion. The rate constant for the regioselective cyclization is approximated by that of the Michael addition step, as this step dictates the regiochemical outcome of the reaction. Given that the initial addition is rate-limiting, it is reasonable to assume that the second irreversible process is relatively fast. The initial concentrations of **1**, **2**, and **e-iii** are set to 0.03, 0.60, and 0.30 M, respectively, based on experimental conditions with a catalyst loading of 10 mol %.^33,34^ The kinetic simulation predicts a 1:1:<1:98 ratio for the products generated via the four regioselective TSs: **ts4-Za**, **ts4-Zg**, **ts4-Ea**, and **ts4-Eg**. The α-regioisomer is formed via **ts4-Za** and **ts4-Ea**, whereas the γ-regioisomer arises from **ts4-Zg** and **ts4-Eg**. The computed α:γ ratio of 1:99 is in reasonable agreement with the experimental value of 10:90.^33^ The kinetic modeling demonstrates that the major γ-product is generated through the [3 + 2] cycloaddition of the *E*-adduct with the enone (accounting for 98% of the product distribution).

**Figure 6:**
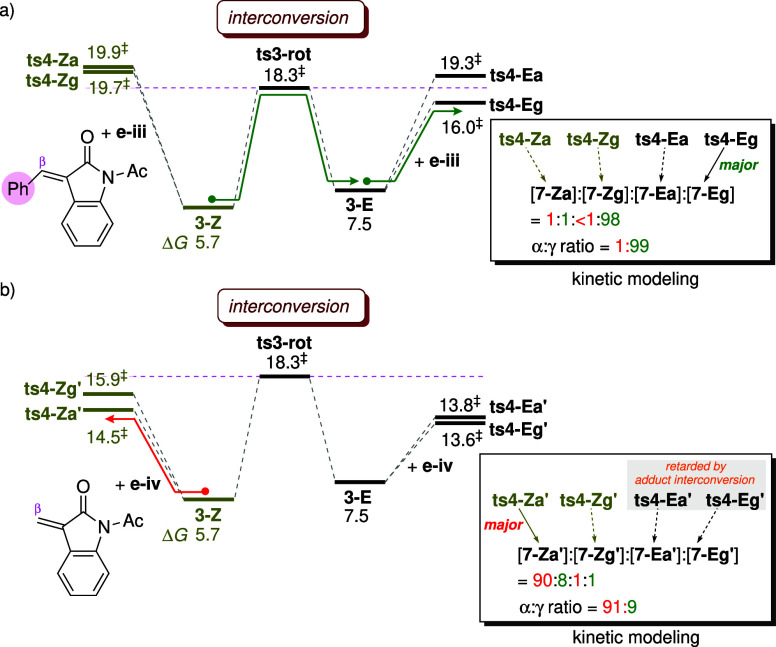
Comparison of activation energies (kcal mol^–1^) for adduct isomerization and Michael addition. Reported free energies are relative to the energy of the separated reactants. The α:γ ratios computed from kinetic modeling are provided in the box on the right.

It is important to note that neglecting the cycloaddition pathway derived from **3-E** leads to incorrect predictions of regioselectivity. If the reaction were assumed to proceed exclusively through **3-Z**, the small energy difference of 0.2 kcal mol^–1^ between **ts4-Za** and **ts4-Zg** would yield an α:γ ratio of 42:58, indicating poor selectivity and deviating markedly from the experimental outcome. To rationalize the high regioselectivity observed in experiments, inclusion of the pathway involving **3-E** in the catalytic mechanism is therefore essential.

#### 2.1.5 β-Substituent Effects in Enones

As shown in Scheme 2, when the enone lacks a phenyl group at the β-position, the α-regioisomer is obtained as the major product. To probe the substituent effect, the model substrate **e-iv** (Figure 6b), which lacks the β-phenyl group, is used to react with the *Z* and *E*-adducts. The four Michael addition modes (**ts4-Za′**, **ts4-Zg′**, **ts4-Ea′** and **ts4-Eg′**) analogous to those in Figure 3 are examined (see Figure S14 in the ESI). Although experimental studies using enone **e-iv** have not been reported, our computational study can provide useful implications regarding the influence of the β-substituent on reaction energetics. In the absence of the β-substituent, the activation barriers for the four addition modes decrease to 14.5, 15.9, 13.8, and 13.6 kcal mol^–1^, respectively (Figure 6b). This consistent reduction in barrier heights indicates that removal of the β-phenyl group enhances the reactivity of the enone. Because the LUMO energy of **e-iv** (−0.41 eV) is higher than that of **e-iii** (−0.61 eV), this enhanced reactivity is not attributed to orbital interactions but is instead likely due to reduced steric hindrance during Michael addition. Among the divergent pathways from **3-Z** and **3-E**, the former exhibits higher activation barriers for the Michael addition. Nevertheless, because the activation energies for all four addition modes lie below the barrier for adduct interconversion (Δ*G* ^‡^: 18.3 kcal mol^–1^), the system is expected to operate under non-Curtin–Hammett control. To validate that the system follows the non-Curtin–Hammett scenario, kinetic modeling is conducted using the reaction network shown in Figure 5b, with the activation energies for the four Michael addition modes replaced by those calculated for **e-iv**. The simulation yields a product distribution of 90:8:1:1 (Figure 6b). Although Michael addition of **3-E** exhibits lower activation barriers than that of **3-Z**, this pathway is kinetically suppressed because its preceding step involves slow equilibration from **3-Z** to **3-E**. As a result, the cycloaddition reaction via **3-Z** accounts for nearly all of the product formation (90% + 8%).

Since the pathway involving **3-E** is blocked under kinetic quenching conditions, regioselectivity is determined by the two competing TSs, **ts4-Za′** and **ts4-Zg′**, derived from **3-Z**. The origin of the preference for α-addition via the *Z*-adduct has been previously elucidated in computational studies on the annulation of methyl allenoates and acrylates using PMe_3_ as the catalyst.^40,43^ Kwon and co-workers proposed that interactions between the carbonyl oxygen atom of the enone and the catalyst moiety, such as P···O interactions and CH···O hydrogen bonding, played a critical role in directing regioselectivity.^40^ The Yu group attributed α-selectivity to favorable orbital interactions.^43^ An alternative explanation based on the distortion/interaction model also offers a distinctive perspective on regiocontrol.^63,64^ In the current system, the α-addition pathway exhibits a lower distortion energy (+8.8 vs +11.2 kcal mol^–1^; see Figure S14), which offsets its slightly weaker interaction energy (−15.7 vs −16.5 kcal mol^–1^). The regioselectivity in the reaction between **3-Z** and **e-iv** is therefore suggested to be governed by distortion energy.

### 2.2 Reactions of γ-Substituted Allenoates

In the following sections, we further investigate the origin of the reversed regioselectivity observed when a phenyl group is attached to the γ-position of the allenoate, as illustrated in Scheme 3b. Unlike unsubstituted allenoates, γ-substituted allenoates generate more diverse adduct isomers. To explore these isomeric pathways, γ-phenyl allenoate **8** is selected to react with PPh_3_ (Scheme 6). Since **8** is axially chiral, its (*R*)-configured form is selected as the representative structure for computational studies.

**Scheme 6:**
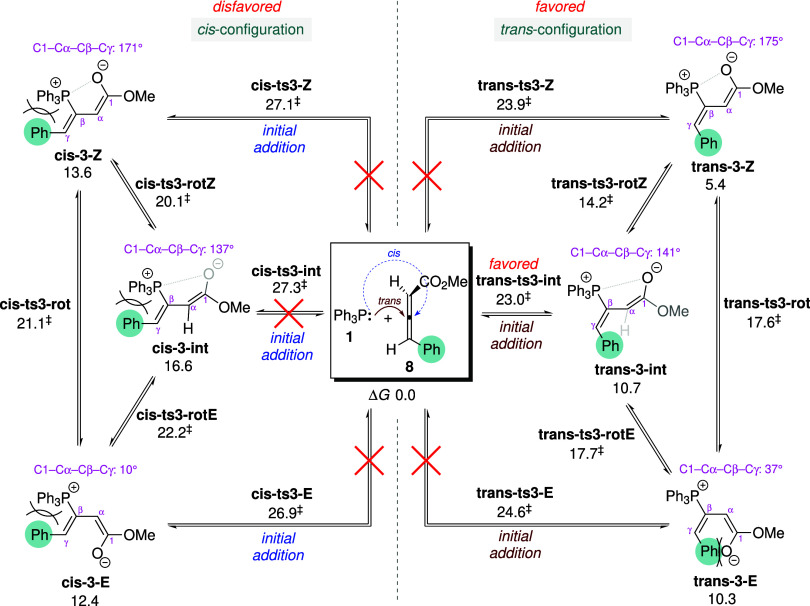
Six Reaction Pathways for Addition of PPh_3_ to γ-Substituted Allenoate **8** ^a^

a Computed free energies (Δ*G* in kcal mol^–1^) are relative to the energy of the separated reactants.

#### 2.2.1 Torquoselectivity in Adduct Formation

On the basis of the preceding studies with unsubstituted allenoate **2**, six possible adduct isomers formed from **8** are identified and classified into *cis* and *trans* subsets according to the geometric orientation of the γ-phenyl group (Scheme 6). These two adduct subsets arise from torquoselective inward and outward rotation of the γ-substituent relative to the catalyst moiety during adduct formation. The preferred mode of rotation plays a decisive role in setting the stereochemistry of the γ-substituent in the final product. In the three *cis*-configured adducts (**cis-3-Z**, **cis-3-E**, and **cis-3-int**, depicted on the left side of Scheme 6), the phosphonium and the γ-phenyl groups reside on the same side of the Cβ=Cγ double bond, whereas in the three *trans*-isomers (**trans-3-Z**, **trans-3-E**, and **trans-3-int**), they are on opposite sides. The *Z* and *E* notations still follow the previous designations, referring to the arrangement of the ester group relative to the phosphonium moiety. Attempts to locate the TS with respect to interconversion between the *trans*- and *cis*-isomers were unsuccessful, due to the geometric rigidity imposed by the Cβ=Cγ double bond. Therefore, adduct isomerization is restricted to occur only within the *cis*- or *trans*-adduct subsets, with no crossover between them. All optimized geometries for nucleophilic attack of PPh_3_ on **8** are provided in Figures S15 and S16 of the ESI, while representative structures are depicted in Figure 7.

**Figure 7:**
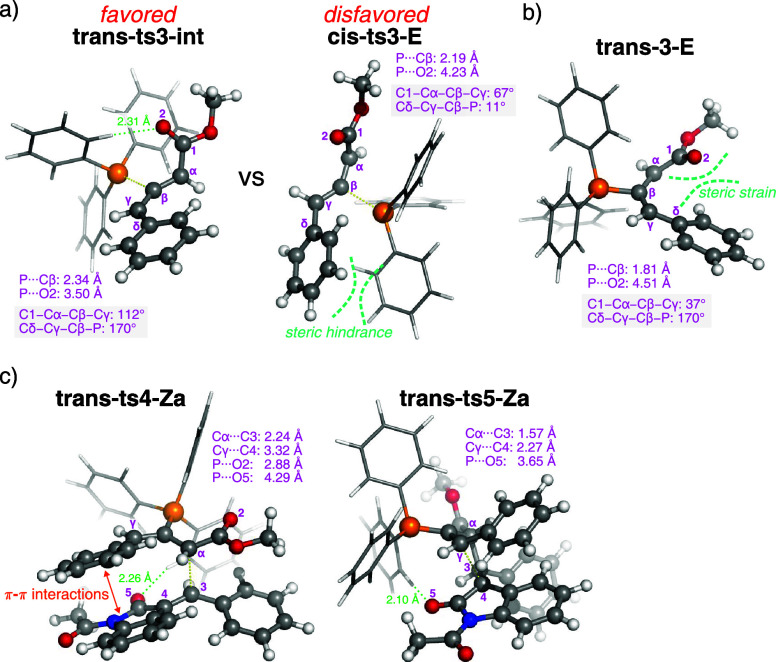
(a) Structural comparison of **trans-ts3-int** and **cis-ts3-E**, which control torquoselectivity. (b) Geometry of the *E*-adduct **trans-3-E**. (c) TS structures for Michael addition (**trans-ts4-Za**) and ring closure (**trans-ts5-Za**). The noncovalent interactions of CH···O are represented by green dashed lines.

Among the three pathways leading to the *trans*-adducts, the most favorable proceeds via **trans-ts3-int**, with an activation barrier of 23.0 kcal mol^–1^ (Scheme 6). Examination of the calculated geometries shows that the structural features of the *trans*-configured TSs/adducts are largely similar to those formed from the unsubstituted allenoate **2** (cf. Figure S7 with Figure S15), suggesting that outward rotation of the γ-substituent does not experience significant steric hindrance from the catalyst moiety. The only notable deviation is observed for **trans-3-E**, in which the enolate moiety is not coplanar with the Cβ=Cγ double bond, with a C1–Cα–Cβ–Cγ dihedral angle of 37° (Figure 7b). The nonplanarity arises from steric strain between the enolate and the γ-phenyl substituent. This steric effect leads to a larger energy gap of 4.9 kcal mol^–1^ between **trans-3-Z** and **trans-3-E**, compared to only 1.8 kcal mol^–1^ between **3-Z** and **3-E** derived from unsubstituted allenoate **2**. Among the *cis*-configured pathways, the lowest-energy TS is **cis-ts3-E** (Δ*G* ^‡^: 26.9 kcal mol^–1^), which is 3.9 kcal mol^–1^ higher in energy than **trans-ts3-int**. Steric clash arising from inward rotation of the γ-substituent toward the catalyst moiety accounts for the disfavored *cis*-configuration (Figure 7a). Given that the initial addition is rate-determining, the *trans*-adducts are formed more rapidly than their *cis*-counterparts and thus dominate as major intermediates in the ensuing cycloaddition with enones. Accordingly, the following discussions focus on the reaction pathways that begin from the two *trans*-adducts, **trans-3-Z** and **trans-3-E**, which correspond to **3-Z** and **3-E** in the case of unsubstituted allenoate **2**. It is also noteworthy that the (*R*)- and (*S*)-configurations of the γ-substituted allenoate do not alter the torquoselectivity toward *trans*-adduct formation, which implies that the chirality of the allenic molecule is lost upon reaction with PPh_3_.

#### 2.2.2 Regioselective Michael Addition

Four regioisomeric TSs, analogous to those depicted in Figure 3, are examined for the Michael addition step (Figure 8): **trans-ts4-Za** and **trans-ts4-Zg** involve α- and γ-mode addition of the adduct **trans-3-Z** to the enone, respectively, while **trans-ts4-Ea** and **trans-ts4-Eg** similarly arise from **trans-3-E**.

**Figure 8:**
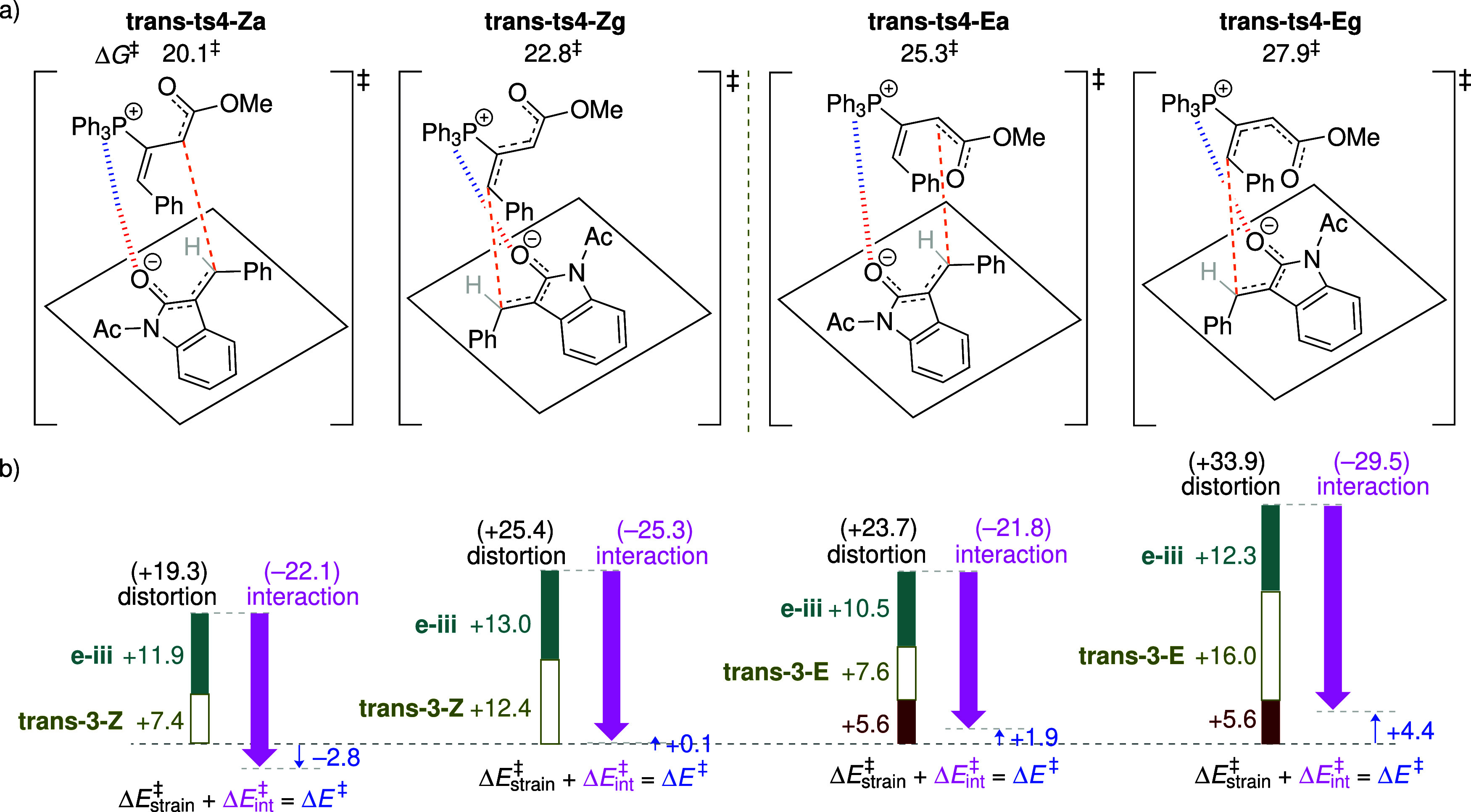
(a) Four isomeric TSs for the reaction of **trans-3-Z**/**trans-3-E** with enone **e-iii**. Computed activation free energies are relative to the energy of the separated reactants. (b) Analysis of the distortion-interaction model based on electronic energies. All energies are given in units of kcal mol^–1^.

Because the steric repulsion between the γ-substituent and the enolate destabilizes the *E*-adduct relative to the *Z*-isomer, this unfavorable effect persists throughout the subsequent Michael addition step (see Figure S17), thereby disfavoring the pathways involving **trans-3-E**. Consequently, Michael addition proceeding via the *Z*-adduct becomes energetically preferred, with lower activation energies (Δ*G* ^‡^: 20.1 and 22.8 kcal mol^–1^; Figure 8a) compared to those from the *E*-adduct (Δ*G* ^‡^: 25.3 and 27.9 kcal mol^–1^). This trend contrasts with that observed for unsubstituted allenoate **2**, where Michael addition of the *E*-adduct is favored. The distortion/interaction analysis demonstrates that the preferred TS **trans-ts4-Za** benefits from the smallest distortion energy of +19.3 kcal mol^–1^ (Figure 8b), combined with a moderate interaction energy of –22.1 kcal mol^–1^. It is also noted that although the γ-addition mode of **trans-ts4-Eg** exhibits the strongest interaction energy of −29.5 kcal mol^–1^ (due to orbital interactions), this advantage is outweighed by a substantial distortion penalty of +33.9 kcal mol^–1^. The computational analysis indicates that the regioselectivity in this system is primarily controlled by distortion energy. Additionally, a particularly noteworthy geometric feature in the favored TS **trans-ts4-Za** is the parallel alignment between the γ-phenyl and oxindole rings (Figure 7c), which suggests a π–π stacking interaction. While the π–π interaction may be viewed as an alternative manifestation of the secondary orbital interaction described in Figure 4a, it contributes only modestly to the overall stabilization (as demonstrated by the distortion/interaction analysis showing that the case is not interaction-controlled). In experiments, replacing the γ-phenyl substituent with alkyl groups maintained diastereomeric ratios up to 88:12, which indicates that the π–π interaction plays a minor role in governing product selectivity, despite the decrease in overall yield.^34^ The reduced yield likely reflects a slower reaction rate (related to the rate-determining initial addition step). This interpretation is supported by kinetic measurements showing that PPh_3_ adds to γ-methyl allenoates 2.4-fold more slowly than to γ-phenyl allenoates.^6^

#### 2.2.3 Stereochemistry of Products

The preferred mode of Michael addition involves the α-attack of **trans-3-Z** to the enone, generating intermediate **trans-4-Za** (Figure 9a). Facial selectivity in the [3 + 2] cyclization is further explored by examining the ring-closure step in both *syn* and *anti* configurations. The *syn* pathway is calculated to be favored by 4.6 kcal mol^–1^ over the *anti* pathway. Structural comparison of the two TSs with respect to facial selectivity reveals a key stabilizing CH···O interaction present in the *syn* TS **trans-ts5-Za** (Figures 7c and S17), which is also observed in **ts5-Eg** (Figure 4b). In addition to its previously recognized role in the regioselective step,^40,43^ the CH···O interaction is also suggested to serve as a stereocontrolling element that steers the cyclization toward *syn* selectivity. Under both regio- and stereocontrol, the cyclization finally produces the ylide intermediate **trans-5-Za** (Figure 9), in which the two phenyl groups on the forming five-membered carbocycle are *trans* to the carbonyl group of the oxindole unit. The stereochemical configuration is preserved in the final product **trans-7-Za**, in agreement with the experimentally observed stereochemistry.^34^

**Figure 9:**
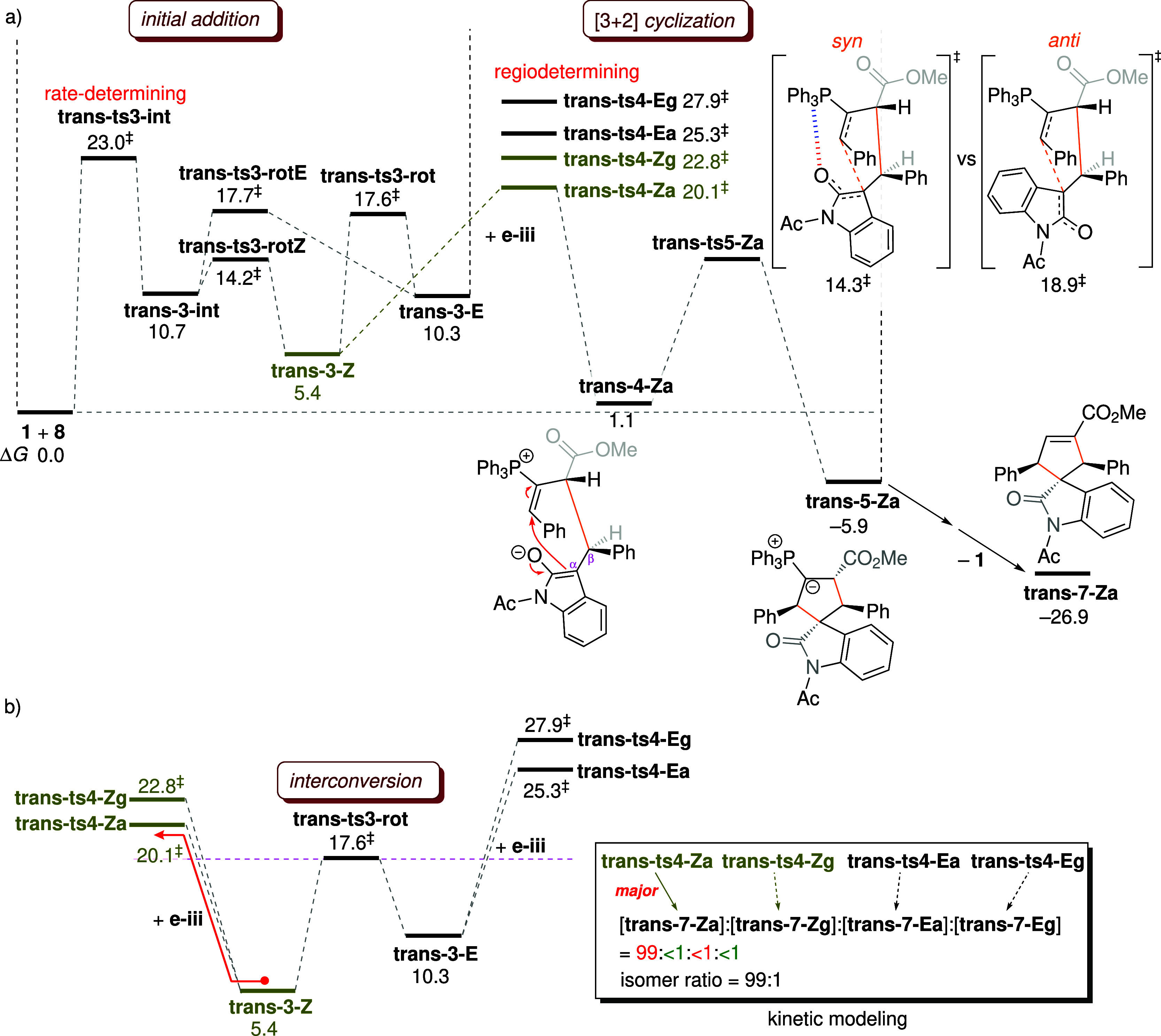
(a) Free energy profile for the [3 + 2] cycloaddition of allenoate **8** with enone **e-iii**. (b) Comparison of activation energies for adduct interconversion and Michael addition. The isomer ratio computed from the kinetic simulation is provided in the box on the right. All energies are given in units of kcal mol^–1^.

According to the comparison of the key barriers shown in Figure 9b, the selectivity is expected to follow Curtin–Hammett kinetics. To verify this, kinetic modeling is performed using the computed free energies and the initial concentrations of [**1**]_0_ = 0.20 M, [**8**]_0_ = 1.00 M, and [**e-iii**]_0_ = 1.50 M employed in experiments. The distribution of products generated from the four TSs **trans-ts4-Za**, **trans-ts4-Zg**, **trans-ts4-Ea**, and **trans-ts4-Eg** is calculated to be 99:1:<1:<1, which confirms that nearly all of the product arises from the lowest-energy TS **trans-ts4-Za** (under Curtin–Hammett control). The computed 99:1 isomer ratio agrees with the experimental value of >95:5.^34^ During catalysis, product stereochemistry is initially established during nucleophilic attack of the phosphine on the allenoate (namely, the rate-limiting step), wherein torquoselective stereocontrol directs formation of the *trans*-configured adducts. The subsequent [3 + 2] annulation proceeds under Curtin–Hammett conditions: α-Michael addition followed by *syn*-mode ring closure determines the final product configuration.

Diastereoselectivity has also been systematically investigated using other arylideneoxindole derivatives and γ-phenyl/neopentyl allenoates.^34^ The choice of phosphine catalyst was found to significantly influence the stereochemical outcome; however, it had little effect on regioselectivity, with all catalysts consistently favoring formation of the α-regioisomer. Among the phosphines tested, only PPh_3_ delivered both high regio- and diastereoselectivity. In one representative example, the use of the less hindered phosphine PMe_2_Ph afforded three diastereoisomers in nearly equal amounts (∼1:1:1 ratio), with an overall yield of 80%. Based on the present mechanistic studies, this poor selectivity may be attributed to two factors. First, the reduced steric bulk of PMe_2_Ph alleviates steric repulsion with the γ-substituent during adduct formation, thereby diminishing torquoselectivity for the *trans*-configured adduct. Second, facial selectivity during ring closure becomes less pronounced, allowing *anti* cyclization to compete more effectively with the *syn* pathway. This decrease in stereocontrol may be related to the strength of noncovalent CH···O interactions, which have been identified in our investigations to play a significant role in guiding facial selectivity. Studies of C­(sp^2^)–H···O and C­(sp^3^)–H···O interactions have shown that the former exhibits shorter hydrogen bond distances and greater interaction energies, consistent with the greater polarization of the sp^2^-hybridized C–H bond (relevant to proton acidity).^65–67^ This trend suggests that the methyl groups of PMe_2_Ph participate in weaker noncovalent interactions during ring closure, thereby increasing the likelihood of *anti* cycloaddition.

### 2.3 Kinetic Regimes Modulated by Substituent Effects

Depending on the relative barrier heights for adduct isomerization and Michael addition (modulated by the substituent effects), the kinetic profile can be classified into three distinct regimes, illustrated in Figure 10. As phenyl substituents are introduced at the β-position of the enone and the γ-position of the allenoate, the kinetic scenario gradually shifts toward Curtin–Hammett control.

**Figure 10:**
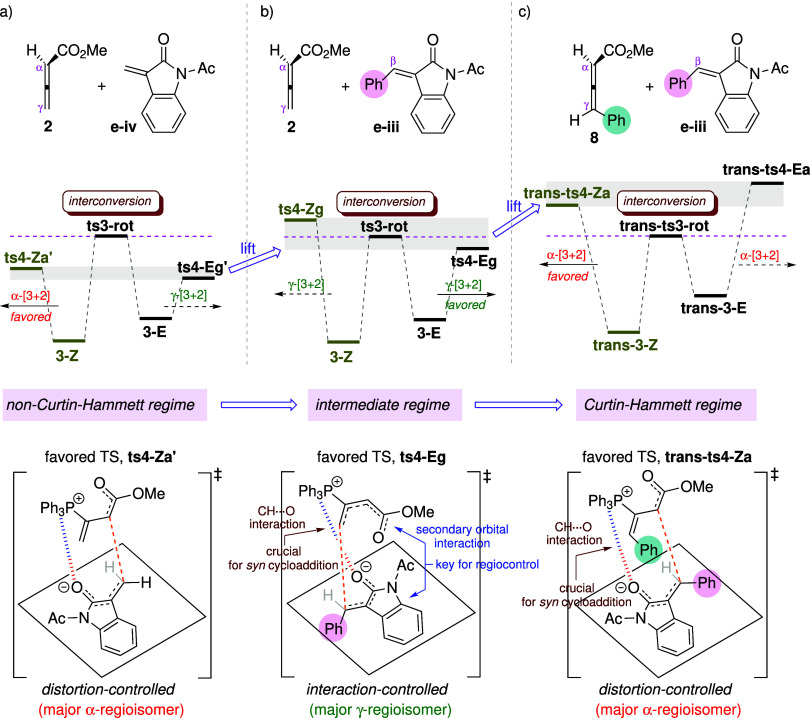
Three distinct kinetic regimes modulated by substituent effects of substrates. The regiodetermining TSs are shown at the bottom.

#### 2.3.1 Non-Curtin–Hammett Regime

In the cyclization of unsubstituted allenoate **2** and enone **e-iv** (Figure 10a), the kinetics operates under non-Curtin–Hammett control where the barrier for adduct interconversion is higher than those for Michael addition. Although the TSs of the subsequent reactions derived from **3-E** are energetically lower than those from **3-Z**, the pathway involving **3-E** is inaccessible due to kinetic quenching. As a result, regioselectivity is determined by the cycloaddition between the *Z*-adduct and the enone, affording the major α-regioisomer. The reaction mode of the regiodetermining TS, shown at the bottom of Figure 10a, is similar to that reported in previous computational studies employing unsubstituted substrates such as methyl allenoates and methyl acrylates.^40,43^

It is noteworthy that cyclization via the *Z*- and *E*-adducts leads to opposite regioselectivity in this system. Thus, if the barriers for Michael addition increase to approach or exceed that for adduct interconversion, **3-E** would begin to compete with **3-Z** in reacting with the enone, potentially reversing product regiochemistry. To assess how the relative barrier heights influence the regiochemical outcome, the product distribution is recomputed by simultaneously raising the activation energies of the four Michael addition TSs (**ts4-Za**′, **ts4-Zg**′, **ts4-Ea**′, and **ts4-Eg**′) in 1 kcal mol^–1^ increments (δ*G*) while maintaining their original relative differences. In other words, the kinetic simulations are performed using the adjusted TS energies of 14.5+δ*G*, 15.9+δ*G*, 13.8+δ*G*, and 13.6+δ*G* kcal mol^–1^ (Figure 6b), respectively, with the energy levels of all other species held unchanged. Figure 11 shows the variation in product distribution as a function of δ*G*, where hatched bars represent product ratios derived from **3-Z** and solid bars indicate those from **3-E**. The energy profile at δ*G* = 0.0 kcal mol^–1^ corresponds to the unperturbed case depicted in Figure 6b, with an α:γ ratio of 91:9. As δ*G* increases, the proportion of the γ-regioisomer rises, reflecting a growing contribution from the cyclization of the *E*-adduct. When δ*G* ≥ 5 kcal mol^–1^, the activation barriers for all four Michael addition modes exceed that for adduct interconversion (Δ*G* ^‡^: 18.3 kcal mol^–1^), marking a transition to Curtin–Hammett control. Under this regime, the computed product ratio converges to 12:1:34:53 (in line with the statistical distribution estimated from the equation shown in Figure 1a), resulting in an α:γ ratio of 46:54. The modeling test confirms that the relative barrier heights between adduct isomerization and Michael addition critically influence regioselectivity, particularly when the *Z*- and *E*-adducts lead to opposite regiochemical outcomes. The change in α:γ selectivity from 91:9 to 46:54 is not caused by the energetics of the regioselective step itself, but rather by the degree of favorability of adduct interconversion. As the kinetic regime transitions from non-Curtin–Hammett to Curtin–Hammett control, the factors influencing regioselectivity become more complex. The results appear to offer a rationale for the variability in regioselectivity typically observed when allenoates/allenic ketones and enones lack substituents at the γ- and β-positions, respectively.^14,21,24^ One strategy to minimize kinetic complexities related to adduct equilibration as much as possible is to employ substituted (or less reactive) enones by lifting the activation barriers to cyclization.

**Figure 11:**
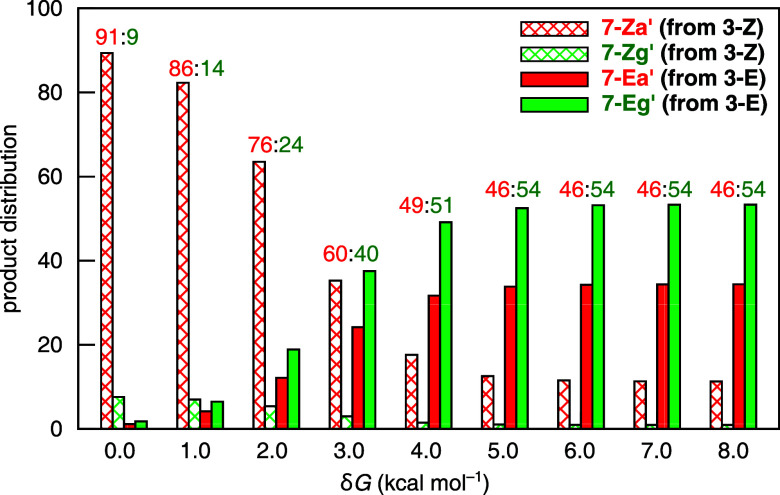
Variation of product distribution with δ*G*. The computed α:γ ratios, defined as ([**7-Za**′]+[**7-Ea**′]):([**7-Zg**′]+[**7-Eg**′]), are shown above the bars.

#### 2.3.2 Intermediate Regime

When a phenyl substituent is present at the β-position of the enone (Figure 10b), the activation barriers for the four Michael addition modes are consistently raised. Consequently, the kinetic profile falls within the intermediate regime between non-Curtin–Hammett and Curtin–Hammett control: among the four TSs examined, only **ts4-Eg** exhibits a barrier lower than the TS for adduct isomerization (**ts3-rot**), while the others lie above it. Kinetic simulations demonstrate that the [3 + 2] cycloaddition preferentially proceeds via **ts4-Eg** (derived from the less stable *E*-adduct), yielding the γ-regioisomer as the major product. The observed γ-regioselectivity is attributed to favorable secondary orbital interactions between the ester group and the oxindole ring.

#### 2.3.3 Curtin–Hammett Regime

When substituted allenoate **8** and enone **e-iii** are employed as the substrates (Figure 10c), the activation energies for all four Michael addition modes are higher than that for adduct interconversion. Therefore, the kinetics of regioselection follows the Curtin–Hammett principle, where product selectivity is determined by the relative activation energies of the four competing TSs in the Michael addition step (irrespective of the energetics of adduct interconversion). Under this condition, the ratio of the isomeric products can be estimated using the equation shown in Figure 1a. The distortion/interaction analysis reveals that the preference for the α-[3 + 2] annulation is distortion-controlled, as observed for the α-selectivity in Figure 10a. In addition, the presence of noncovalent CH···O interactions is a crucial factor that biases the reaction toward *syn*-mode cyclization.

## 3 Conclusions

The substituent effects of the substrates in the PPh_3_-catalyzed [3 + 2] cycloaddition are elucidated through DFT calculations and kinetic simulations. Nucleophilic addition of the phosphine catalyst to the allenoate preferentially generates a twisted adduct, which subsequently interconverts with the *Z*- and *E*-isomeric adducts. Among the three isomeric adducts, the least stable twisted form is involved solely in the initial addition and interconversion processes, whereas the *Z*- and *E*-isomers participate in subsequent cyclization. Regioselectivity in the cycloaddition reaction is determined not only by the Michael addition step, but also by the equilibration between the *Z*- and *E*-adducts. As substituents are attached to the β-position of the enone and the γ-position of the allenoate, the barrier heights for the Michael addition are elevated due to increased steric hindrance (Figure 10). Consequently, the kinetic regime shifts toward Curtin–Hammett control.

Electronic and steric factors significantly affect the relative energy differences among the four Michael addition modes. In the reaction of **2** with **e-iii**, a secondary orbital interaction preferentially stabilizes the γ-mode of Michael addition proceeding via the *E*-adduct. In contrast, introducing a γ-substituent on the allenoate destabilizes the pathways involving the *E*-adduct (due to increased steric strain). As a result, the α-mode addition via the *Z*-adduct becomes favored, as observed in the cyclization of **8** with **e-iii**. The torquoselective (*trans* over *cis*), regioselective (α over γ), and stereoselective (*syn* over *anti*) processes jointly direct the formation of the α-regioisomer bearing a *trans*-arrangement of the two phenyl substituents relative to the oxindole carbonyl. These key selective steps enable the efficient creation of three stereogenic centers in the final product. The insights into the origins of regio- and stereochemical control can assist in the rational design of catalysts and the judicious variation of substrates for highly selective transformations.

Our computations demonstrate how the favorability of adduct interconversion influences the accessibility of subsequent reaction pathways, and how the substituent effects of the substrates modulate regioselectivity (by shifting the kinetic regime). The mechanistic framework for adduct formation/interconversion is applicable not only to the [3 + 2] cycloaddition with enone substrates, but also potentially to a broader range of phosphine-catalyzed transformation involving other substrates. The following three factors are expected to influence product selectivity: (1) substrate concentration and reactivity that determine the rate of subsequent reactions, *v* = *k*[adduct]­[substrate]; (2) entropic effects associated with intra- versus intermolecular processes (e.g., adduct interconversion vs subsequent reactions); (3) the steric bulk of the phosphine catalyst^46^ relevant to the *Z*/*E*-isomerism of the adducts. Overall, this work provides mechanistic insight into phosphine·allenoate chemistry and underscores the central role of adduct interconversion dynamics in catalysis. The kinetic interplay between adduct interconversion and subsequent reactions ultimately governs the accessibility of downstream pathways and the resulting product distribution.

## 4 Computational Details

The computational workflow for constructing the free energy profile of the catalytic cycle is outlined in Figure S1. The same protocol has been successfully employed in other studies of catalytic systems.^68^ The conformer-rotamer ensemble sampling tool (CREST, version 2.11)^69,70^ was first employed to explore conformational space, using the tight-binding semiempirical method GFN2-xTB.^71^ Following conformer sampling, single-point energy calculations were performed at the ωB97X-D^72^/6-31G­(d)^73^ level of theory. The solvent effect of toluene, as employed in experimental studies (Scheme 3), was taken into account through the SMD solvation model.^74^ Based on these single-point energies, the ten lowest-energy conformers were selected for further geometry optimization at the ωB97X-D/6-311G­(d,p) level. Frequency calculations were conducted to confirm the nature of the optimized structures: the TS was characterized by only one imaginary frequency corresponding to the desired reaction coordinate, and the minimum by real frequencies for all vibrational modes. The details of barrier calculations (with respect to internal rotation) were provided in the ESI. Entropic contributions from low-frequency vibrational modes^75^ were treated using the quasi-rigid-rotor harmonic oscillator (quasi-RRHO) approximation.^76^ Thermodynamic properties were computed at 298.15 K (25 °C), and thermal corrections were included to compute thermodynamic quantities such as free energies. For further refinement, single-point electronic energies were calculated using the larger def2-TZVP basis set.^77^ Tests of the Ahlrichs-type triple-ζ basis set showed that the computed energetics were not significantly changed by the inclusion of diffuse functions (Table S2). An ultrafine integration grid (pruned 99,590) was used throughout all DFT calculations. Additionally, a free energy correction of 1.9 kcal mol^–1^ was included to account for the standard-state concentration change from 1 atm (gas phase) to 1 M (solution phase). The final energy profile was constructed from Boltzmann-weighted free energies based on the five most stable conformers for each state. For certain species with fewer than five conformers (e.g., catalyst **1**), all available conformers were used directly. The lowest-energy conformers were employed as representative geometries for detailed structural analysis in the main text. All DFT calculations were performed using the Gaussian 09 suite of programs.^78^ Furthermore, single-point energy calculations were performed using other methods to validate the DFT results (Tables S3 and S4, and Figure S2 in the ESI). The consistent trends observed across different levels of theory support the reliability of the calculated energy profiles.

An allenoate can adopt two conformations of *s-cis* and *s-trans*, which have been observed by NMR spectroscopy to interconvert rapidly.^79^ To account for this conformational equilibrium, both forms were included in the calculation of Boltzmann-weighted free energies for the reactant state. Preliminary calculations have shown that the barriers for the attack of PPh_3_ on the *s-cis* allenoate are lower than those on the *s-trans* isomer (Figure S3), in agreement with previous computational studies.^42,54,80^ As the initial addition step has been identified as rate-determining,^43–45^ the intermediates arising from the lower-barrier pathway are expected to exist as dominant species during catalysis. Accordingly, all mechanistic investigations were conducted along the energetically favored pathway derived from the *s-cis* conformer. IRC^62^ and relaxed scan calculations were performed to confirm that the TSs associated with initial addition and adduct isomerization were linked to the desired reactants and products (Figures S4 and S5). The bonding patterns of the adducts were analyzed using electron localization function (ELF).^61^ The comparison of the calculated electron populations has shown that the bonding pattern of the allenic moiety in the *Z*/*E*-adducts resembles that of *s-trans*/*s-cis* dienes more closely than that of an allyl anion (Figure S6). Therefore, the negatively charged group in the zwitterionic adduct is primarily described as an enolate throughout the main context. In addition, the noncovalent interaction (NCI) analysis^81^ was employed to identify CH···O nonclassical hydrogen bonds^65^ present in key TS structures (Figures S8 to S10).^82^

## Supplementary Material





## Data Availability

The data underlying this study are available in the published article and its Supporting Information.

## Notes

Comparison of addition pathways, computational workflow, conformational search, effects of diffuse functions, tests of other functionals, IRC and relaxed scan calculations, identification of noncovalent interactions, differential equations used in the kinetic modeling of the reaction network, and Cartesian coordinates and energies of all optimized minima and TSs (PDF)
Cartesian coordinates of optimized structures (ZIP)

## References

[1] Guo H., Fan Y. C., Sun Z., Wu Y., Kwon O. (2018). Phosphine
Organocatalysis. Chem. Rev..

[2] Xie C., Smaligo A. J., Song X. R., Kwon O. (2021). Phosphorus-Based
Catalysis.
ACS Cent. Sci..

[3] Gorenstein D., Westheimer F. H. (1970). Nuclear magnetic resonance evidence for the pathways
of pseudorotation in alkyloxyphosphoranes. J.
Am. Chem. Soc..

[4] Buono G., Llinas J. R. (1981). Oxyphosphoranes with an oxaphospholene ring: analysis
of the activation barriers of the isomerization process. J. Am. Chem. Soc..

[5] Wu X. Y., Gui H. Z., Jangra H., Wei Y., Zipse H., Shi M. (2020). Phosphine-catalyzed [3 + 2] annulation of 2-aminoacrylates with allenoates
and mechanistic studies. Catal. Sci. Technol..

[6] An F., Jangra H., Wei Y., Shi M., Zipse H., Ofial A. R. (2022). Reactivities of allenic and olefinic Michael acceptors
towards phosphines. Chem. Commun..

[7] An F., Brossette J., Jangra H., Wei Y., Shi M., Zipse H., Ofial A. R. (2024). Reactivities of tertiary phosphines
towards allenic, acetylenic, and vinylic Michael acceptors. Chem. Sci..

[8] Cowen B. J., Miller S. J. (2009). Enantioselective catalysis and complexity generation
from allenoates. Chem. Soc. Rev..

[9] Wang Z., Xu X., Kwon O. (2014). Phosphine
catalysis of allenes with electrophiles. Chem.
Soc. Rev..

[10] Wei Y., Shi M. (2014). Applications of chiral
phosphine-based organocatalysts in catalytic
asymmetric reactions. Chem.–Asian J..

[11] Zhou R., He Z. (2016). Advances in Annulation Reactions
Initiated by Phosphorus Ylides Generated
in situ. Eur. J. Org. Chem..

[12] Li H., Lu Y. (2017). Enantioselective Construction
of All-Carbon Quaternary Stereogenic
Centers by Using Phosphine Catalysis. Asian
J. Org. Chem..

[13] Gomez C., Betzer J. F., Voituriez A., Marinetti A. (2013). Phosphine
Organocatalysis in the Synthesis of Natural Products and Bioactive
Compounds. ChemCatChem..

[14] Zhang C., Lu X. (1995). Phosphine-Catalyzed
Cycloaddition of 2,3-Butadienoates or 2-Butynoates
with Electron-Deficient Olefins. A Novel [3 + 2] Annulation Approach
to Cyclopentenes. J. Org. Chem..

[15] Lu X., Zhang C., Xu Z. (2001). Reactions
of electron-deficient alkynes
and allenes under phosphine catalysis. Acc.
Chem. Res..

[16] Wei Y., Shi M. (2017). Lu’s [3 + 2]
cycloaddition of allenes with electrophiles:
Discovery, development and synthetic application. Org. Chem. Front..

[17] Ung A. T., Schafer K., Lindsay K. B., Pyne S. G., Amornraksa K., Wouters R., Van der Linden I., Biesmans I., Lesage A. S., Skelton B. W., White A. H. (2002). Synthesis and biological activities
of conformationally restricted cyclopentenyl-glutamate analogues. J. Org. Chem..

[18] Du Y., Lu X. (2003). A phosphine-catalyzed [3 + 2] cycloaddition strategy leading to the
first total synthesis of (−)-hinesol. J. Org. Chem..

[19] Pham T. Q., Pyne S. G., Skelton B. W., White A. H. (2005). Synthesis of carbocyclic
hydantocidins via regioselective and diastereoselective phosphine-catalyzed
[3 + 2]-cycloadditions to 5-methylenehydantoins. J. Org. Chem..

[20] Voituriez A., Marinetti A., Gicquel M. (2014). Phosphine Organocatalysis for the
Synthesis of Spirocyclic Compounds. Synlett.

[21] Du Y., Lu X., Yu Y. (2002). Highly regioselective construction
of spirocycles via
phosphine-catalyzed [3 + 2]-cycloaddition. J.
Org. Chem..

[22] Wilson J.
E., Fu G. C. (2006). Synthesis
of functionalized cyclopentenes through catalytic asymmetric
[3 + 2] cycloadditions of allenes with enones. Angew. Chem., Int. Ed..

[23] Voituriez A., Panossian A., Fleury-Brégeot N., Retailleau P., Marinetti A. (2008). 2-Phospha­[3]­ferrocenophanes with planar chirality:
Synthesis and use in enantioselective organocatalytic [3 + 2] cyclizations. J. Am. Chem. Soc..

[24] Wallace D. J., Sidda R. L., Reamer R.a. (2007). Phosphine-Catalyzed Cycloadditions
of Allenic Ketones: New Substrates for Nucleophilic Catalysis. J. Org. Chem..

[25] Zou Y. Q., Li C., Rong J., Yan H., Chen J. R., Xiao W. J. (2011). Phosphine-catalyzed
[3 + 2] cycloadditions of 2-phenyl-4-arylidene-5­(4 H)-oxazolones with
allenoate: A concise synthesis of aspartic acid analogues. Synlett.

[26] Pinto N., Neel M., Panossian A., Retailleau P., Frison G., Voituriez A., Marinetti A. (2010). Expanding
the Scope of Enantioselective FerroPHANE-Promoted [3 + 2] Annulations
with α,β-Unsaturated Ketones. Chem.–Eur.
J..

[27] Voituriez A., Panossian A., Fleury-Brégeot N., Retailleau P., Marinetti A. (2009). Synthesis of chiral 2-phospha[3]­ferrocenophanes and
their behaviour as organocatalysts in [3 + 2]­Cyclization reactions. Adv. Synth. Catal..

[28] Duvvuru D., Pinto N., Gomez C., Betzer J.-F., Retailleau P., Voituriez A., Marinetti A. (2012). Heterocyclic Spiranes and Dispiranes
via Enantioselective Phosphine-Catalyzed [3 + 2] Annulations. Adv. Synth. Catal..

[29] Yang L. J., Cai H., Nie J., Ma J. A. (2013). A highly regio-and diastereoselective
phosphane-catalyzed [3 + 2] annulation of morita-baylis-hillman carbonates
with cyclic N-acyl ketimines. Eur. J. Org. Chem..

[30] Kitagaki S., Nakayoshi T., Masunaka S., Uchida A., Inano M., Yoshida E., Washino Y., Aoyama H., Yoshida K. (2024). Highly regio-
and stereoselective (3 + 2) annulation reaction of allenoates with
3-methyleneindolin-2-ones catalyzed by a planar chiral [2.2]­paracyclophane-based
bifunctional phosphine–phenol catalyst. Org. Biomol. Chem..

[31] Li X., Wang F., Dong N., Cheng J.-P. (2013). Phosphine-containing
Lewis base catalyzed cyclization of benzofuranone type electron-deficient
alkenes with allenoates: a facile synthesis of spirocyclic benzofuranones. Org. Biomol. Chem..

[32] Gao Z., Wang C., Yuan C., Zhou L., Sun Z., Xiao Y., Guo H. (2015). Phosphine-catalyzed asymmetric [3
+ 2] annulation of chalcones with allenoates for enantioselective
synthesis of functionalized cyclopentenes. RSC
Adv..

[33] Voituriez A., Pinto N., Neel M., Retailleau P., Marinetti A. (2010). An Organocatalytic
[3 + 2] Cyclisation Strategy for
the Highly Enantioselective Synthesis of Spirooxindoles. Chem.–Eur. J..

[34] Gomez C., Gicquel M., Carry J. C., Schio L., Retailleau P., Voituriez A., Marinetti A. (2013). Phosphine-catalyzed synthesis of
3,3-spirocyclopenteneoxindoles from γ-substituted allenoates:
Systematic studies and targeted applications. J. Org. Chem..

[35] Wang Y., Zheng L., Wei D., Tang M. (2015). A quantum mechanical
study of the mechanism and stereoselectivity of the N-heterocyclic
carbene catalyzed [4 + 2] annulation reaction of enals with azodicarboxylates. Org. Chem. Front..

[36] Anwar S., Lin L.-T., Srinivasadesikan V., Gudise V. B., Chen K. (2021). [3 + 2] regioselective
annulation reaction of 2-arylidene-1,3-indandiones towards synthesis
of spirocyclopentenes: understanding the mechanism of γ-attack
vs. α-attack using DFT studies. RSC Adv..

[37] Cowen B. J., Miller S. J. (2007). Enantioselective
[3 + 2]-cycloadditions catalyzed by
a protected, multifunctional phosphine-containing α-amino acid. J. Am. Chem. Soc..

[38] Zhou W., Wang H., Tao M., Zhu C. Z., Lin T. Y., Zhang J. (2017). Phosphine-catalyzed enantioselective [3 + 2] cycloadditions of γ-substituted
allenoates with β-perfluoroalkyl enones. Chem. Sci..

[39] Liu R., Qin Z., Fan B., Li R., Zhou R., He Z. (2019). Phosphine-Catalyzed
Chemo- And Diastereoselective [2 + 2 + 2] and [3 + 2] Annulations
of γ-Methyl Allenoates with Doubly Activated Olefins: Syntheses
of Highly Substituted Cyclohexanes and Cyclopentenes. J. Org. Chem..

[40] Dudding T., Kwon O., Mercier E. (2006). Theoretical Rationale for Regioselection
in Phosphine-Catalyzed Allenoate Additions to Acrylates, Imines, and
Aldehydes. Org. Lett..

[41] Xia Y., Liang Y., Chen Y., Wang M., Jiao L., Huang F., Liu S., Li Y., Yu Z.-X. (2007). An Unexpected
Role of a Trace Amount of Water in Catalyzing Proton Transfer in Phosphine-Catalyzed
(3 + 2) Cycloaddition of Allenoates and Alkenes. J. Am. Chem. Soc..

[42] Mercier E., Fonovic B., Henry C., Kwon O., Dudding T. (2007). Phosphine
triggered [3 + 2] allenoate-acrylate annulation: a mechanistic enlightenment. Tetrahedron Lett..

[43] Liang Y., Liu S., Xia Y., Li Y., Yu Z. X. (2008). Mechanism, regioselectivity,
and the kinetics of phosphine-catalyzed [3 + 2] cycloaddition reactions
of allenoates and electron-deficient alkenes. Chem.–Eur. J..

[44] Fujiwara Y., Fu G. C. (2011). Application of a New Chiral Phosphepine to the Catalytic Asymmetric
Synthesis of Highly Functionalized Cyclopentenes That Bear an Array
of Heteroatom-Substituted Quaternary Stereocenters. J. Am. Chem. Soc..

[45] Lee S. Y., Fujiwara Y., Nishiguchi A., Kalek M., Fu G. C. (2015). Phosphine-Catalyzed
Enantioselective Intramolecular [3 + 2] Annulations to Generate Fused
Ring Systems. J. Am. Chem. Soc..

[46] Zhu X. F., Schaffner A. P., Li R. C., Kwon O. (2005). Phosphine-catalyzed
synthesis of 6-substituted 2-pyrones: Manifestation of E/Z-isomerism
in the zwitterionic intermediate. Org. Lett..

[47] Zhu X. F., Henry C. E., Wang J., Dudding T., Kwon O. (2005). Phosphine-catalyzed
synthesis of 1,3-dioxan-4-ylidenes. Org. Lett..

[48] Pollak P. I., Curtin D. Y. (1950). Stereospecificity
in the Rearrangement of Amino Alcohols. J. Am.
Chem. Soc..

[49] Curtin D. Y., Pollak P. I. (1951). Stereospecificity in the Rearrangement of Aminoalcohols.
II 1. J. Am. Chem. Soc..

[50] Seeman J. I., Farone W. A. (1978). Analytical Solution
to the Curtin-Hammett/Winstein-Holness
Kinetic System1. J. Org. Chem..

[51] Seeman J. I. (1983). Effect
of conformational change on reactivity in organic chemistry. Evaluations,
applications, and extensions of Curtin-Hammett Winstein-Holness kinetics. Chem. Rev..

[52] Seeman J. I. (1986). The Curtin-Hammett
principle and the Winstein-Holness equation: new definition and recent
extensions to classical concepts. J. Chem. Educ..

[53] Chakraborty S., Saha C. (2016). The Curtin-Hammett
principle. Resonance.

[54] Huang G.-T., Lankau T., Yu C.-H. (2014). A Computational
Study: Reactivity
Difference between Phosphine- and Amine-Catalyzed Cycloadditions of
Allenoates and Enones. J. Org. Chem..

[55] Bickelhaupt F. M., Houk K. N. (2017). Analyzing Reaction
Rates with the Distortion/Interaction-Activation
Strain Model. Angew. Chem., Int. Ed..

[56] Zhang C., Lu X. (1995). Umpolung Addition Reaction
of Nucleophiles to 2,3-Butadienoates Catalyzed
by a Phosphine. Synlett.

[57] Xu Z., Lu X. (1997). Phosphine-catalyzed
[3 + 2] cycloaddition reaction of methyl 2,3-butadienoate
and N-tosylimines. A novel approach to nitrogen heterocycles. Tetrahedron Lett..

[58] Xu Z., Lu X. (1998). A Novel [3 + 2] Cycloaddition Approach to Nitrogen
Heterocycles via
Phosphine-Catalyzed Reactions of 2,3-Butadienoates or 2-Butynoates
and Dimethyl Acetylenedicarboxylate with Imines: A Convenient Synthesis
of Pentabromopseudilin. J. Org. Chem..

[59] Duan M., Zhu L., Qi X., Yu Z., Li Y., Bai R., Lan Y. (2017). From Mechanistic Study
to Chiral Catalyst Optimization: Theoretical
Insight into Binaphthophosphepine-catalyzed Asymmetric Intramolecular
[3 + 2] Cycloaddition. Sci. Rep..

[60] Huang G. T., Lankau T., Yu C. H. (2014). A computational
study of the activation
of allenoates by Lewis bases and the reactivity of intermediate adducts. Org. Biomol. Chem..

[61] Becke A. D., Edgecombe K. E. (1990). A simple measure of electron localization in atomic
and molecular systems. J. Chem. Phys..

[62] Fukui K. (1981). The path of
chemical reactions-the IRC approach. Acc. Chem.
Res..

[63] Ni H., Yu Z., Yao W., Lan Y., Ullah N., Lu Y. (2017). Catalyst-controlled
regioselectivity in phosphine catalysis: The synthesis of spirocyclic
benzofuranones via regiodivergent [3 + 2] annulations of aurones and
an allenoate. Chem. Sci..

[64] Gallardo-Fuentes S., Ormazábal-Toledo R., Fernández I. (2020). Unraveling
the Selectivity Patterns in Phosphine-Catalyzed Annulations of Azomethine
Imines and Allenoates. J. Org. Chem..

[65] Johnston R. C., Cheong P. H. Y. (2013). CH···O
non-classical hydrogen bonding
in the stereomechanics of organic transformations: Theory and recognition. Org. Biomol. Chem..

[66] Gu Y., Kar T., Scheiner S. (1999). Fundamental properties of the CH ···
O interaction: Is it a true hydrogen bond?. J. Am. Chem. Soc..

[67] Scheiner S., Kar T. (2002). Red- versus blue-shifting hydrogen bonds: Are there fundamental distinctions?. J. Phys. Chem. A.

[68] Huang G. T., Yu J. S. K. (2024). Catalytic role of the enol ether intermediate in the
intramolecular Stetter reaction: a computational perspective. Phys. Chem. Chem. Phys..

[69] Pracht P., Bohle F., Grimme S. (2020). Automated exploration of the low-energy
chemical space with fast quantum chemical methods. Phys. Chem. Chem. Phys..

[70] Pracht P., Grimme S., Bannwarth C., Bohle F., Ehlert S., Feldmann G., Gorges J., Müller M., Neudecker T., Plett C. (2024). CREST—A program
for the exploration of low-energy molecular chemical space. J. Chem. Phys..

[71] Bannwarth C., Ehlert S., Grimme S. (2019). GFN2-xTB—An
accurate and broadly
parametrized self-consistent tight-binding quantum chemical method
with multipole electrostatics and density-dependent dispersion contributions. J. Chem. Theory Comput..

[72] Chai J.-D., Head-Gordon M. (2008). Long-range corrected hybrid density
functionals with
damped atom–atom dispersion corrections. Phys. Chem. Chem. Phys..

[73] Hariharan P. C., Pople J. A. (1973). The influence of polarization functions on molecular
orbital hydrogenation energies. Theor. Chim.
Acta..

[74] Marenich A.
V., Cramer C. J., Truhlar D. G. (2009). Universal solvation model based on
solute electron density and on a continuum model of the solvent defined
by the bulk dielectric constant and atomic surface tensions. J. Phys. Chem. B.

[75] Grimme S. (2012). Supramolecular
binding thermodynamics by dispersion-corrected density functional
theory. Chem.–Eur. J..

[76] Lu T., Chen Q. (2021). Shermo: A general code for calculating molecular thermochemistry
properties. Comput. Theor. Chem..

[77] Weigend F., Ahlrichs R. (2005). Balanced basis sets
of split valence, triple zeta valence
and quadruple zeta valence quality for H to Rn: Design and assessment
of accuracy. Phys. Chem. Chem. Phys..

[78] Frisch, M. J. ; Trucks, G. W. ; Schlegel, H. B. ; Scuseria, G. E. ; Robb, M. A. ; Cheeseman, J. R. ; Scalmani, G. ; Barone, V. ; Mennucci, B. ; Petersson, G. A. ; Nakatsuji, H. ; Caricato, M. ; Li, X. ; Hratchian, H. P. ; Izmaylov, A. F. ; Bloino, J. ; Zheng, G. ; Sonnenberg, J. L. ; Hada, M. ; Ehara, M. ; Toyota, K. ; Fukuda, R. ; Hasegawa, J. ; Ishida, M. ; Nakajima, T. ; Honda, Y. ; Kitao, O. ; Nakai, H. ; Vreven, T. ; Montgomery, J. A., Jr ; Peralta, J. E. ; Ogliaro, F. ; Bearpark, M. ; Heyd, J. J. ; Brothers, E. ; Kudin, K. N. ; Staroverov, V. N. ; Kobayashi, R. ; Normand, J. ; Raghavachari, K. ; Rendell, A. ; Burant, J. C. ; Iyengar, S. S. ; Tomasi, J. ; Cossi, M. ; Rega, N. ; Millam, J. M. ; Klene, M. ; Knox, J. E. ; Cross, J. B. ; Bakken, V. ; Adamo, C. ; Jaramillo, J. ; Gomperts, R. ; Stratmann, R. E. ; Yazyev, O. ; Austin, A. J. ; Cammi, R. ; Pomelli, C. ; Ochterski, J. W. ; Martin, R. L. ; Morokuma, K. ; Zakrzewski, V. G. ; Voth, G. A. ; Salvador, P. ; Dannenberg, J. J. ; Dapprich, S. ; Daniels, A. D. ; Farkas, O. ; Foresman, J. B. ; Ortiz, J. V. ; Cioslowski, J. ; Fox, D. J. Gaussian 09 Revision E.01; Gaussian Inc: Wallingford CT, 2009.

[79] Gandhi R. P., Ishar M. P. (1991). Carbon-13 NMR spectral investigations on allenic esters:
Rationalization of carbon-13 NMR chemical shifts and relaxation times
(T1). Magn. Reson. Chem..

[80] Holland M. C., Gilmour R., Houk K. N. (2016). Importance
of intermolecular hydrogen
bonding for the stereochemical control of allene-enone (3 + 2) annulations
catalyzed by a bifunctional, amino acid derived phosphine catalyst. Angew. Chem., Int. Ed..

[81] Johnson E. R., Keinan S., Mori-Sánchez P., Contreras-García J., Cohen A. J., Yang W. (2010). Revealing noncovalent interactions. J. Am. Chem. Soc..

[82] Lu T., Chen F. (2012). Multiwfn: a multifunctional wavefunction analyzer. J. Comput. Chem..

