# Supplementary material for: Origin of Substituent-Modulated Regioselectivity in Phosphine-Catalyzed [3 + 2] Cyclization of Allenoates and Enones: A Kinetic Shift toward Curtin–Hammett Control
Source: J Org Chem. 2025 Oct 8;90(41):14541–56. doi: 10.1021/acs.joc.5c01466 (PMC12538593; doi:10.1021/acs.joc.5c01466)
Supplement: Supplementary file 1 [file jo5c01466_si_001.pdf]

Supporting Information

Origin of Substituent-Modulated Regioselectivity in Phosphine-Catalyzed [3+2] Cyclization of Allenoates and Enones: A Kinetic Shift toward Curtin-Hammett Control

Gou-Tao Huang*,† and Jen-Shiang K. Yu*,†,‡,¶

†*Department of Biological Science and Technology, National Yang Ming Chiao Tung University, Hsinchu City 300, Taiwan*

‡*Institute of Bioinformatics and Systems Biology, National Yang Ming Chiao Tung University, Hsinchu City 300, Taiwan*

¶*Center for Intelligent Drug Systems and Smart Bio-devices (IDS^{2}B), National Yang Ming Chiao Tung University, Hsinchu City 300, Taiwan*

E-mail: gthtown@gmail.com; jsyu@mail.nctu.edu.tw

Phone: +886 (3)5729287. Fax: +886 (3)5729288

Contents

List of Figures **S4**

List of Tables **S7**

1 Comparison of PPh_3 and PMe_3 **S8**

2 Computational workflow **S9**

2.1 Conformational search by CREST S10

3 Assessment of basis sets and functionals **S12**

3.1 Effects of diffuse functions S12

3.2 Tests of other functionals S14

3.3 DLPNO-CCSD(T) calculations S15

4 Adduct formation **S17**

4.1 Isomeric TSs for initial addition S17

4.2 Rotational barrier and IRC calculations S18

4.3 Comparison of bonding and geometric properties S21

4.4 Identification of key interactions by NCI analysis S23

4.5 Frontier orbital interactions S25

4.6 Michael addition of **3-Z/3-E** to **e-iii** S26

4.7 Ring closure in *syn* vs *anti* modes S27

4.8 Michael addition of **3-Z/3-E** to **e-iv** S28

5 Reactions of γ-substituted allenoates **S29**

6 Kinetic simulations **S33**

7 Cartesian coordinates of optimized structures **S36**

List of Figures

S1 Computational workflow and approaches to data analysis. S9

S2 Free energy profiles ($\Delta G_{\text{DFT, sol}}$ and $\Delta G_{\text{CC, sol}}$ in kcal mol^{-1}) based on the most stable conformers. Values shown in red and blue correspond to free energies computed at the ωB97X-D and DLPNO-CCSD(T) levels, respectively. S16

S3 Isomeric TSs for nucleophilic addition of PPh_3 to *s-cis*/*s-trans* allenoate **2**. Computed activation free energies ($\Delta G^{\ddagger}$ in kcal mol^{-1}) are reported with respect to the separated reactants. S17

S4 IRC calculations of initial addition and adduct isomerization. The units of the x- and y-axes are intrinsic reaction coordinate (amu$^{1/2}$ bohr) and electronic energies (au), respectively. S19

S5 Scan calculations with respect to the specified dihedral angle. The units of the x- and y-axes are dihedral angle (°) and electronic energies (au), respectively. S20

S6 Calculated C−C bond lengths and electron populations of the corresponding valence basins (shown in bold red). S21

S7 Optimized structures of initial addition of PPh_3 to unsubstituted allenoate **2**. Selected structural parameters with respect to the allenic moiety are listed in magenta. The noncovalent interactions of CH···O are represented by green dashed lines. S22

S8 a) Electrostatic potential mapped on an electron density isosurface of 0.02 a.u., and b) NCI analysis of the lowest energy conformers for initial addition. The NCI surface corresponds to $s = 0.5$ au and the NCI color scale ranges from −0.04 to 0.02 au. S23

S9 NCI analysis of the lowest energy TS conformers in the [3+2] cyclization of **2** and **e-iii**. The NCI surface corresponds to $s = 0.5$ au and the NCI color scale ranges from −0.04 to 0.02 au. S24

S10 NCI analysis of the lowest energy TS conformers in the [3+2] cyclization of **8** and **e-iii**. The NCI surface corresponds to $s = 0.5$ au and the NCI color scale ranges from -0.04 to 0.02 au. S24

S11 Frontier molecular orbitals of a) adducts **3-Z/3-E**, b) enone **e-iii/e-iv**, and c) the favored TS **ts4-Eg**. To better visualize the secondary orbital interaction, the isosurface of its HOMO is displayed at a reduced value of 0.01. . . . . . S25

S12 Optimized structures of Michael addition of **3-Z/3-E** to **e-iii**. Key structural parameters of the lowest-energy conformers are shown in magenta, and noncovalent interactions are indicated by green dashed lines. Free energies and enthalpies (in kcal mol^{-1}) are reported relative to the separated reactants. All conformers for each mode are compared by aligning the four atoms of P, Cα, Cβ, and Cγ, with the most stable conformers highlighted in orange. S26

S13 TS structures associated with facial selectivity in the ring-closure step. In the lowest-energy conformer, key structural parameters are shown in magenta, and noncovalent interactions are indicated by green dashed lines. The electrostatic potential (units in a.u.) mapped onto an electron density isosurface of 10^{-4} a.u. S27

S14 a) Four modes of Michael addition of **3-Z/3-E** to **e-iv**. b) Analysis of the distortion-interaction model based on electronic energies (kcal mol^{-1}). Optimized TS structures are shown at the bottom, with the most stable conformers highlighted in orange. Free energies and enthalpies (in kcal mol^{-1}) are reported relative to the separated reactants. S28

S15 Optimized structures of initial addition of PPh_3 to γ-substituted allenoates in terms of the *trans*-configuration. Selected structural parameters with respect to the allenic moiety are listed in magenta. The noncovalent interactions of CH···O are represented by green dashed lines. S30

S16 Optimized structures of initial addition of PPh_3 to γ-substituted allenoates in terms of the *cis*-configuration. Selected structural parameters with respect to the allenic moiety are listed in magenta. The noncovalent interactions of CH···O are represented by green dashed lines. S31
S17 TS structures with respect to a) Michael addition and b) ring closure. Selected structural parameters with respect to the allenic moiety are listed in magenta. The noncovalent interactions of CH···O are represented by green dashed lines. S32
S18 Variation of the concentrations in the kinetic modeling. S34

List of Tables

S1 Number of the conformers and the constrained parameters for the TSs. . . . S11

S2 Effects of diffuse functions on energetics for initial addition. The reported electronic energies (in kcal mol^{-1}) are given relative to the energy of the separated reactants (**1** + **2** + **e-iii**). S13

S3 Relative electronic energies ($\Delta\Delta E_{\mathrm{ele}}$ in kcal mol^{-1}) of the three isomeric TSs, **ts3-Z**, **ts3-E**, and **ts3-int**. The single-point calculations are performed using the def2-TZVP basis set. The reported electronic energies are given relative to the energy of **ts3-int**. S14

S4 Relative electronic energies ($\Delta\Delta E_{\mathrm{ele}}$ in kcal mol^{-1}) of the four regioisomeric TSs, **ts4-Za**, **ts4-Zg**, **ts4-Ea**, and **ts4-Eg**. The single-point calculations are performed using the def2-TZVP basis set. The reported electronic energies are given relative to the energy of **ts4-Eg**. S14

S5 Initial concentrations and results of kinetic simulations. S35

1 Comparison of PPh_3 and PMe_3

Scheme S1a shows three pathways for nucleophilic addition of PPh_3 to the allenoate. In contrast, with PMe_3, attempts to locate either the twisted or the E-configured TS were unsuccessful (Scheme S1b). Consequently, the addition proceeds exclusively through the Z-configuration, consistent with previous reports.[1–5] The Z- and E-adducts are identified as energy minima for both catalysts. The twisted isomer observed in the case of PPh_3 is not an energy minimum when PMe_3 is used as the catalyst.

Scheme S1: Comparison of addition pathways using a) PPh_3 and b) PMe_3 as the catalysts. Free energies (ΔG in kcal mol^{-1}) are given relative to the separated reactants.

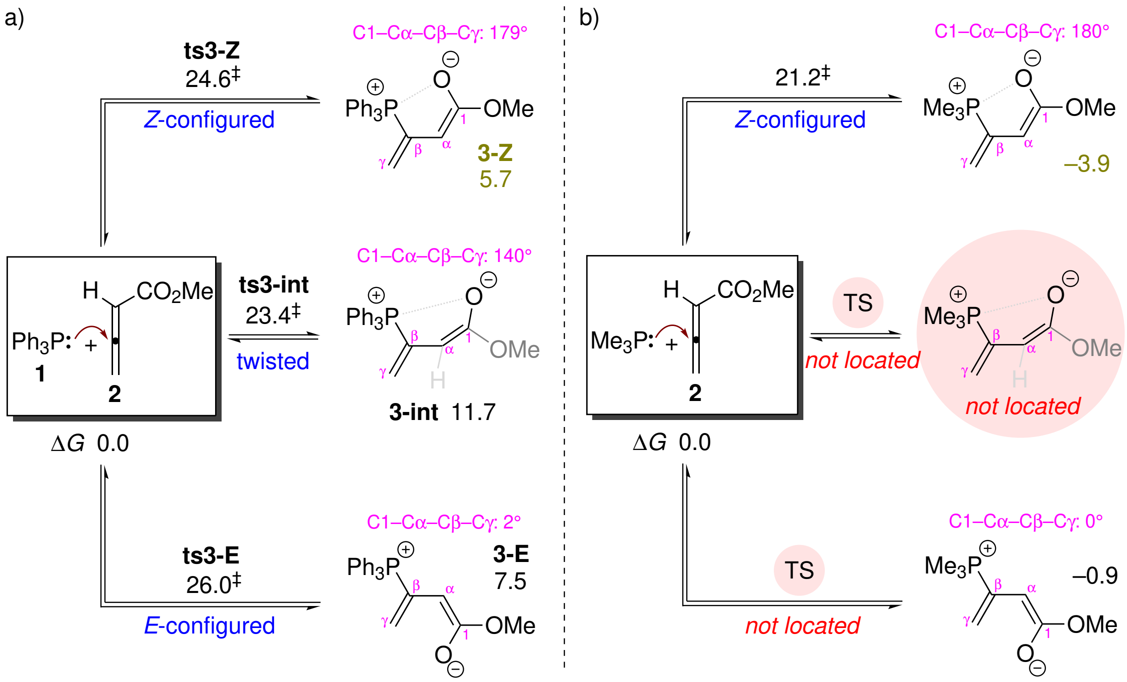

2 Computational workflow

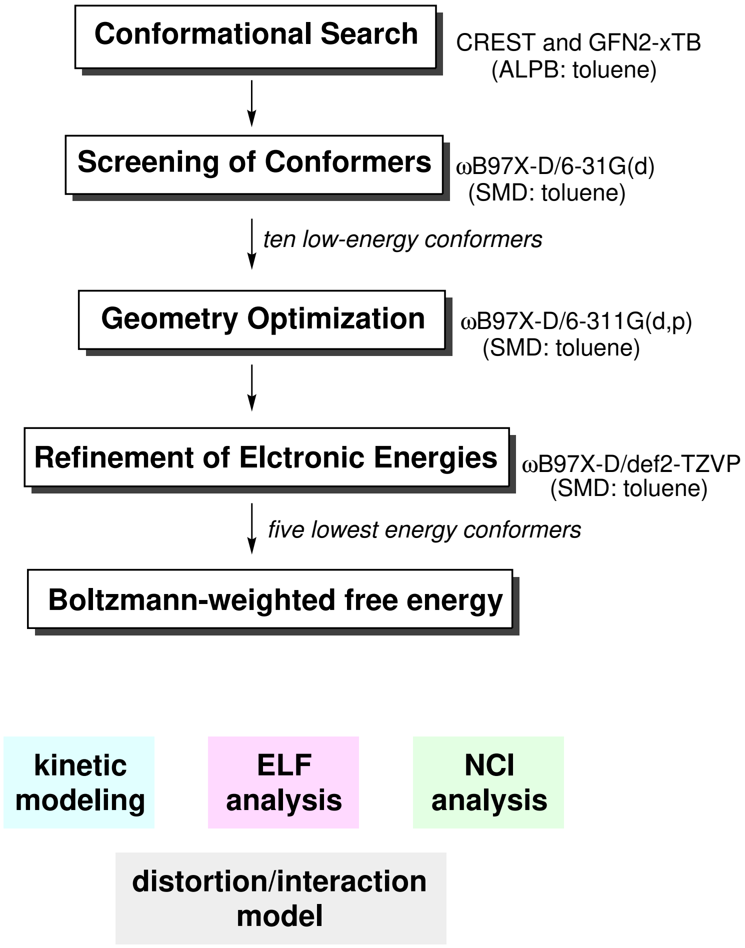

Figure S1: Computational workflow and approaches to data analysis.

Computational methods and programs:

CREST,[6,7] ALPB,[8] GFN2-xTB,[9,10] ωB97X-D,[11] 6-31G(d),[12] def2-TZVP,[13] SMD,[14] quasi-RRHO,[15] ELF,[16] NCI,[17] Multiwfn,[18] Shermo,[19] Gaussian 09.[20]

2.1 Conformational search by CREST

The CREST program was employed to generate possible conformers.[6,7] Solvation effects were incorporated during conformer sampling using the analytical linearized Poisson-Boltzmann (ALPB) model, which has been specifically parameterized for the GFN family of methods.[8] Additional constraints were applied to locate TSs and species that deviated significantly from DFT-optimized structures. These structural constraints were imposed according to DFT-computed geometries. To effectively sample the configuration space of the TSs, the bond to be formed or cleaved was constrained during the CREST search, and the constrained parameters for the TSs are summarized in Table S1. The intermediates in the catalytic cycle exist as phosphonium cations. DFT calculations predict that the phosphonium center adopts a tetrahedral geometry in a four-coordinate environment, and tends to be trigonal-bipyramidal in a five-coordinate state (as in the case of the Z-adduct). However, at the GFN2-xTB[10] level, significant deviations from this arrangement were sometimes observed, particularly when electrostatic P···O interactions were involved. To address this issue, constraints were applied during the conformational search of all intermediates to ensure the reliability of structures.

As shown in the computational workflow of Figure 2, the purpose of the single-point ωB97X-D/6-31G(d) calculations was to efficiently screen low-energy conformers generated by CREST. These screened conformers were then re-optimized at the ωB97X-D/6-311G(d,p) level of theory. Because different conformers may converge to the same structure during DFT geometry optimization, it was necessary to verify whether the optimized structures were distinct. The five most stable conformers were then used to compute Boltzmann-weighted free energies.

Table S1: Number of the conformers and the constrained parameters for the TSs.

reaction	TS	number of conformers	constrained parameters
**1 + 2 + e-iii**			
initial addition	**ts3-Z**, **ts3-EZ**, **ts3-E**	21	P···Cβ: 2.32 Å
Michael addition	**ts4-Za**	41	Cα···C3: 2.23 Å
	**ts4-Zg**	223	Cγ···C3: 2.22 Å
	**ts4-Ea**	40	Cα···C3: 2.10 Å
	**ts4-Eg**	158	Cγ···C3: 2.10 Å
Ring closure	**ts5-Eg** (*syn*)	64	Cα···C4: 2.20 Å
	**ts5-Eg** (*anti*)	155	Cα···C4: 2.20 Å
**1 + 2 + e-iv**			
Michael addition	**ts4-Za′**	132	Cα···C3: 2.23 Å
	**ts4-Zg′**	89	Cγ···C3: 2.25 Å
	**ts4-Ea′**	216	Cα···C3: 2.10 Å
	**ts4-Eg′**	121	Cγ···C3: 2.10 Å
**1 + 8 + e-iii**			
initial addition	**trans-ts3-Z**, **trans-ts3-EZ**, **trans-ts3-E**	56	P···Cβ: 2.33 Å
	**cis-ts3-Z**, **cis-ts3-EZ**, **cis-ts3-E**	100	P···Cβ: 2.30 Å
Michael addition	**trans-ts4-Za**	138	Cα···C3: 2.24 Å
	**trans-ts4-Zg**	123	Cγ···C3: 2.24 Å
	**trans-ts4-Ea**	387	Cα···C3: 2.23 Å
	**trans-ts4-Eg**	263	Cγ···C3: 2.25 Å
Ring closure	**trans-ts5-Za** (*syn*)	112	Cγ···C4: 2.26 Å
	**trans-ts5-Za** (*anti*)	103	Cγ···C4: 2.26 Å

3 Assessment of basis sets and functionals

3.1 Effects of diffuse functions

To verify the effect of diffuse functions on the energetics, single-point calculations were done for adduction formation and Michael addition, and the computed electronic energies are listed in Table S2. In addition to the Pople-type basis set, the Ahlrichs-type basis sets of def2-TZVP and ma-def2-TZVP were also tested. The "ma" prefix in ma-def2-TZVP denotes minimal augmentation, referring to the inclusion of a minimal set of diffuse functions.[21] This basis set has been widely recognized as reliable for DFT-level calculations of barrier heights and electron affinities.[21] Given its size and quality, ma-def2-TZVP is considered the reference standard for benchmarking the accuracy of the other basis sets evaluated. The results in Table S2 show that the impact of diffuse functions is less pronounced when using the Ahlrichs-type triple zeta basis sets, yielding deviations within ± 0.3 kcal mol^{-1} for the ten states. This suggests that the def2-TZVP basis set is sufficient to provide an accuracy comparable to that of ma-def2-TZVP. In contrast, the Pople-type basis sets of 6-311G(d,p) and 6-311++G(d,p) exhibit larger deviations of ± 1.3 kcal mol^{-1}, indicating greater sensitivity to diffuse function inclusion. Moreover, the relative energies obtained with 6-311++G(d,p) do not converge as closely to those from ma-def2-TZVP. The tests demonstrate that def2-TZVP provides satisfactory accuracy in describing the computed energetics.

Table S2: Effects of diffuse functions on energetics for initial addition. The reported electronic energies (in kcal mol^{-1}) are given relative to the energy of the separated reactants (**1** + **2** + **e-iii**).

	6-311G(d,p)	6-311++G(d,p)	def2-TZVP	ma-def2-TZVP
**ts3-int**	9.3	9.7	10.2	10.3
**3-int**	−1.5	−1.5	−3.1	−3.1
**ts3-rotZ**	1.2	1.1	−0.4	−0.4
**3-Z**	−6.9	−7.2	−9.4	−9.4
**ts3-rot**	6.7	5.4	4.1	3.9
**3-E**	−4.1	−5.2	−7.1	−7.5
**ts4-Za**	−13.9	−12.9	−12.1	−11.9
**ts4-Zg**	−13.8	−12.8	−12.3	−12.0
**ts4-Ea**	−14.7	−13.6	−12.6	−12.5
**ts4-Eg**	−18.9	−17.6	−16.7	−16.6

3.2 Tests of other functionals

Single-point energy calculations at the B3LYP-D3[22–26] and M06-2X[27] levels were performed on the three isomeric TSs for initial addition, **ts3-Z**, **ts3-E**, and **ts3-int**. The lowest-energy conformer of each TS was selected for comparison (Table S3). Across all three functionals examined, the twisted TS (**ts3-int**) is consistently predicted to be the most energetically favorable. Similarly, four regioisomeric TSs, **ts4-Za**, **ts4-Zg**, **ts4-Ea**, and **ts4-Eg**, are also examined (Table S4). Calculations employing the three functionals consistently show that **ts4-Eg** has the lowest activation energy, at least 2.7 kcal mol^{-1} lower than the other three modes.

Table S3: Relative electronic energies ($\Delta\Delta E_{\text{ele}}$ in kcal mol^{-1}) of the three isomeric TSs, **ts3-Z**, **ts3-E**, and **ts3-int**. The single-point calculations are performed using the def2-TZVP basis set. The reported electronic energies are given relative to the energy of **ts3-int**.

	ωB97X-D	B3LYP-D3	M06-2X
**ts3-Z**	1.6	1.3	1.6
**ts3-E**	2.5	2.1	2.6
**ts3-int**	0.0	0.0	0.0

Table S4: Relative electronic energies ($\Delta\Delta E_{\text{ele}}$ in kcal mol^{-1}) of the four regioisomeric TSs, **ts4-Za**, **ts4-Zg**, **ts4-Ea**, and **ts4-Eg**. The single-point calculations are performed using the def2-TZVP basis set. The reported electronic energies are given relative to the energy of **ts4-Eg**.

	ωB97X-D	B3LYP-D3	M06-2X
**ts4-Za**	4.6	3.8	4.5
**ts4-Zg**	4.4	2.7	4.6
**ts4-Ea**	4.1	4.8	4.6
**ts4-Eg**	0.0	0.0	0.0

3.3 DLPNO-CCSD(T) calculations

To assess the accuracy of the energetics computed at the ωB97X-D level of theory, the free energy profiles for adduct formation and isomerization were re-evaluated using the high-level DLPNO-CCSD(T) method[28,29] according to the following equation:

$$G_{\mathrm{CC,\ sol}} = G_{\mathrm{DFT,\ sol}} - E_{\mathrm{DFT,\ gas}} + E_{\mathrm{CC,\ gas}},$$

where $E_{\mathrm{DFT,\ gas}}$ and $E_{\mathrm{CC,\ gas}}$ denote the gas-phase electronic energies computed at the DFT and DLPNO-CCSD(T)/def2-TZVP levels, respectively. In this scheme, only the electronic energy was replaced; all other energetic components including zero-point energies, thermal corrections, and solvation effects were retained from the original DFT calculations. The coupled-cluster calculations were performed using the ORCA software package.[30] Density fitting was employed to accelerate the evaluation of two-electron integrals.

Figure S2 presents a comparison of the free energy profiles based on the DFT-optimized lowest-energy conformers. It should be noted that the primary difference between the DFT-computed energies shown in Figure S2 and those in Scheme 5 lies in the number of conformers considered: in Scheme 5 of the main context, free energies were computed using the five lowest-energy conformers for each species. Although the coupled-cluster energies are, on average, 1.4 kcal mol^{-1} higher than those computed at the DFT level, the two methods exhibit consistent energetic trends overall, supporting the reliability of the ωB97X-D functional for describing the energetics of phosphine-associated reactions.

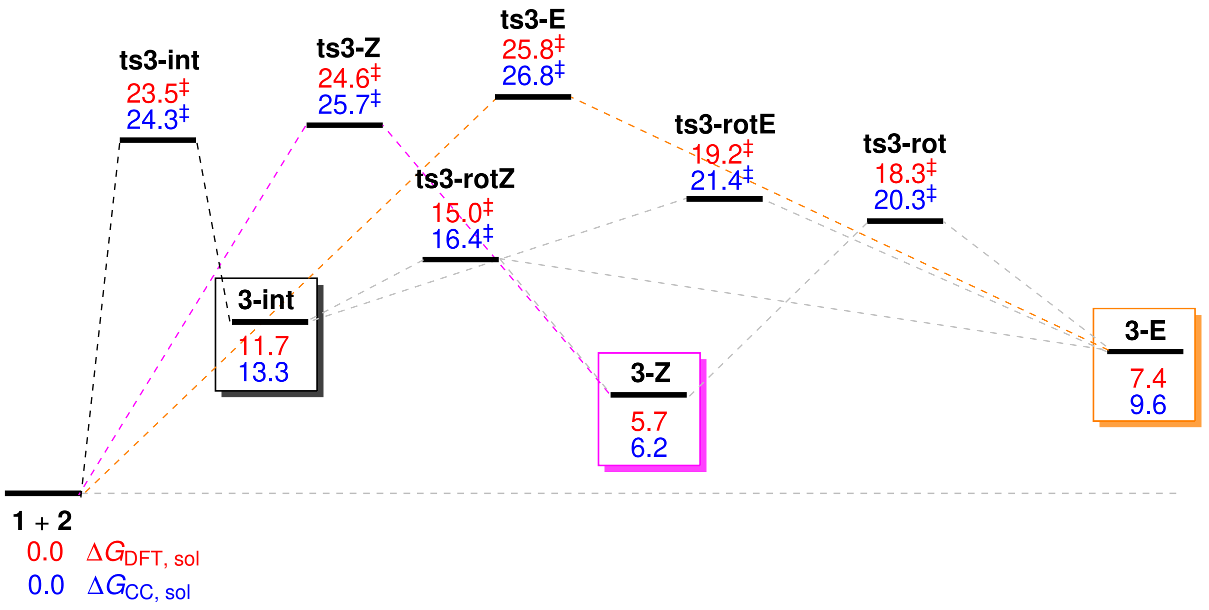

Figure S2: Free energy profiles ($\Delta G_{\text{DFT, sol}}$ and $\Delta G_{\text{CC, sol}}$ in kcal mol^{-1}) based on the most stable conformers. Values shown in red and blue correspond to free energies computed at the ωB97X-D and DLPNO-CCSD(T) levels, respectively.

4 Adduct formation

4.1 Isomeric TSs for initial addition

For nucleophilic addition of PPh_3 to allenoate **2**, six isomeric TSs arising from combinations of *trans* and *cis* configurations with *Z*-, *E*-, and twisted geometries are examined. Among these, the initial addition is favored with the *s-cis* conformation, which exhibits the lowest activation barrier of 23.4 kcal mol^{-1}, compared to 24.2 kcal mol^{-1} for the *s-trans* isomer.

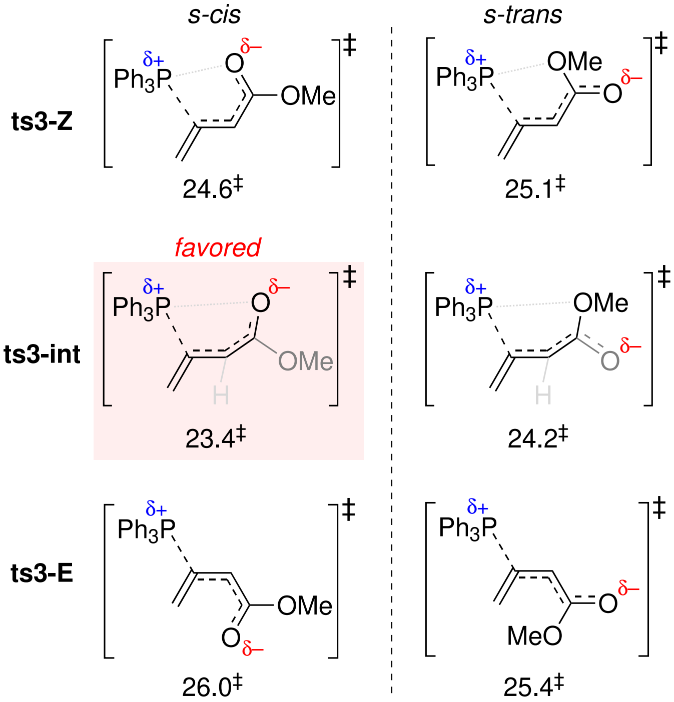

Figure S3: Isomeric TSs for nucleophilic addition of PPh_3 to *s-cis*/*s-trans* allenoate **2**. Computed activation free energies ($\Delta G^\ddagger$ in kcal mol^{-1}) are reported with respect to the separated reactants.

4.2 Rotational barrier and IRC calculations

Rotational barrier calculations followed the same procedure as that for calculating the barrier for bond forming/breaking processes. The first step was to optimize the TS corresponding to the desired torsional mode. The synchronous transit-guided quasi-Newton method[31] (Gaussian keyword: QST3) was employed to locate the first-order saddle point: three initial guess structures along the reaction coordinate were provided in the input file. After geometry optimization, the vibrational mode of the imaginary frequency (one and only one) must be confirmed to correspond to the desired reaction coordinate. Unlike bond forming/breaking courses, internal rotation typically features a low frequency, reflecting a relatively flat potential energy surface around the TS. Intrinsic reaction coordinate (IRC) calculations were performed to verify that the located TSs properly connect the corresponding reactants and products (Figure S4). In some cases, it may be more efficient to use a larger step size (e.g., 10 to 20) to obtain the first point, as in the case of **ts3-rotZ**. Subsequent IRC steps were then continued using the default step size of 10 in the Gaussian package (i.e., 0.1 Bohr). To ensure that the endpoints of the IRC calculations are energy minima, geometry optimization followed by frequency calculations was carried out for these endpoint structures. In addition to the IRC calculations, relaxed potential energy scans were done to characterize the low-frequency torsional vibration mode (Figure S5). These scans require less computational effort if the variable of the reaction coordinate was appropriately chosen.

In principle, the rotational barrier is defined as the energy difference between the TS and its associated reactant for a given elementary step. However, if not clearly defined, this “reactant” may be confused with either the initial reactant of the overall reaction or its corresponding adduct intermediate. To avoid such ambiguity, all free energies reported in the revised manuscript are referenced relative to the energy of the initially separated reactants (i.e., PPh_3 + allenoate + enone).

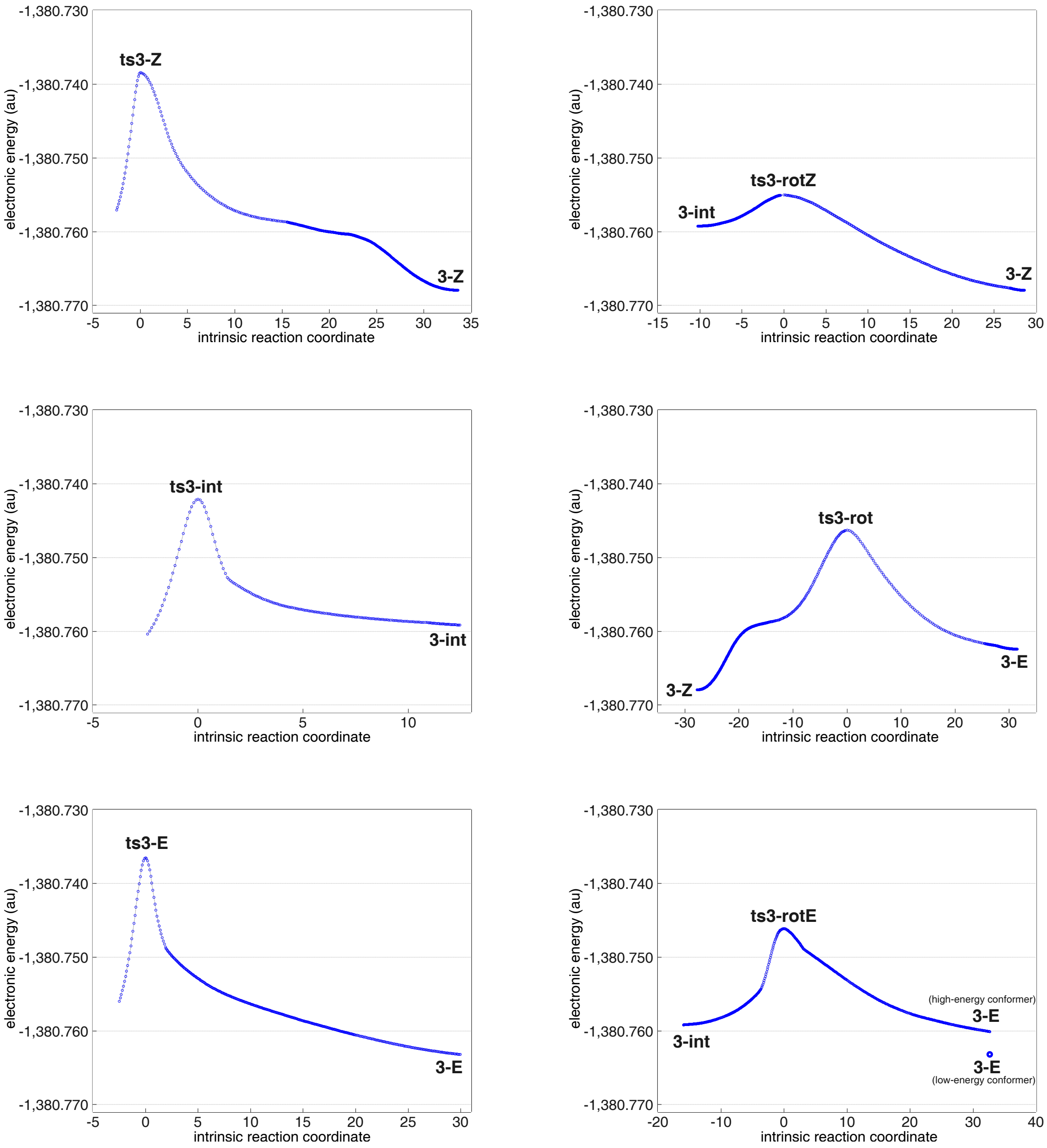

Figure S4: IRC calculations of initial addition and adduct isomerization. The units of the x- and y-axes are intrinsic reaction coordinate ($amu^{1/2}$ bohr) and electronic energies (au), respectively.

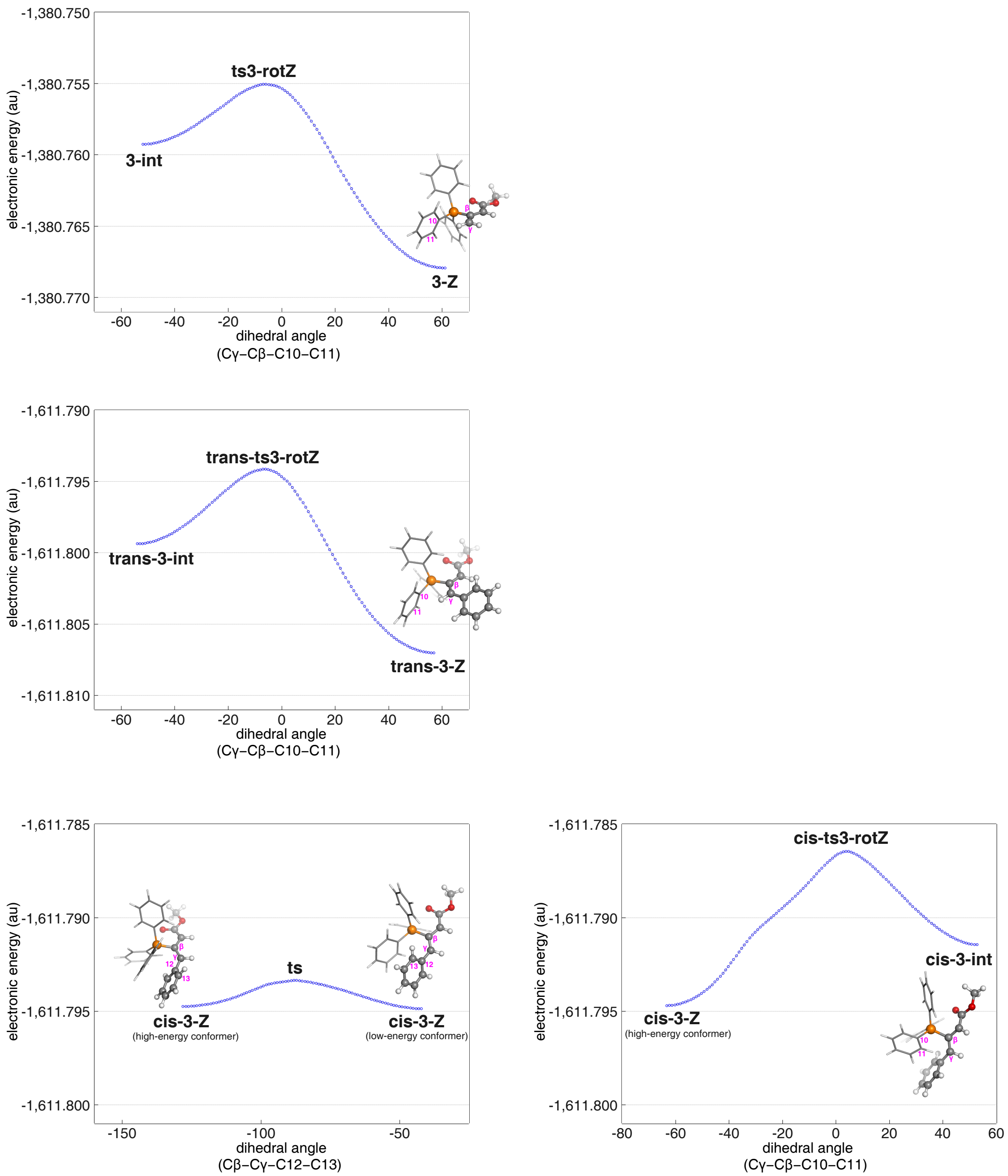

Figure S5: Scan calculations with respect to the specified dihedral angle. The units of the x- and y-axes are dihedral angle (°) and electronic energies (au), respectively.

4.3 Comparison of bonding and geometric properties

Figure S6 shows calculated bond lengths and electron populations of the conjugated double bonds in the six molecules, including two diene conformers, an allyl anion, and three adduct isomers. The electron population of the valence basin $V(X, Y)$ between two atoms is determined using the electron localization function (ELF).[32] In the diene system, the single bond connecting two conjugated double bonds yields an electron population of approximately 2.20 e, while each double bond accounts for about 3.38 e. The allyl anion shows a maximum electron population of 3.68 e in the valence basins V(C4,C3)/V(C3,C2). According to the computed electron populations, the bonding patterns of the allenic moiety in **3-Z** and **3-E** resemble those of *s-trans* and *s-cis* dienes more closely than that of an allyl anion, indicating a single-bond character between Cα and Cβ in the adducts. Thus, by analogy to the exchange between the *s-trans* and *s-cis* dienes, the adduct isomers can interconvert through internal rotation around the Cα−Cβ bond. Among the three adducts, the twisted form (**3-int**) displays the lowest electron population of 2.27 e in the valence basin V(Cα,Cβ), suggesting weaker resonance stabilization between the C1=Cα and Cβ=Cγ double bonds.

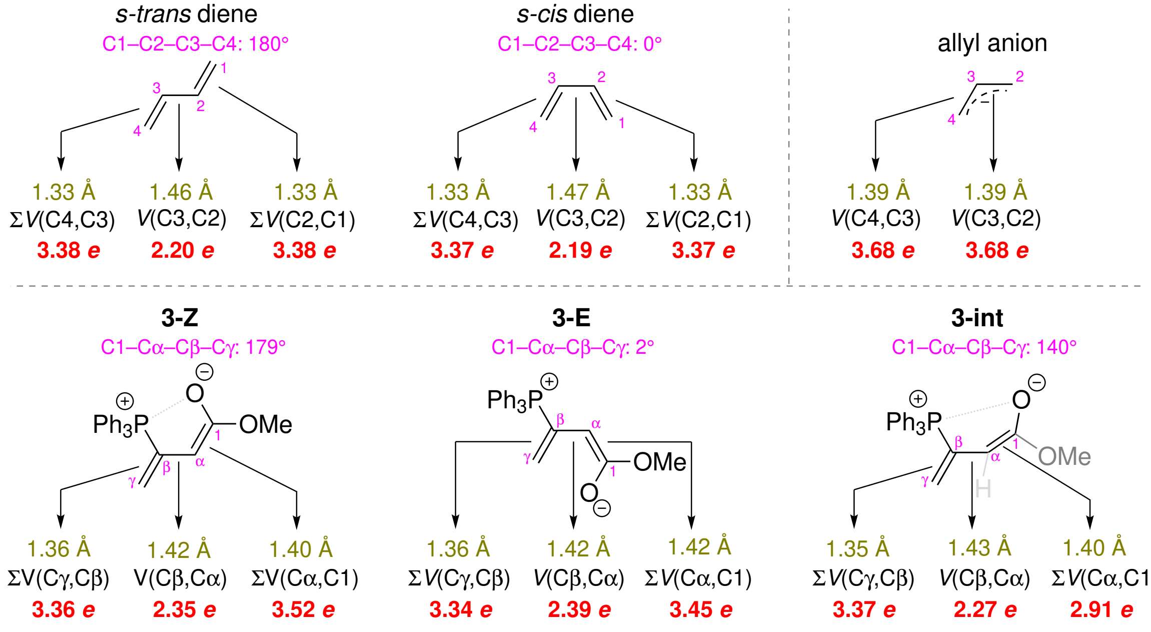

Figure S6: Calculated C−C bond lengths and electron populations of the corresponding valence basins (shown in bold red).

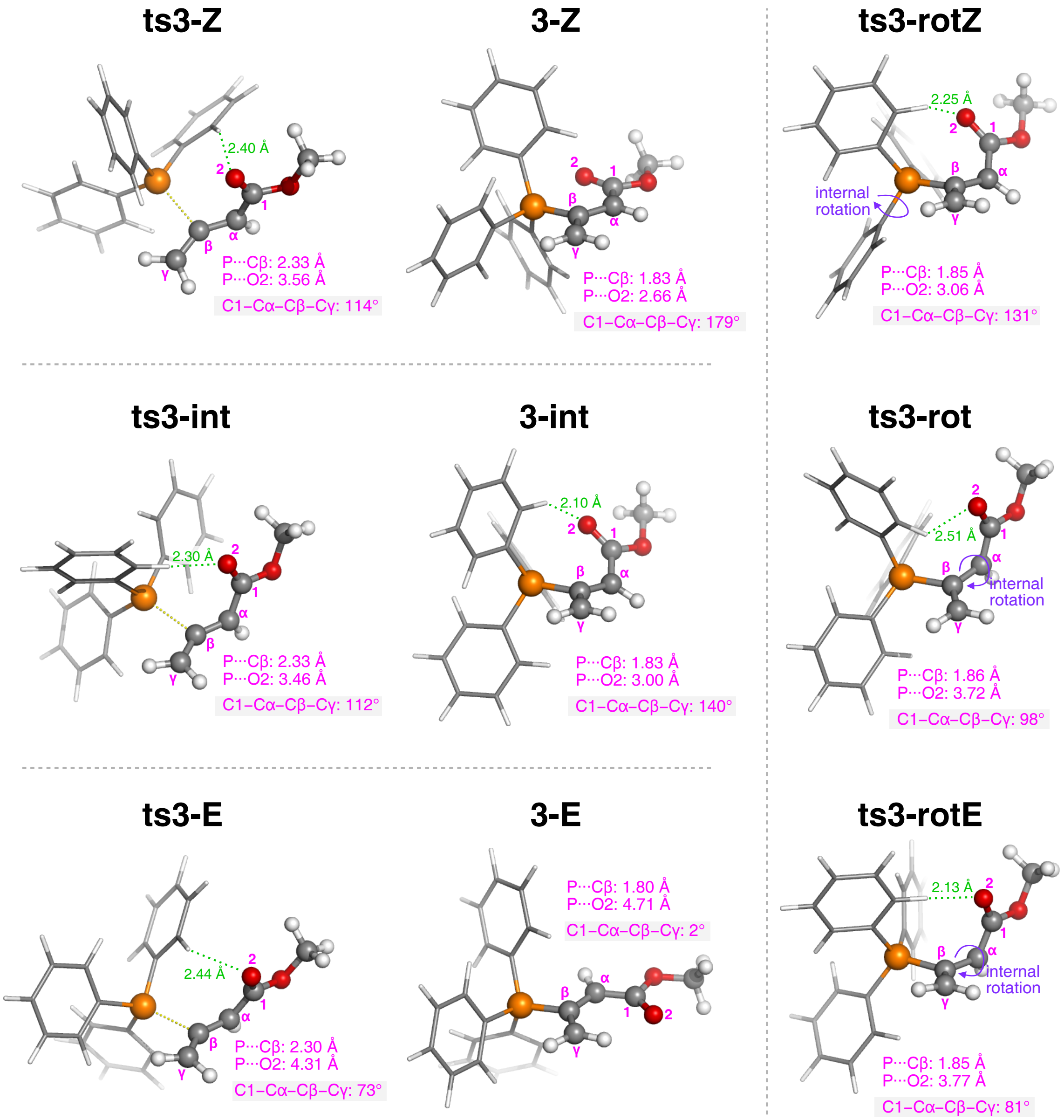

Figure S7: Optimized structures of initial addition of PPh_3 to unsubstituted allenoate **2**. Selected structural parameters with respect to the allenic moiety are listed in magenta. The noncovalent interactions of CH···O are represented by green dashed lines.

4.4 Identification of key interactions by NCI analysis

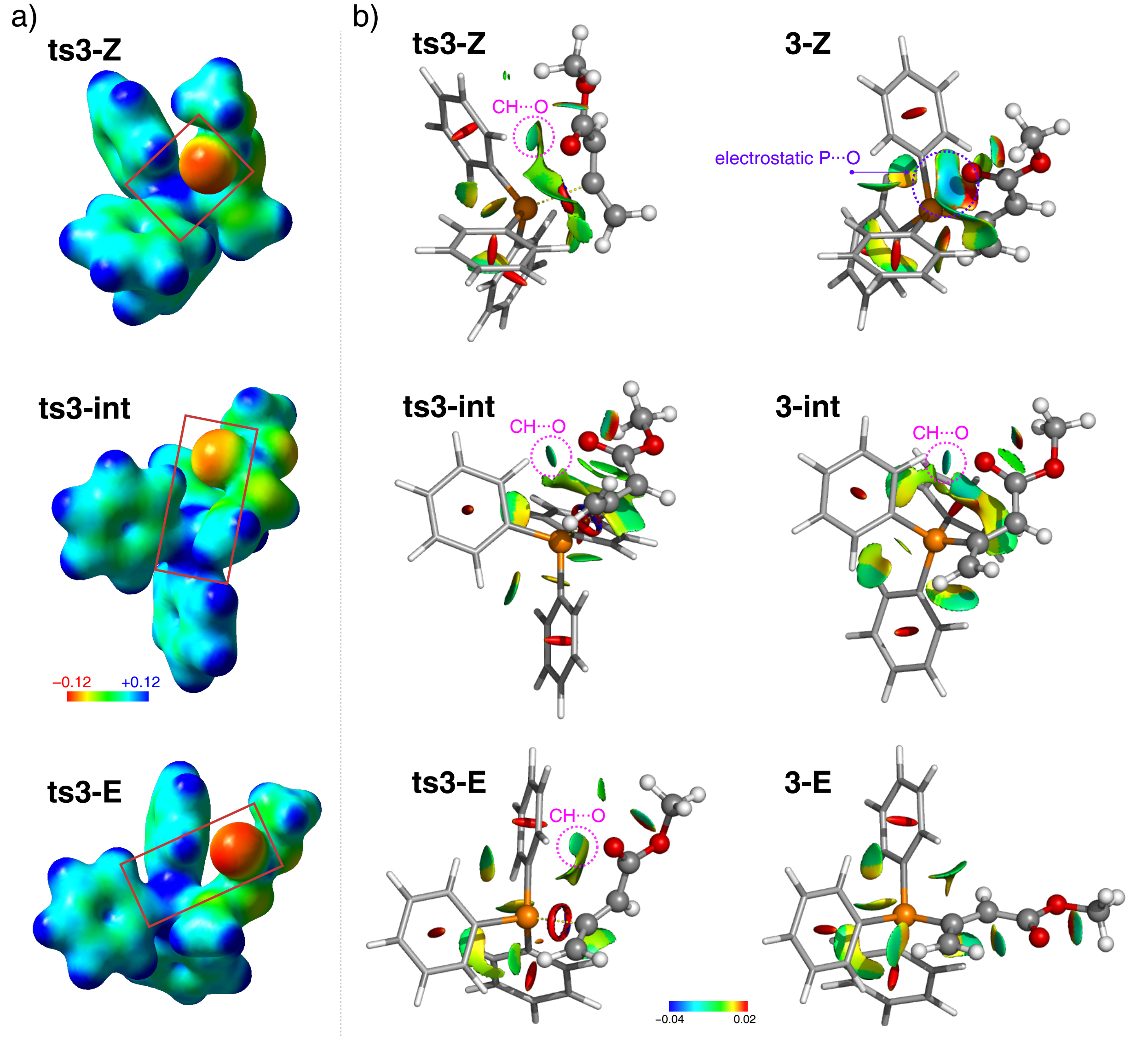

Figure S8: a) Electrostatic potential mapped on an electron density isosurface of 0.02 a.u., and b) NCI analysis of the lowest energy conformers for initial addition. The NCI surface corresponds to $s = 0.5$ au and the NCI color scale ranges from -0.04 to 0.02 au.

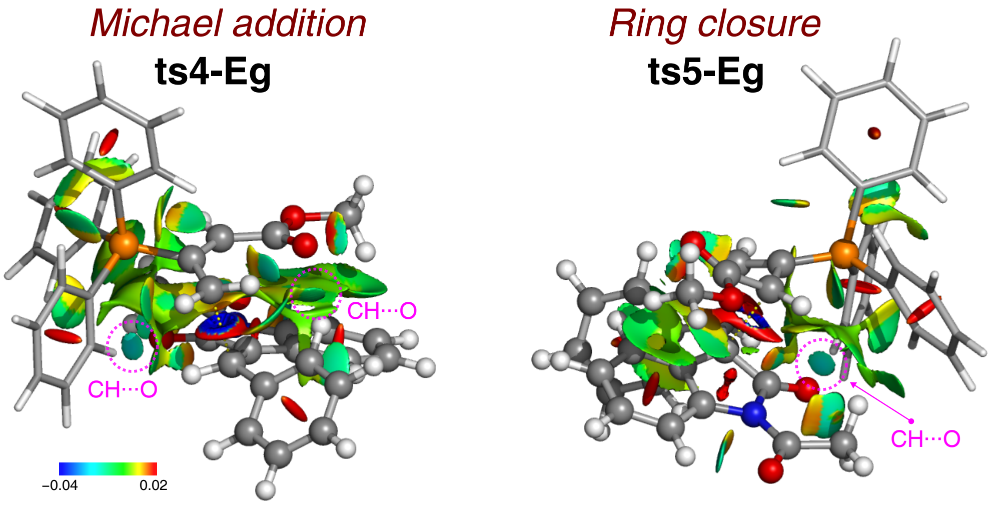

Figure S9: NCI analysis of the lowest energy TS conformers in the [3+2] cyclization of **2** and **e-iii**. The NCI surface corresponds to $s = 0.5$ au and the NCI color scale ranges from -0.04 to 0.02 au.

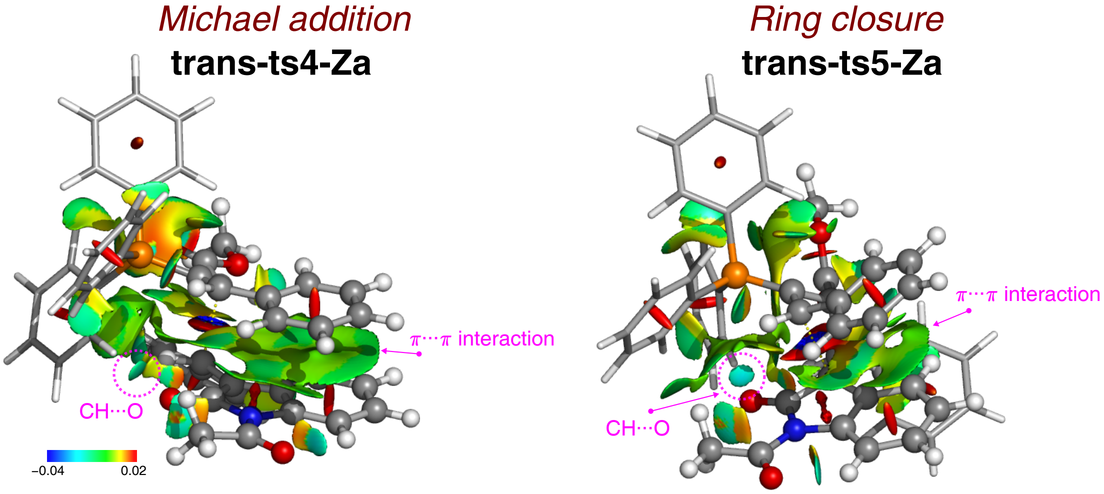

Figure S10: NCI analysis of the lowest energy TS conformers in the [3+2] cyclization of **8** and **e-iii**. The NCI surface corresponds to $s = 0.5$ au and the NCI color scale ranges from -0.04 to 0.02 au.

4.5 Frontier orbital interactions

As illustrated in Figure S11c, two regions of orbital overlap are identified in the favored TS **ts4-Eg**: (1) the primary interaction between the 1,3-dipole of the allenic moiety and the alkene of the enone, and (2) the secondary interaction between the enolate and the oxindole ring. Such a second orbital interaction is lacking in the other three disfavored TSs, **ts4-Za**, **ts4-Zg**, and **ts4-Ea**.

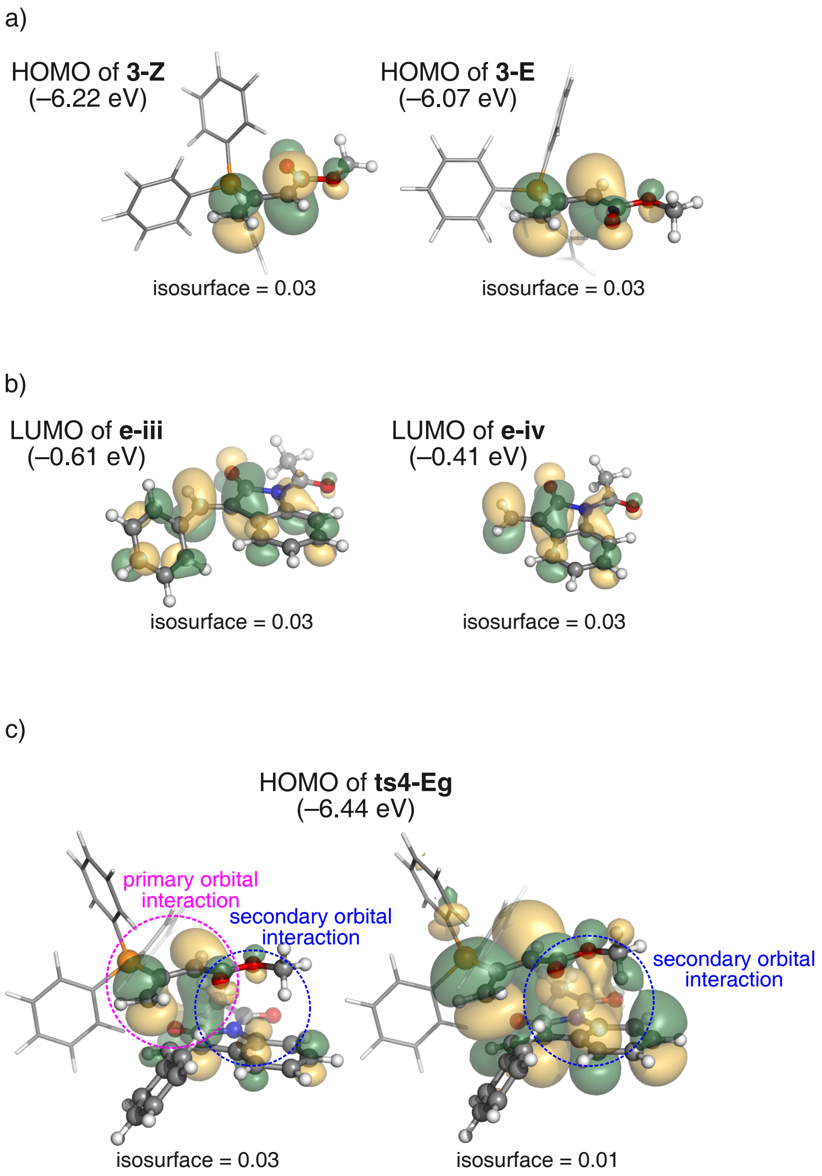

Figure S11: Frontier molecular orbitals of a) adducts **3-Z**/**3-E**, b) enone **e-iii**/**e-iv**, and c) the favored TS **ts4-Eg**. To better visualize the secondary orbital interaction, the isosurface of its HOMO is displayed at a reduced value of 0.01.

4.6 Michael addition of 3-Z/3-E to e-iii

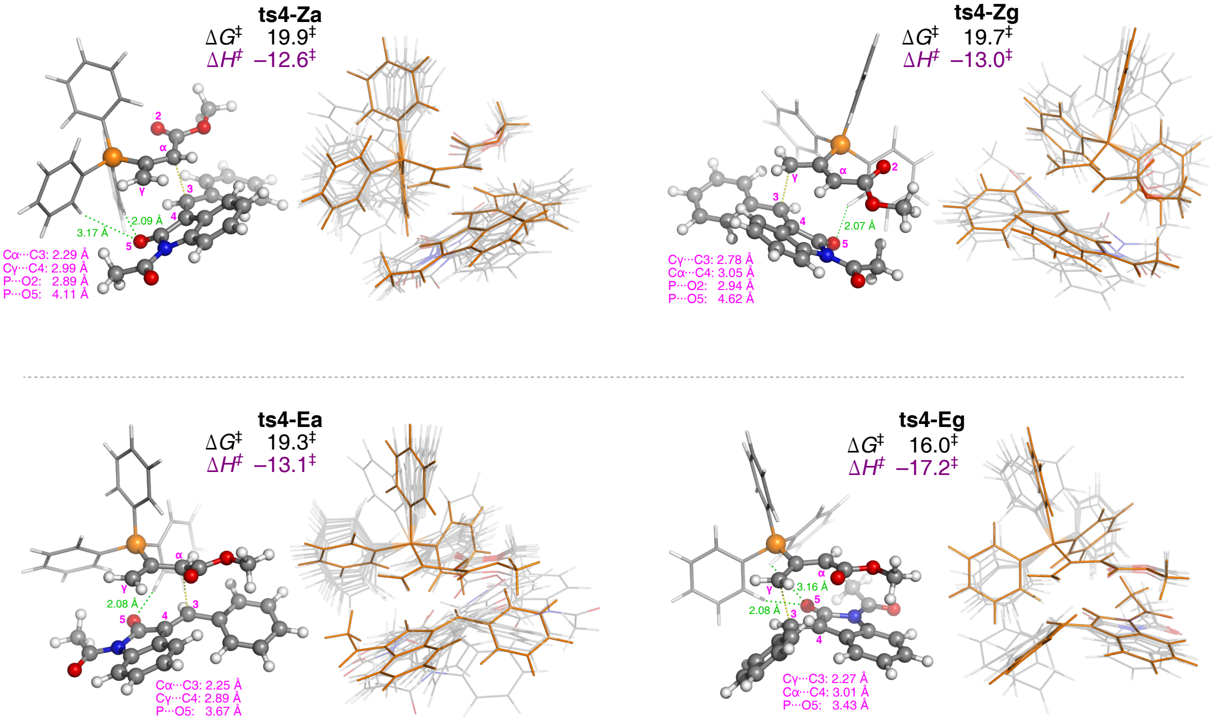

Figure S12: Optimized structures of Michael addition of **3-Z/3-E** to **e-iii**. Key structural parameters of the lowest-energy conformers are shown in magenta, and noncovalent interactions are indicated by green dashed lines. Free energies and enthalpies (in kcal mol^{-1}) are reported relative to the separated reactants. All conformers for each mode are compared by aligning the four atoms of P, Cα, Cβ, and Cγ, with the most stable conformers highlighted in orange.

4.7 Ring closure in *syn* vs *anti* modes

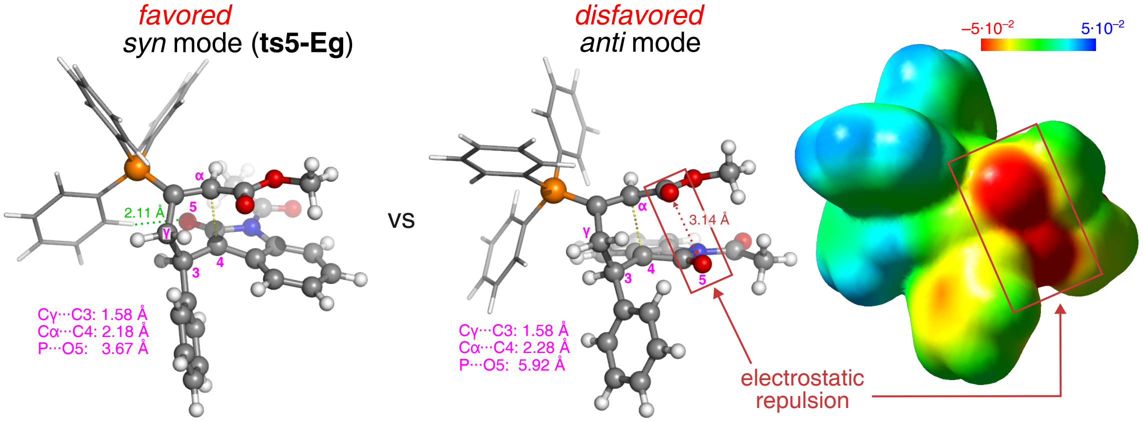

Figure S13: TS structures associated with facial selectivity in the ring-closure step. In the lowest-energy conformer, key structural parameters are shown in magenta, and noncovalent interactions are indicated by green dashed lines. The electrostatic potential (units in a.u.) mapped onto an electron density isosurface of 10^{-4} a.u.

4.8 Michael addition of 3-Z/3-E to e-iv

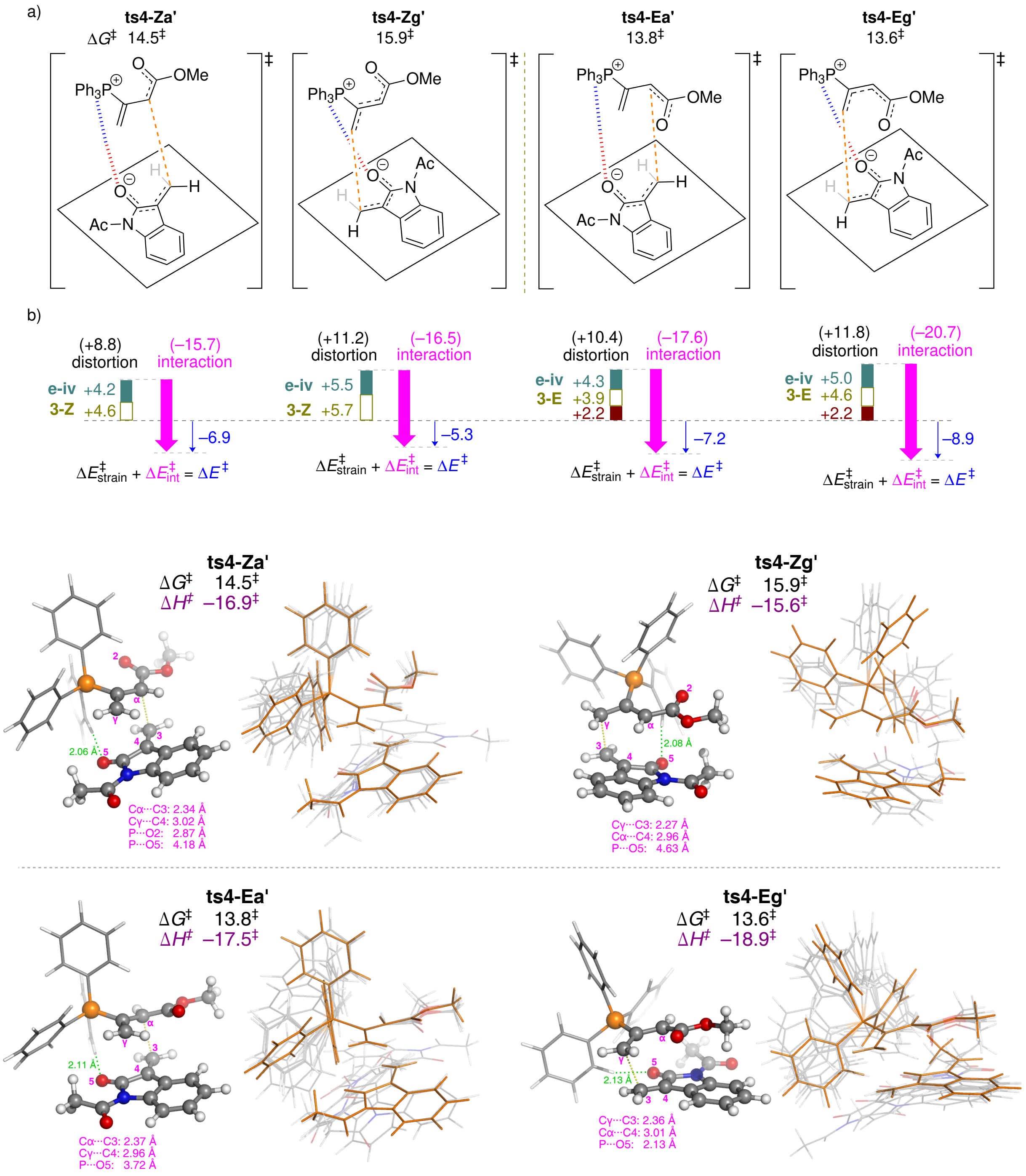

Figure S14: a) Four modes of Michael addition of **3-Z/3-E** to **e-iv**. b) Analysis of the distortion-interaction model based on electronic energies (kcal mol^{-1}). Optimized TS structures are shown at the bottom, with the most stable conformers highlighted in orange. Free energies and enthalpies (in kcal mol^{-1}) are reported relative to the separated reactants.

5 Reactions of γ-substituted allenoates

Figures S15 and S16 show the optimized structures for addition of PPh_3 to β-substituted allenoate **8**, depicted in Scheme 6. Optimized TS structures of the cycloaddition reaction are depicted in Figure S17. Among the four possible modes of Michael addition, **trans-ts4-Za** exhibits the lowest activation energy (Figure S17a). In the two TSs derived from **trans-3-E** (**trans-ts4-Ea** and **trans-ts4-Eg**), significant steric congestion is observed between the γ-phenyl group and the ester moiety of the allenoate. Furthermore, the *syn*-mode of ring closure is energetically preferred over the *anti*-mode (Figure S17b).

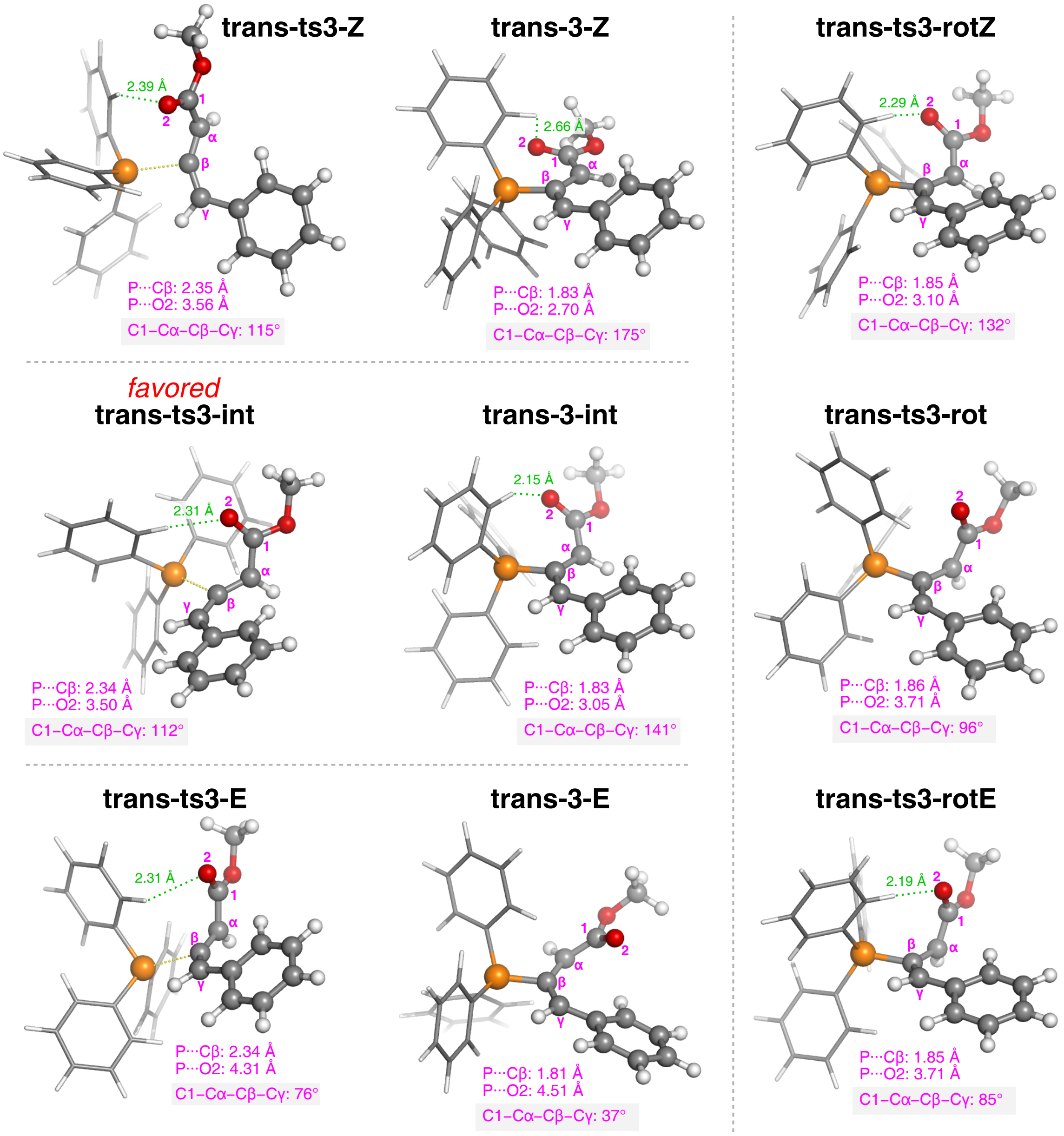

Figure S15: Optimized structures of initial addition of PPh_3 to γ-substituted allenoates in terms of the *trans*-configuration. Selected structural parameters with respect to the allenic moiety are listed in magenta. The noncovalent interactions of CH···O are represented by green dashed lines.

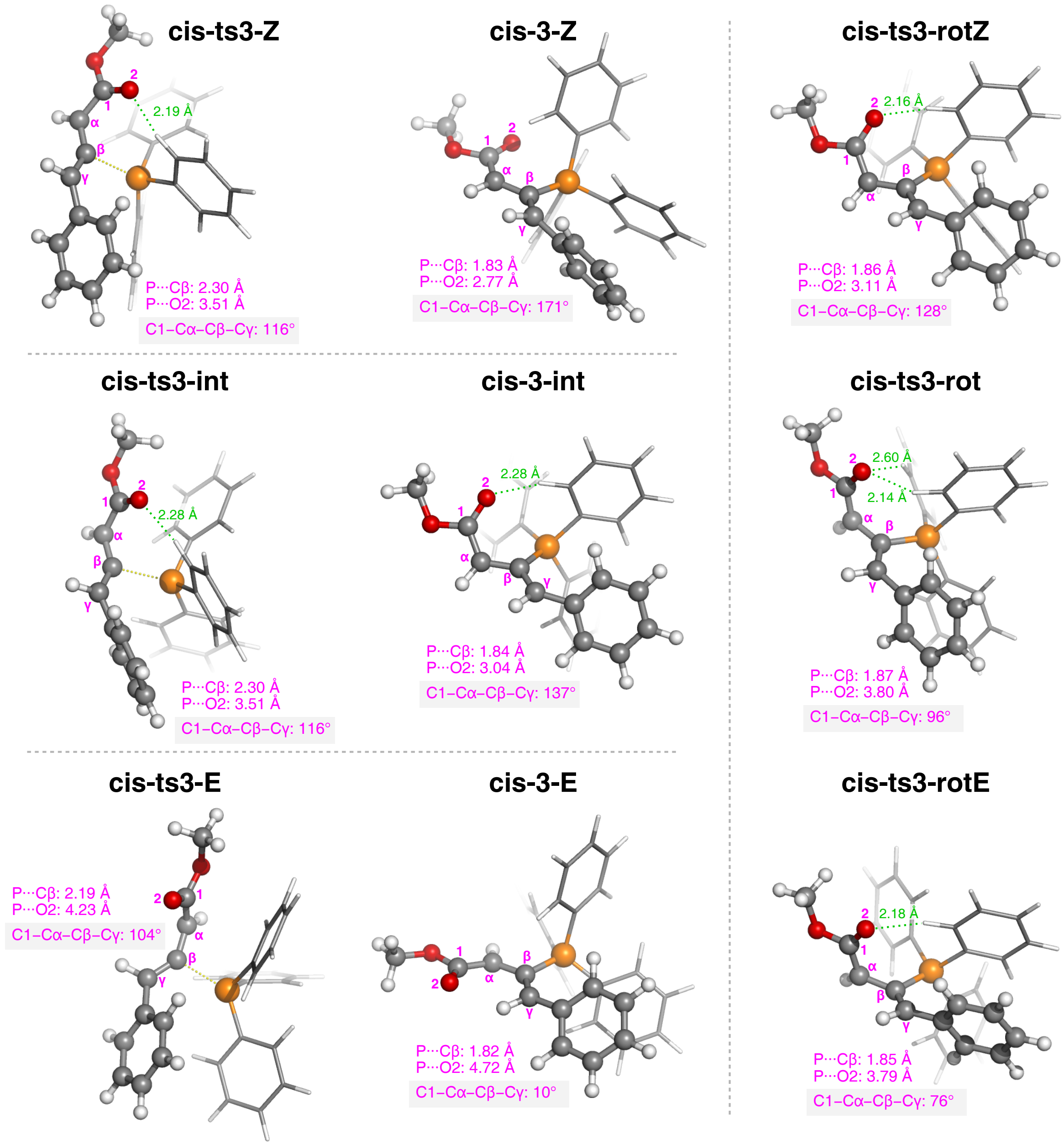

Figure S16: Optimized structures of initial addition of PPh_3 to γ-substituted allenoates in terms of the *cis*-configuration. Selected structural parameters with respect to the allenic moiety are listed in magenta. The noncovalent interactions of CH···O are represented by green dashed lines.

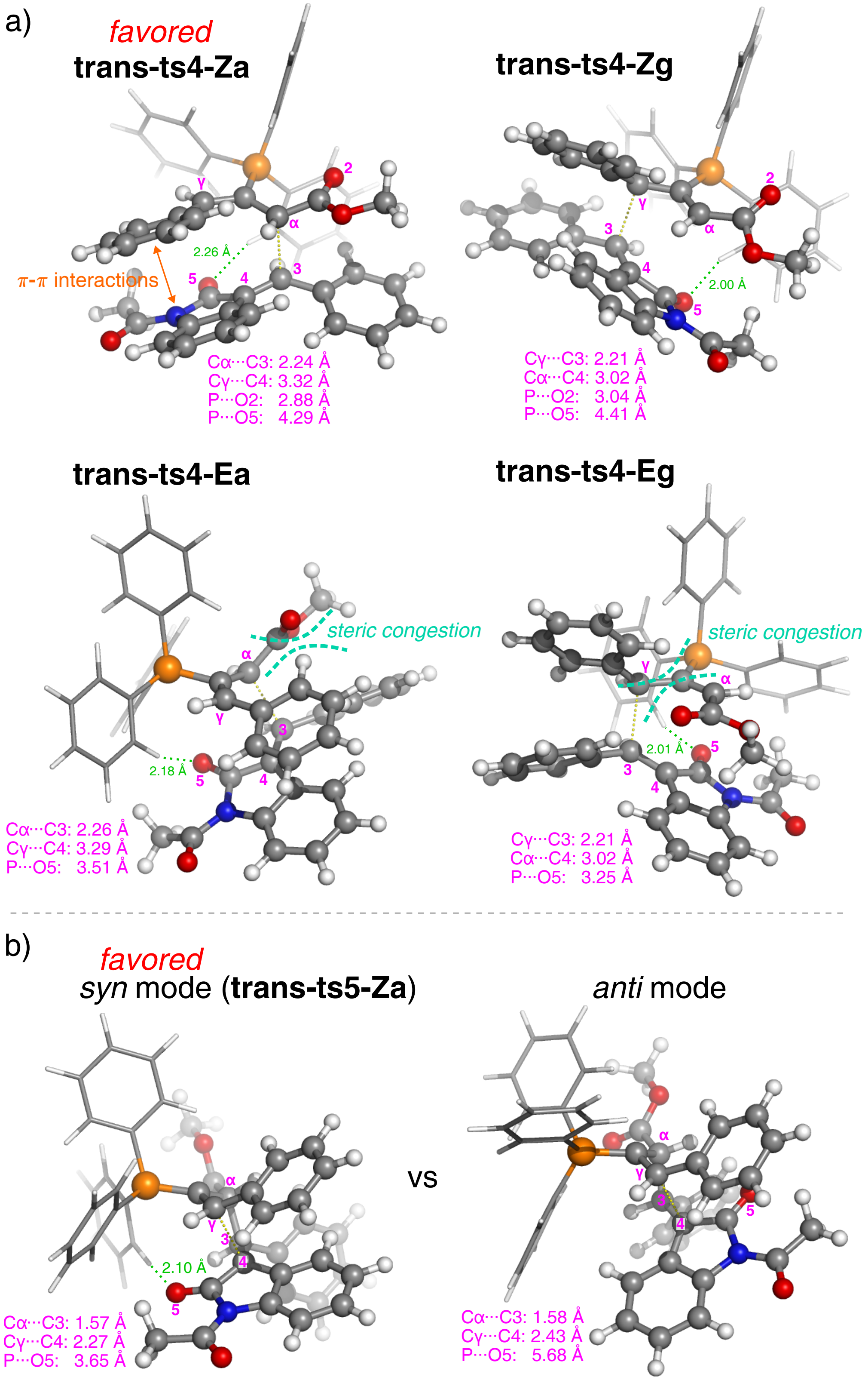

Figure S17: TS structures with respect to a) Michael addition and b) ring closure. Selected structural parameters with respect to the allenic moiety are listed in magenta. The noncovalent interactions of CH···O are represented by green dashed lines.

6 Kinetic simulations

According to the reaction network in Figure 6b, the rate equations are written as

$$\frac{d[\mathbf{1}]}{dt} = -k_{3\mathrm{i}}[\mathbf{1}][\mathbf{2}] + k_{-3\mathrm{i}}[\textbf{3-int}] + k_{7\text{-Za}}[\textbf{5-Za}] + k_{7\text{-Zg}}[\textbf{5-Zg}] + k_{7\text{-Ea}}[\textbf{5-Ea}] + k_{7\text{-Eg}}[\textbf{5-Eg}] \quad (1)$$

$$\frac{d[\mathbf{2}]}{dt} = -k_{3\mathrm{i}}[\mathbf{1}][\mathbf{2}] + k_{-3\mathrm{i}}[\textbf{3-int}] \quad (2)$$

$$\frac{d[\textbf{e-iii}]}{dt} = -k_{4\mathrm{Za}}[\textbf{3-Z}][\textbf{e-iii}] - k_{4\mathrm{Zg}}[\textbf{3-Z}][\textbf{e-iii}] - k_{4\mathrm{Ea}}[\textbf{3-E}][\textbf{e-iii}] - k_{4\mathrm{Eg}}[\textbf{3-E}][\textbf{e-iii}] \quad (3)$$

$$\frac{d[\textbf{3-int}]}{dt} = +k_{3\mathrm{i}}[\mathbf{1}][\mathbf{2}] - k_{-3\mathrm{i}}[\textbf{3-int}] - k_{3\mathrm{rotZ}}[\textbf{3-int}] + k_{-3\mathrm{rotZ}}[\textbf{3-Z}] - k_{3\mathrm{rotE}}[\textbf{3-int}] + k_{-3\mathrm{rotE}}[\textbf{3-E}] \quad (4)$$

$$\frac{d[\textbf{3-Z}]}{dt} = +k_{3\mathrm{rotZ}}[\textbf{3-int}] - k_{-3\mathrm{rotZ}}[\textbf{3-Z}] + k_{-3\mathrm{rot}}[\textbf{3-E}] - k_{3\mathrm{rot}}[\textbf{3-Z}] - k_{4\mathrm{Za}}[\textbf{3-Z}][\textbf{e-iii}] - k_{4\mathrm{Zg}}[\textbf{3-Z}][\textbf{e-iii}] \quad (5)$$

$$\frac{d[\textbf{3-E}]}{dt} = +k_{3\mathrm{rotE}}[\textbf{3-int}] - k_{-3\mathrm{rotE}}[\textbf{3-E}] - k_{-3\mathrm{rot}}[\textbf{3-E}] + k_{3\mathrm{rot}}[\textbf{3-Z}] - k_{4\mathrm{Ea}}[\textbf{3-E}][\textbf{e-iii}] - k_{4\mathrm{Eg}}[\textbf{3-E}][\textbf{e-iii}] \quad (6)$$

$$\frac{d[\textbf{5-Za}]}{dt} = +k_{4\mathrm{Za}}[\textbf{3-Z}][\textbf{e-iii}] - k_{7\text{-Za}}[\textbf{5-Za}] \quad (7)$$

$$\frac{d[\textbf{5-Zg}]}{dt} = +k_{4\mathrm{Zg}}[\textbf{3-Z}][\textbf{e-iii}] - k_{7\text{-Zg}}[\textbf{5-Zg}] \quad (8)$$

$$\frac{d[\textbf{5-Ea}]}{dt} = +k_{4\mathrm{Ea}}[\textbf{3-E}][\textbf{e-iii}] - k_{7\text{-Ea}}[\textbf{5-Ea}] \quad (9)$$

$$\frac{d[\textbf{5-Eg}]}{dt} = +k_{4\mathrm{Eg}}[\textbf{3-E}][\textbf{e-iii}] - k_{7\text{-Eg}}[\textbf{5-Eg}] \quad (10)$$

$$\frac{d[\textbf{7-Za}]}{dt} = +k_{7\text{-Za}}[\textbf{5-Za}] \quad (11)$$

$$\frac{d[\textbf{7-Zg}]}{dt} = +k_{7\text{-Zg}}[\textbf{5-Zg}] \quad (12)$$

$$\frac{d[\textbf{7-Ea}]}{dt} = +k_{7\text{-Ea}}[\textbf{5-Ea}] \quad (13)$$

$$\frac{d[\textbf{7-Eg}]}{dt} = +k_{7\text{-Eg}}[\textbf{5-Eg}] \quad (14)$$

where the concentrations of the regioisomeric products are expressed as

$$[\alpha\text{-regioisomer}] = [\mathbf{7\text{-}Za}] + [\mathbf{7\text{-}Ea}] \tag{15}$$

$$[\gamma\text{-regioisomer}] = [\mathbf{7\text{-}Zg}] + [\mathbf{7\text{-}Eg}]. \tag{16}$$

The initial concentrations of the catalyst and the reactants are set as $[\mathbf{1}]_0$ = 0.03 M, $[\mathbf{2}]_0$ = 0.60 M, and $[\mathbf{e\text{-}iii}]_0$ = 0.30 M, while the initial concentrations of all other species are set to zero. A system of 14 coupled differential equations was solved using the Mathematica software to simulate the reaction kinetics. At full conversion, the computed product ratio of [**7-Za**]:[**7-Zg**]:[**7-Ea**]:[**7-Eg**] is 1 : 1 :< 1 : 98, corresponding to an overall α:γ ratio of 1:99. Table S5 summarizes the results of the kinetic simulation using various substrates.

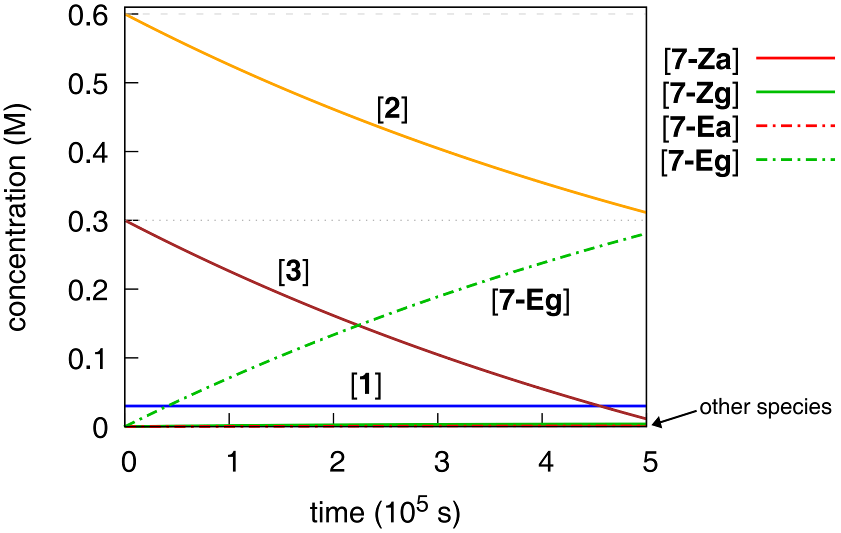

Figure S18: Variation of the concentrations in the kinetic modeling.

Table S5: Initial concentrations and results of kinetic simulations.

substrates	**2** + **e-iii**	**2** + **e-iv**	**8** + **e-iii**
initial concentration	$[\mathbf{1}]_0 = 0.03$ M	$[\mathbf{1}]_0 = 0.03$ M	$[\mathbf{1}]_0 = 0.20$ M
	$[\mathbf{2}]_0 = 0.60$ M	$[\mathbf{2}]_0 = 0.60$ M	$[\mathbf{8}]_0 = 1.50$ M
	$[\textbf{e-iii}]_0 = 0.30$ M	$[\textbf{e-iv}]_0 = 0.30$ M	$[\textbf{e-iii}]_0 = 1.00$ M
product ratio	1:1:<1:98	90:8:1:1	99:1:<1:<1
α:γ ratio	1:99	91:9	99:1

7 Cartesian coordinates of optimized structures

For each species, the coordinates and the electronic energies of the lowest energy conformer are provided. Other energy terms used to calculate free energies include zero-point energies, thermal corrections, and entropy contributions. In addition to the default RRHO treatment implemented in the Gaussian program,[20] the quasi-RRHO approximation[15] proposed by Grimme is employed to correct entropic contributions from low-frequency vibrational modes. The types of corrections are reported below and are labeled as RRHO and quasi-RRHO, respectively. The imaginary frequency with respect to the reaction coordinate is given for each TS located. Note that thermodynamic quantities are calculated under gas-phase standard conditions of 298.15 K and 1 atm, as defined by the default settings in the Gaussian software. In solution phase, a change in concentration (1 M standard state) leads to a difference in free energy of 1.894 kcal mol^{-1} in ΔG_{conc}, which must be included in the calculation of the free energy of solvation. Single-point electronic energies computed with a larger basis set of def2-TZVP are also presented.

1

```
charge, 2S+1    = 0, 1
--- OPT at ωB97X-D/6-311G(d,p) in SMD ---
E(ele)          = -1036.24281192 a.u.
Correction to G = 0.230057 a.u.(RRHO)
Correction to G = 0.233781 a.u.(quasi-RRHO)
--- SP at ωB97X-D/def2-TZVP in SMD ---
E(ele)          = -1036.32943260 a.u.
 P      0.011961    0.003438    1.229839
 C     -1.537204    0.587995    0.422103
 C     -1.573588    1.423505   -0.695037
 C     -2.743684    0.154616    0.979609
 C     -2.790491    1.809215   -1.246627
 C     -3.958089    0.529053    0.420160
 C     -3.983695    1.360246   -0.694350
 H     -0.649802    1.776647   -1.139286
 H     -2.731250   -0.484424    1.857456
 H     -2.803835    2.461203   -2.113208
 H     -4.885252    0.179573    0.860754
 H     -4.931296    1.660941   -1.127390
 C      0.268826   -1.628048    0.413830
 C     -0.434905   -2.063829   -0.709801
 C      1.238449   -2.467520    0.969823
 C     -0.167461   -3.308509   -1.269116
 C      1.513987   -3.704968    0.403175
 C      0.808919   -4.129081   -0.717777
 H     -1.195660   -1.431291   -1.153224
 H      1.784020   -2.147444    1.852436
 H     -0.723767   -3.635422   -2.140820
 H      2.272903   -4.342556    0.842975
 H      1.016548   -5.098677   -1.156711
 C      1.284889    1.043926    0.398768
 C      1.997888    0.655620   -0.736380
 C      1.535105    2.301707    0.954869
 C      2.933740    1.511375   -1.306853
 C      2.460473    3.160930    0.377164
 C      3.163911    2.765755   -0.755296
 H      1.824290   -0.318344   -1.179972
 H      0.997724    2.611560    1.846085
 H      3.482571    1.195840   -2.187444
 H      2.639037    4.135816    0.817168
 H      3.893018    3.431994   -1.202963
```

2

```
charge, 2S+1    = 0, 1
--- OPT at ωB97X-D/6-311G(d,p) in SMD ---
E(ele)          = -344.51410764 a.u.
Correction to G = 0.067971 a.u.(RRHO)
Correction to G = 0.068389 a.u.(quasi-RRHO)
--- SP at ωB97X-D/def2-TZVP in SMD ---
E(ele)          = -344.56117406 a.u.
 C      1.836607   -0.288395    0.000577
 C      2.647212   -1.299558    0.006041
 H      2.992206   -1.731624    0.940126
 H      2.991662   -1.742238   -0.923267
 C      1.031202    0.741477   -0.004997
 H      1.422363    1.752953   -0.011100
 C     -0.446041    0.636843   -0.003503
 O     -1.174098    1.597426   -0.007445
 O     -0.878555   -0.626752    0.003454
 C     -2.297319   -0.797105    0.005738
 H     -2.462713   -1.872709    0.010708
 H     -2.740337   -0.343516    0.894506
 H     -2.742248   -0.351409   -0.886063
```

e-iii

```
charge, 2S+1    = 0, 1
--- OPT at ωB97X-D/6-311G(d,p) in SMD ---
E(ele)          = -860.79914404 a.u.
Correction to G = 0.215517 a.u.(RRHO)
Correction to G = 0.218139 a.u.(quasi-RRHO)
--- SP at ωB97X-D/def2-TZVP in SMD ---
E(ele)          = -860.90447065 a.u.
 C      1.210822    1.414754    0.022858
 O      1.320365    2.614909    0.008129
 C     -0.056818    0.625756    0.048177
 C     -1.221060    1.291728    0.041687
 H     -1.131167    2.374925    0.104558
 N      2.271951    0.481432    0.000888
 C      1.738756   -0.837897    0.071218
 C      0.335963   -0.780984    0.103504
 C      2.414031   -2.047266    0.138573
 C      1.656757   -3.210025    0.258352
 C      0.269503   -3.168008    0.322594
 C     -0.398496   -1.951680    0.247016
 H      3.490428   -2.082381    0.105410
 H      2.169792   -4.163568    0.311234
 H     -0.296414   -4.085578    0.432984
 H     -1.478030   -1.920028    0.308174
 C      3.646018    0.783673   -0.075511
 O      4.460036   -0.107880   -0.105547
 C      4.049397    2.230121   -0.118841
 H      3.706713    2.762068    0.769413
 H      5.136234    2.254538   -0.178286
 H      3.611753    2.737158   -0.979548
 C     -2.588190    0.763824   -0.039016
 C     -3.585663    1.323675    0.765530
 C     -4.889962    0.854719    0.700607
 C     -5.222895   -0.157599   -0.192563
 C     -4.245987   -0.696016   -1.022806
 C     -2.937142   -0.240826   -0.946265
 H     -3.329774    2.123154    1.452926
 H     -5.650079    1.286386    1.341765
 H     -6.243931   -0.517398   -0.250349
 H     -4.504736   -1.469727   -1.736754
 H     -2.182000   -0.649889   -1.607859
```

7-Eg

```
charge, 2S+1    = 0, 1
--- OPT at ωB97X-D/6-311G(d,p) in SMD ---
E(ele)          = -1205.39961413 a.u.
Correction to G = 0.313748 a.u.(RRHO)
Correction to G = 0.318699 a.u.(quasi-RRHO)
--- SP at ωB97X-D/def2-TZVP in SMD ---
E(ele)          = -1205.54649990 a.u.
 C     1.258473   -2.927613    0.565773
 C    -0.205551   -3.022628    0.869946
 H    -0.426921   -3.675002    1.717981
 H    -0.734575   -3.438884    0.005277
 C     1.634533   -1.675984    0.305521
 H     1.927847   -3.778452    0.526311
 C     3.004753   -1.288769   -0.091341
 O     3.945180   -2.038071   -0.161922
 O     3.068690    0.019315   -0.367431
 C     4.342381    0.521635   -0.776242
 H     5.080709    0.383694    0.015653
 H     4.680561    0.015099   -1.681855
 H     4.190430    1.581093   -0.972005
 C     0.707817    0.602011    1.058852
 O     1.114056    0.712929    2.184753
 C     0.458892   -0.722025    0.337093
 C    -0.586734   -1.560078    1.161792
 H    -0.308872   -1.375755    2.203893
 N     0.314618    1.649877    0.205801
 C    -0.086281    1.116416   -1.055023
 C     0.020064   -0.271285   -1.025105
 C    -0.509917    1.776586   -2.199121
 C    -0.811233    1.003580   -3.319164
 C    -0.683215   -0.379385   -3.305688
 C    -0.259112   -1.025381   -2.147978
 H    -0.598381    2.850324   -2.221065
 H    -1.144791    1.503288   -4.221628
 H    -0.911221   -0.954286   -4.195686
 H    -0.142948   -2.103611   -2.123496
 C     0.337202    3.023728    0.514152
 O    -0.019180    3.832204   -0.309146
 C     0.811269    3.434921    1.878952
 H     1.833626    3.101219    2.059933
 H     0.754937    4.521415    1.920542
 H     0.191564    2.995014    2.661228
 C    -2.020180   -1.127764    0.980749
 C    -2.543990   -0.164976    1.845445
 C    -3.835138    0.319775    1.680010
 C    -4.628786   -0.157452    0.644529
 C    -4.121654   -1.120326   -0.219457
 C    -2.828633   -1.599599   -0.053242
 H    -1.929746    0.208012    2.659579
 H    -4.221570    1.068283    2.362791
 H    -5.637312    0.217790    0.512068
 H    -4.733350   -1.497767   -1.031307
 H    -2.449093   -2.339886   -0.747756
```

ts3-Z

```
charge, 2S+1    = 0, 1
--- OPT at ωB97X-D/6-311G(d,p) in SMD ---
E(ele)          = -1380.73844658 a.u.
Correction to G = 0.318826 a.u.(RRHO)
Correction to G = 0.325562 a.u.(quasi-RRHO)
imaginary frequency = 265i
--- SP at ωB97X-D/def2-TZVP in SMD ---
E(ele)          = -1380.87180935 a.u.
 P     0.183745   -0.445019   -0.267726
 C    -1.390786   -0.911086    0.518997
 C    -1.354331   -1.821343    1.574818
 C    -2.617449   -0.399748    0.088591
 C    -2.534792   -2.230296    2.182336
 C    -3.793921   -0.806335    0.703156
 C    -3.753412   -1.724348    1.747367
 H    -0.401680   -2.191711    1.935953
 H    -2.651785    0.319152   -0.722792
 H    -2.499722   -2.933286    3.006706
 H    -4.743323   -0.404189    0.367877
 H    -4.673196   -2.037941    2.228504
 C     0.291495   -1.462760   -1.780416
 C     1.510595   -1.474014   -2.467068
 C    -0.763743   -2.244478   -2.251943
 C     1.659724   -2.231214   -3.620526
 C    -0.605902   -3.011454   -3.400328
 C     0.600998   -3.002406   -4.088259
 H     2.346043   -0.890691   -2.093238
 H    -1.709623   -2.259001   -1.723053
 H     2.606663   -2.227477   -4.148237
 H    -1.431258   -3.616961   -3.757558
 H     0.719954   -3.601010   -4.984384
 C    -0.052027    1.266680   -0.828974
 C    -0.339802    2.229607    0.144117
 C     0.123103    1.664388   -2.155571
 C    -0.455929    3.566633   -0.210545
 C     0.007662    3.004607   -2.502693
 C    -0.279053    3.957482   -1.532455
 H    -0.489487    1.927452    1.175201
 H     0.338850    0.930935   -2.923824
 H    -0.681442    4.304220    0.551268
 H     0.138437    3.302935   -3.536865
 H    -0.366049    5.002849   -1.806908
 C     2.094197   -0.536227    1.069992
 C     2.465862   -1.797578    0.973292
 H     2.004499   -2.518624    0.309223
 H     3.288727   -2.139422    1.595722
 C     2.421877    0.678135    1.602110
 H     3.227375    1.257889    1.168417
 C     1.583235    1.277817    2.595790
 O     0.540749    0.816692    3.036920
 O     2.059375    2.479668    3.010788
 C     1.271797    3.148668    3.985063
 H     1.147330    2.540201    4.884000
 H     1.813161    4.062717    4.227781
 H     0.282173    3.399681    3.593060
```

3-Z

```
charge, 2S+1    = 0, 1
--- OPT at ωB97X-D/6-311G(d,p) in SMD ---
E(ele)          = -1380.76793070 a.u.
Correction to G = 0.324112 a.u.(RRHO)
Correction to G = 0.329136 a.u.(quasi-RRHO)
--- SP at ωB97X-D/def2-TZVP in SMD ---
E(ele)          = -1380.90552893 a.u.
 P     0.210491   -0.280199   -0.162930
 C    -1.267316   -0.797443    0.750437
 C    -1.163866   -1.237231    2.068462
 C    -2.498541   -0.830525    0.094966
 C    -2.293689   -1.700986    2.726794
 C    -3.629387   -1.272679    0.768978
 C    -3.526451   -1.709582    2.083686
 H    -0.205331   -1.217934    2.572913
 H    -2.578477   -0.528114   -0.943601
 H    -2.210842   -2.048739    3.749922
 H    -4.586520   -1.286625    0.260776
 H    -4.407698   -2.063632    2.606842
 C     0.086144   -1.288666   -1.684369
 C     0.347621   -0.749505   -2.942900
 C    -0.269581   -2.636968   -1.574984
 C     0.259214   -1.549309   -4.076604
 C    -0.350793   -3.431863   -2.708096
 C    -0.086913   -2.888513   -3.961152
 H     0.616590    0.294304   -3.048018
 H    -0.479567   -3.067915   -0.602641
 H     0.461142   -1.119915   -5.051079
 H    -0.623090   -4.476558   -2.612482
 H    -0.154398   -3.510217   -4.846850
 C     0.212208    1.433302   -0.745882
 C    -0.960954    2.172834   -0.866625
 C     1.435939    1.988071   -1.116348
 C    -0.908808    3.464682   -1.369350
 C     1.478900    3.283805   -1.616315
 C     0.308898    4.020553   -1.746693
 H    -1.908725    1.759720   -0.543819
 H     2.351457    1.417704   -1.004322
 H    -1.821443    4.043140   -1.455187
 H     2.431748    3.717752   -1.896554
 H     0.345659    5.032647   -2.133664
 C     1.793080   -0.691409    0.654251
 C     2.530242   -1.655621    0.044655
 H     2.228180   -2.163295   -0.858475
 H     3.487904   -1.934665    0.473232
 C     2.163655    0.057397    1.805511
 H     3.148010   -0.104497    2.225882
 C     1.334954    1.042124    2.353746
 O     0.188062    1.319124    1.967092
 O     1.884721    1.719822    3.399793
 C     1.066416    2.726634    3.971825
 H     0.135015    2.311676    4.367036
 H     1.650643    3.157781    4.785280
 H     0.818888    3.503882    3.243663
```

ts3-E

```
charge, 2S+1    = 0, 1
--- OPT at ωB97X-D/6-311G(d,p) in SMD ---
E(ele)          = -1380.73651595 a.u.
Correction to G = 0.319936 a.u.(RRHO)
Correction to G = 0.326102 a.u.(quasi-RRHO)
imaginary frequency = 257i
--- SP at ωB97X-D/def2-TZVP in SMD ---
E(ele)          = -1380.87034658 a.u.
 P    -0.770317    0.041980    0.303238
 C    -2.073867    1.318256    0.355999
 C    -3.106051    1.164008    1.289090
 C    -2.024517    2.476339   -0.420995
 C    -4.075968    2.145570    1.430224
 C    -2.996851    3.458545   -0.271848
 C    -4.022309    3.296729    0.650848
 H    -3.158525    0.266691    1.897946
 H    -1.232959    2.611406   -1.149020
 H    -4.874368    2.012297    2.151475
 H    -2.952307    4.352121   -0.884224
 H    -4.779230    4.064676    0.763307
 C    -1.679075   -1.506279   -0.028182
 C    -2.849440   -1.525707   -0.792553
 C    -1.178923   -2.703523    0.486268
 C    -3.500987   -2.725571   -1.043085
 C    -1.831836   -3.903068    0.229950
 C    -2.991536   -3.915547   -0.534463
 H    -3.254743   -0.601048   -1.188507
 H    -0.282398   -2.702539    1.095579
 H    -4.408725   -2.730882   -1.635852
 H    -1.435459   -4.827130    0.634763
 H    -3.502455   -4.851653   -0.730221
 C     0.213339    0.381641   -1.185276
 C     1.259988    1.298792   -1.059446
 C    -0.016137   -0.238324   -2.413926
 C     2.049571    1.609337   -2.158915
 C     0.779072    0.073382   -3.509202
 C     1.808916    0.998851   -3.383921
 H     1.476369    1.753776   -0.098540
 H    -0.810406   -0.968533   -2.519057
 H     2.861310    2.319411   -2.049901
 H     0.595808   -0.411681   -4.461344
 H     2.430433    1.236651   -4.240138
 C     0.757083   -0.290500    1.983657
 C     0.177487    0.205827    3.058491
 H    -0.767093    0.735878    3.045136
 H     0.682655    0.090026    4.013679
 C     1.910343   -0.846475    1.505036
 H     1.934660   -1.869508    1.149259
 C     3.076495   -0.030858    1.288713
 O     3.207426    1.158471    1.519748
 O     4.096014   -0.768517    0.771946
 C     5.296831   -0.055999    0.515117
 H     5.140511    0.727141   -0.231663
 H     5.691930    0.401468    1.425530
 H     6.003862   -0.792468    0.133607
```

3-E

```
charge, 2S+1    = 0, 1
--- OPT at ωB97X-D/6-311G(d,p) in SMD ---
E(ele)          = -1380.76345971 a.u.
Correction to G = 0.322654 a.u.(RRHO)
Correction to G = 0.328394 a.u.(quasi-RRHO)
--- SP at ωB97X-D/def2-TZVP in SMD ---
E(ele)          = -1380.90207260 a.u.
 P    -0.279724    0.209411   -0.196294
 C    -1.523436   -0.972934    0.389977
 C    -2.725263   -1.110082   -0.312067
 C    -1.287454   -1.743959    1.528260
 C    -3.676265   -2.024419    0.116959
 C    -2.249397   -2.651294    1.953621
 C    -3.437835   -2.795749    1.249312
 H    -2.927041   -0.496740   -1.183444
 H    -0.356309   -1.630295    2.072706
```

H	-4.606681	-2.128221	-0.428960
H	-2.065411	-3.244943	2.841301
H	-4.184012	-3.506527	1.586104
C	0.598524	-0.494942	-1.614337
C	0.020908	-1.474898	-2.420234
C	1.867958	0.004228	-1.916071
C	0.708094	-1.946069	-3.531180
C	2.545579	-0.469473	-3.030439
C	1.966144	-1.441924	-3.837646
H	-0.953548	-1.883620	-2.180331
H	2.327929	0.745905	-1.273063
H	0.261524	-2.713237	-4.152785
H	3.532788	-0.086246	-3.259865
H	2.501685	-1.815091	-4.703281
C	-1.196560	1.658145	-0.780447
C	-0.864096	2.277857	-1.983950
C	-2.230001	2.171291	0.008907
C	-1.560129	3.408347	-2.393241
C	-2.918076	3.301559	-0.405871
C	-2.583499	3.919920	-1.606087
H	-0.068906	1.882275	-2.604815
H	-2.493217	1.691235	0.944772
H	-1.300106	3.888125	-3.329437
H	-3.716290	3.699646	0.209373
H	-3.124227	4.802667	-1.928008
C	0.908631	0.618838	1.098819
C	0.774663	1.852364	1.659284
H	0.057482	2.576298	1.297273
H	1.418928	2.123535	2.484594
C	1.782510	-0.462972	1.392466
H	1.780319	-1.350557	0.773906
C	2.710333	-0.413981	2.460294
O	2.906517	0.498965	3.254845
O	3.441862	-1.578460	2.549574
C	4.398234	-1.611894	3.591300
H	4.884840	-2.585862	3.520954
H	5.145416	-0.820312	3.480577
H	3.930093	-1.506410	4.574698

ts3-int

charge, 2S+1 = 0, 1
--- OPT at ωB97X-D/6-311G(d,p) in SMD ---
E(ele) = -1380.74208854 a.u.
Correction to G = 0.320260 a.u.(RRHO)
Correction to G = 0.326242 a.u.(quasi-RRHO)
imaginary frequency = 258i
--- SP at ωB97X-D/def2-TZVP in SMD ---
E(ele) = -1380.87428440 a.u.

P	-0.515728	0.048230	0.398927
C	-2.038826	-0.956813	0.494917
C	-2.731650	-1.017144	1.704442
C	-2.529093	-1.656198	-0.611646
C	-3.907760	-1.751375	1.803227
C	-3.701617	-2.391742	-0.508451
C	-4.393620	-2.437381	0.697549
H	-2.345513	-0.498883	2.575438
H	-1.990520	-1.627537	-1.552440
H	-4.438660	-1.793321	2.747423
H	-4.075054	-2.932132	-1.371002
H	-5.308436	-3.014131	0.775713
C	0.530289	-0.844563	-0.783474
C	1.052832	-2.069180	-0.360361
C	0.894215	-0.335230	-2.029988
C	1.929325	-2.774096	-1.175505
C	1.764039	-1.046902	-2.844025
C	2.286522	-2.263546	-2.416765
H	0.777780	-2.472579	0.607942
H	0.507148	0.620746	-2.362426
H	2.337132	-3.718843	-0.835067
H	2.039193	-0.647219	-3.813594
H	2.972858	-2.812310	-3.052194
C	-0.991382	1.618104	-0.383726
C	-0.003469	2.607401	-0.454399
C	-2.280075	1.890063	-0.845141
C	-0.302986	3.843207	-1.011406
C	-2.573524	3.134709	-1.389206
C	-1.586401	4.109117	-1.477126
H	0.994187	2.393841	-0.079413
H	-3.055252	1.134624	-0.782630
H	0.467335	4.603661	-1.072597
H	-3.576004	3.341184	-1.747070
H	-1.818798	5.078605	-1.903827
C	0.771455	0.349571	2.311851
C	0.039306	1.156388	3.053855
H	-0.972079	1.454917	2.806118
H	0.482708	1.572937	3.954451
C	1.961029	-0.313400	2.215941
H	2.117626	-1.247528	2.741013
C	2.900640	0.100147	1.212586
O	2.789443	1.074791	0.483411
O	3.967309	-0.730660	1.127864
C	4.892125	-0.430503	0.091230
H	5.348790	0.551628	0.237428
H	5.656963	-1.205112	0.142564
H	4.404045	-0.452001	-0.886361

3-int

charge, 2S+1 = 0, 1
--- OPT at ωB97X-D/6-311G(d,p) in SMD ---
E(ele) = -1380.75926783 a.u.
Correction to G = 0.323649 a.u.(RRHO)
Correction to G = 0.328890 a.u.(quasi-RRHO)
--- SP at ωB97X-D/def2-TZVP in SMD ---
E(ele) = -1380.89562239 a.u.

P	-0.365968	0.112298	0.336345
C	-1.894357	-0.874007	0.523558
C	-2.296743	-1.435377	1.732603
C	-2.613050	-1.161992	-0.642607
C	-3.421593	-2.252552	1.778772
C	-3.738390	-1.968312	-0.588716
C	-4.145819	-2.513920	0.624847
H	-1.733906	-1.239862	2.636646
H	-2.289798	-0.760835	-1.597220
H	-3.726161	-2.686571	2.724021
H	-4.291675	-2.179575	-1.496366
H	-5.022670	-3.150169	0.665713
C	0.576189	-0.920421	-0.814343
C	1.014206	-2.164046	-0.360485
C	0.790618	-0.538197	-2.135269
C	1.697954	-3.007275	-1.225108
C	1.461284	-1.394650	-2.997244
C	1.922268	-2.624186	-2.542060
H	0.840241	-2.465598	0.665406
H	0.453920	0.427714	-2.491525
H	2.053616	-3.966442	-0.867237
H	1.632500	-1.094498	-4.024601
H	2.453954	-3.286656	-3.215903
C	-0.813941	1.694768	-0.425247
C	0.217417	2.562061	-0.801145
C	-2.145504	2.089252	-0.558012
C	-0.095818	3.803331	-1.335813
C	-2.445322	3.337658	-1.088823
C	-1.423161	4.191065	-1.483178
H	1.247384	2.252664	-0.655050
H	-2.950735	1.435484	-0.246166
H	0.703420	4.473689	-1.630034
H	-3.480668	3.641992	-1.189524
H	-1.660823	5.164233	-1.898156
C	0.410812	0.384362	1.976238
C	-0.438043	0.912992	2.885683
H	-1.480355	1.125944	2.676234
H	-0.078442	1.146707	3.882481
C	1.753538	-0.061568	2.167767
H	2.057057	-0.395035	3.151694
C	2.726495	0.193734	1.190246
O	2.551945	0.796537	0.120462
O	3.963446	-0.296066	1.499236
C	4.971447	-0.059237	0.532627
H	5.111846	1.009658	0.348335
H	5.887082	-0.482856	0.947136
H	4.737789	-0.547765	-0.417815

ts3-rotZ

charge, 2S+1 = 0, 1
--- OPT at ωB97X-D/6-311G(d,p) in SMD ---
E(ele) = -1380.75501475 a.u.
Correction to G = 0.325463 a.u.(RRHO)
Correction to G = 0.329629 a.u.(quasi-RRHO)
imaginary frequency = 81i
--- SP at ωB97X-D/def2-TZVP in SMD ---
E(ele) = -1380.89122367 a.u.

P	0.396596	0.031159	-0.390244
C	1.879995	-1.048930	-0.418383
C	2.941570	-0.807107	-1.296212
C	1.996605	-2.089481	0.506176
C	4.079198	-1.598563	-1.261291
C	3.138074	-2.882385	0.535429
C	4.179515	-2.642104	-0.348207
H	2.897086	0.001423	-2.010974
H	1.200936	-2.291219	1.210705
H	4.889646	-1.397184	-1.952038
H	3.206802	-3.689096	1.255948
H	5.068745	-3.261760	-0.325645
C	-0.707802	-0.759402	0.803428
C	-1.320672	-1.968108	0.469153
C	-0.913233	-0.190707	2.057510
C	-2.158589	-2.587550	1.386457
C	-1.743279	-0.822912	2.972176
C	-2.371274	-2.016348	2.635459
H	-1.161122	-2.411237	-0.505511
H	-0.448122	0.753359	2.314691
H	-2.647488	-3.517500	1.120626
H	-1.909148	-0.374323	3.944739
H	-3.027762	-2.502618	3.348455
C	0.947155	1.630044	0.254037
C	0.100473	2.730523	0.096166
C	2.169060	1.767283	0.913904
C	0.488625	3.965164	0.596701
C	2.546254	3.008327	1.410392
C	1.708413	4.105371	1.249781
H	-0.861431	2.594889	-0.386633
H	2.827001	0.915237	1.039257
H	-0.166819	4.819948	0.477044
H	3.496085	3.116701	1.921035
H	2.006878	5.073538	1.636382
C	-0.362273	0.212591	-2.066314
C	0.453428	0.682876	-3.029161
H	1.490157	0.948546	-2.876118
H	0.056803	0.832035	-4.028229
C	-1.715455	-0.239169	-2.212038
H	-2.008713	-0.744487	-3.122704
C	-2.685686	0.244489	-1.324460
O	-2.491442	1.049786	-0.400745
O	-3.939796	-0.258955	-1.537228
C	-4.940026	0.209587	-0.651572
H	-4.723509	-0.072488	0.383035
H	-5.046216	1.297281	-0.698568
H	-5.869104	-0.262982	-0.973479

ts3-rotE

charge, 2S+1 = 0, 1
--- OPT at ωB97X-D/6-311G(d,p) in SMD ---
E(ele) = -1380.74613644 a.u.
Correction to G = 0.323575 a.u.(RRHO)
Correction to G = 0.328471 a.u.(quasi-RRHO)
imaginary frequency = 82i
--- SP at ωB97X-D/def2-TZVP in SMD ---
E(ele) = -1380.88327539 a.u.

P	-0.521304	0.024169	0.445139
C	-2.022745	-0.983222	0.595327
C	-2.652802	-1.118768	1.831575
C	-2.559980	-1.609446	-0.534141
C	-3.819834	-1.866085	1.934316
C	-3.725965	-2.352413	-0.423337
C	-4.357063	-2.478927	0.810010
H	-2.228426	-0.655433	2.714388
H	-2.065179	-1.523249	-1.495263
H	-4.304208	-1.973150	2.897746
H	-4.139583	-2.837138	-1.299951
H	-5.266446	-3.063008	0.894145
C	0.584903	-0.851614	-0.683514
C	0.766591	-2.225578	-0.510611
C	1.324598	-0.164210	-1.642157
C	1.680675	-2.905370	-1.299843
C	2.240970	-0.852336	-2.427806
C	2.419181	-2.218357	-2.257686
H	0.200408	-2.763286	0.242027
H	1.199782	0.903349	-1.774936
H	1.823106	-3.970637	-1.161359
H	2.818707	-0.314802	-3.170433
H	3.138481	-2.750730	-2.869667
C	-0.960494	1.622820	-0.287873

C -0.029632 2.662671 -0.180808
C -2.169961 1.820180 -0.954457
C -0.317740 3.890803 -0.761117
C -2.448099 3.057572 -1.520271
C -1.522041 4.089754 -1.427201
H 0.908418 2.507198 0.355056
H -2.899997 1.022350 -1.025624
H 0.402269 4.697169 -0.682558
H -3.390962 3.214493 -2.031358
H -1.742803 5.054615 -1.870085
C 0.386775 0.312079 2.036722
C -0.220151 1.159938 2.871481
H -1.170999 1.640178 2.652571
H 0.272430 1.427419 3.800697
C 1.736750 -0.208716 2.119895
H 1.904486 -1.278929 2.112508
C 2.765507 0.617257 1.644800
O 2.698919 1.800310 1.279075
O 3.979358 -0.036100 1.581038
C 5.045977 0.719124 1.047979
H 5.244872 1.619899 1.636483
H 5.920083 0.065722 1.076540
H 4.853154 1.022464 0.013406

ts3-rot

charge, 2S+1 = 0, 1
--- OPT at ωB97X-D/6-311G(d,p) in SMD ---
E(ele) = -1380.74630614 a.u.
Correction to G = 0.322267 a.u.(RRHO)
Correction to G = 0.327793 a.u.(quasi-RRHO)
imaginary frequency = 129i
--- SP at ωB97X-D/def2-TZVP in SMD ---
E(ele) = -1380.88400463 a.u.

P -0.356037 -0.346071 0.194733
C -1.883247 -1.325562 0.258184
C -3.132801 -0.758196 0.012902
C -1.788844 -2.680597 0.594929
C -4.276742 -1.543939 0.089415
C -2.933976 -3.459326 0.662975
C -4.178671 -2.891461 0.409330
H -3.219195 0.293979 -0.230809
H -0.822424 -3.122528 0.812812
H -5.246101 -1.097571 -0.099372
H -2.855371 -4.508694 0.921990
H -5.073334 -3.500828 0.468146
C 0.610062 -0.877043 -1.235327
C 0.253098 -1.980902 -2.009220
C 1.759603 -0.143309 -1.547164
C 1.050223 -2.358211 -3.082485
C 2.549786 -0.529910 -2.620086
C 2.199316 -1.637586 -3.383448
H -0.644260 -2.545219 -1.786139
H 2.028283 0.728692 -0.964142
H 0.770226 -3.215818 -3.683071
H 3.442465 0.036917 -2.856219
H 2.820995 -1.936081 -4.220166
C -0.813539 1.378214 -0.097724
C -0.805343 2.294174 0.950863
C -1.224143 1.768480 -1.376137
C -1.228720 3.597498 0.720728
C -1.645699 3.070877 -1.595030
C -1.652066 3.983316 -0.543915
H -0.416131 2.007800 1.918670
H -1.208206 1.060168 -2.197770
H -1.207941 4.315625 1.531831
H -1.961386 3.375982 -2.585936
H -1.975267 5.003679 -0.717789
C 0.649659 -0.540055 1.746208
C -0.069622 -0.808996 2.836866
H -1.148952 -0.934403 2.832101
H 0.440829 -0.905721 3.790222
C 2.089011 -0.372546 1.631771
H 2.697170 -1.234631 1.389050
C 2.650700 0.904065 1.612315
O 2.083955 1.996675 1.744946
O 4.020225 0.875561 1.397891
C 4.660149 2.133245 1.433157
H 4.277651 2.810668 0.662987
H 4.544029 2.624979 2.404511
H 5.718756 1.937202 1.251622

ts4-Za

charge, 2S+1 = 0, 1
--- OPT at ωB97X-D/6-311G(d,p) in SMD ---
E(ele) = -2241.57826754 a.u.
Correction to G = 0.567509 a.u.(RRHO)
Correction to G = 0.576897 a.u.(quasi-RRHO)
imaginary frequency = 311i
--- SP at ωB97X-D/def2-TZVP in SMD ---
E(ele) = -2241.81442672 a.u.

P -1.968420 -0.467394 0.075064
C -2.132617 -2.262062 -0.210304
C -2.514969 -3.064253 0.870526
C -1.846197 -2.856539 -1.438226
C -2.596335 -4.440531 0.723746
C -1.933240 -4.236317 -1.579297
C -2.303044 -5.028935 -0.501353
H -2.744250 -2.615780 1.830284
H -1.555870 -2.260323 -2.291667
H -2.888766 -5.052999 1.568527
H -1.707959 -4.688328 -2.538070
H -2.366274 -6.105086 -0.615465
C -2.143736 0.377409 -1.518949
C -3.213267 1.239870 -1.758861
C -1.177766 0.169191 -2.504026
C -3.324399 1.872520 -2.987561
C -1.292858 0.817088 -3.728401
C -2.366066 1.662878 -3.973470
H -3.935943 1.448142 -0.981315
H -0.318173 -0.471071 -2.323072
H -4.154056 2.546050 -3.168186
H -0.531936 0.660608 -4.484049
H -2.452154 2.168092 -4.928932
C -3.359911 -0.097727 1.174929
C -3.148123 0.430700 2.445323
C -4.650460 -0.435437 0.762538
C -4.227866 0.634303 3.294264
C -5.725976 -0.217650 1.611293
C -5.514626 0.317898 2.876897
H -2.146617 0.678649 2.775134
H -4.817342 -0.875766 -0.214873
H -4.060931 1.044585 4.283233
H -6.727502 -0.474373 1.286650
H -6.355085 0.482846 3.541494
C -0.379975 -0.184886 0.915343
C 0.311556 -1.287283 1.287253
H -0.006720 -2.296988 1.071722
H 1.217512 -1.172086 1.871001
C 0.159287 1.137762 1.080376
H 0.993417 1.203613 1.769236
C -0.671775 2.308275 1.037041
O -1.791553 2.382842 0.546649
O -0.071065 3.378630 1.591250
C -0.784630 4.606471 1.512525
H -1.686091 4.573718 2.130194
H -0.103100 5.368680 1.886981
H -1.068479 4.833259 0.483497
C 2.214125 -1.193680 -0.977821
O 1.484670 -1.447986 -1.928491
C 2.444802 0.067313 -0.312033
C 1.664532 1.169250 -0.646057
H 0.915301 0.935190 -1.393095
N 3.090150 -2.154063 -0.366990
C 3.805900 -1.522263 0.677650
C 3.411653 -0.169389 0.738837
C 4.696340 -2.061843 1.592886
C 5.177962 -1.235479 2.608040
C 4.758582 0.084932 2.716406
C 3.870262 0.622279 1.789822
H 4.996357 -3.095102 1.522685
H 5.878617 -1.641895 3.328837
H 5.122326 0.703341 3.529658
H 3.532926 1.646469 1.893151
C 3.181271 -3.512144 -0.678337
O 3.994117 -4.219690 -0.123410
C 2.231532 -4.072610 -1.702926
H 2.366774 -3.589773 -2.670545
H 2.436047 -5.140204 -1.774015
H 1.193809 -3.901636 -1.411239
C 2.045341 2.594214 -0.695255
C 3.327635 3.062144 -0.399306
C 3.641141 4.408994 -0.534285
C 2.686832 5.312264 -0.984942
C 1.416968 4.853730 -1.321339
C 1.104823 3.511335 -1.182268
H 4.099158 2.367587 -0.094205
H 4.643654 4.749496 -0.299288
H 2.935553 6.362335 -1.091873
H 0.670182 5.543971 -1.698499
H 0.112148 3.158226 -1.445796

ts4-Zg

charge, 2S+1 = 0, 1
--- OPT at ωB97X-D/6-311G(d,p) in SMD ---
E(ele) = -2241.57810643 a.u.
Correction to G = 0.568007 a.u.(RRHO)
Correction to G = 0.576975 a.u.(quasi-RRHO)
imaginary frequency = 374i
--- SP at ωB97X-D/def2-TZVP in SMD ---
E(ele) = -2241.81460049 a.u.

P -2.084189 -0.085506 0.117202
C -2.318186 1.013964 -1.300511
C -3.550347 1.620761 -1.534675
C -1.257497 1.209187 -2.180296
C -3.718726 2.416398 -2.658810
C -1.434683 2.011116 -3.300350
C -2.663484 2.611603 -3.542967
H -4.369754 1.502684 -0.836594
H -0.280491 0.774466 -2.000427
H -4.674588 2.895575 -2.836339
H -0.600118 2.167416 -3.973746
H -2.798208 3.240430 -4.415888
C -3.318087 0.224874 1.404661
C -4.644942 -0.158285 1.189730
C -2.950047 0.824934 2.607125
C -5.598838 0.075505 2.169666
C -3.909217 1.046374 3.585921
C -5.230776 0.678103 3.367231
H -4.934045 -0.649564 0.267163
H -1.921583 1.115262 2.779108
H -6.627447 -0.220076 1.999579
H -3.620263 1.510906 4.521240
H -5.976101 0.855581 4.134239
C -2.436151 -1.762693 -0.502773
C -2.297265 -2.059024 -1.858588
C -2.797870 -2.773890 0.392510
C -2.508406 -3.355692 -2.311891
C -3.004102 -4.066618 -0.066045
C -2.858322 -4.359003 -1.418127
H -2.027080 -1.285665 -2.567048
H -2.911317 -2.555272 1.448177
H -2.396003 -3.577767 -3.366449
H -3.278678 -4.846287 0.634566
H -3.020484 -5.369985 -1.774281
C -0.410495 -0.099123 0.832779
C 0.162453 -1.357563 1.006989
H -0.402720 -2.263725 0.862991
H 0.983660 -1.430353 1.711372
C 0.287464 1.070741 1.100209
H 1.264378 0.979320 1.554385
C -0.220480 2.401856 0.923729
O -1.381196 2.712330 0.700135
O 0.753120 3.328072 1.051115
C 0.356576 4.684497 0.885501
H 1.270910 5.269202 0.969970
H -0.103157 4.842930 -0.092591
H -0.352502 4.982511 1.661840
C 2.370437 0.802780 -1.160389
O 1.769086 0.964829 -2.211327
C 2.509094 -0.411363 -0.378959
C 1.705659 -1.502999 -0.660351
H 1.015963 -1.325389 -1.482511
N 3.175487 1.803001 -0.531101
C 3.812953 1.235047 0.595297
C 3.415830 -0.113340 0.712002
C 4.642266 1.830986 1.532081
C 5.064338 1.064732 2.617745
C 4.651701 -0.253560 2.770452
C 3.822721 -0.847132 1.823773
H 4.938352 2.862009 1.423358
H 5.716786 1.515808 3.357026
H 4.975788 -0.825796 3.632682
H 3.497922 -1.872187 1.954113

C	3.202615	3.162018	-0.853694
O	3.958908	3.918149	-0.284243
C	2.258548	3.653766	-1.917844
H	2.334256	4.740439	-1.932033
H	2.529257	3.244991	-2.892009
H	1.233814	3.340892	-1.717878
C	2.024662	-2.938198	-0.474971
C	3.324672	-3.382832	-0.223900
C	3.605771	-4.740442	-0.119597
C	2.597475	-5.681537	-0.280058
C	1.304029	-5.254085	-0.566115
C	1.025145	-3.900844	-0.667472
H	4.129619	-2.663630	-0.137176
H	4.623232	-5.060939	0.075799
H	2.818784	-6.739871	-0.198491
H	0.510241	-5.978178	-0.715066
H	0.015591	-3.578548	-0.900794

ts4-Ea

charge, 2S+1 = 0, 1
--- OPT at ωB97X-D/6-311G(d,p) in SMD ---
E(ele) = -2241.57956048 a.u.
Correction to G = 0.567576 a.u.(RRHO)
Correction to G = 0.576844 a.u.(quasi-RRHO)
imaginary frequency = 323i
--- SP at ωB97X-D/def2-TZVP in SMD ---
E(ele) = -2241.81522058 a.u.

P	-2.017599	0.682107	0.063381
C	-1.921888	2.483935	-0.093873
C	-2.055043	3.045804	-1.367807
C	-1.751425	3.310121	1.015740
C	-1.991958	4.422088	-1.526891
C	-1.705564	4.688135	0.849036
C	-1.818331	5.243626	-0.418499
H	-2.220338	2.412985	-2.232696
H	-1.654620	2.892062	2.008597
H	-2.086435	4.852027	-2.516888
H	-1.572363	5.325902	1.714695
H	-1.773260	6.319217	-0.544776
C	-2.245784	0.214714	1.797064
C	-1.215098	0.469239	2.707681
C	-3.400969	-0.449292	2.209910
C	-1.354047	0.064081	4.027471
C	-3.527320	-0.850696	3.533830
C	-2.507073	-0.594680	4.440896
H	-0.305285	0.964934	2.377404
H	-4.195252	-0.666427	1.505945
H	-0.552656	0.257310	4.730916
H	-4.423122	-1.370890	3.852033
H	-2.607566	-0.914411	5.471945
C	-3.504563	0.189831	-0.848852
C	-4.682332	0.926071	-0.693511
C	-3.479238	-0.924034	-1.686015
C	-5.830169	0.539711	-1.368751
C	-4.633413	-1.302679	-2.360635
C	-5.805529	-0.575085	-2.201178
H	-4.702144	1.800520	-0.051748
H	-2.562451	-1.486209	-1.819642
H	-6.742810	1.111752	-1.249815
H	-4.611237	-2.165872	-3.015187
H	-6.702956	-0.872440	-2.731752
C	-0.612313	-0.195559	-0.641177
C	0.088721	0.397194	-1.632059
H	-0.118049	1.405189	-1.968676
H	0.846042	-0.164146	-2.160879
C	-0.357548	-1.490693	-0.068878
H	-0.991667	-1.807431	0.750285
C	0.089612	-2.589790	-0.905821
O	0.698685	-2.523668	-1.954686
O	-0.237212	-3.779023	-0.343192
C	0.408982	-4.921216	-0.891187
H	0.045884	-5.772424	-0.316535
H	0.162548	-5.049145	-1.947504
H	1.493797	-4.840106	-0.781899
C	2.009691	1.228265	0.425063
O	1.280026	1.741450	1.269750
C	2.266893	-0.169061	0.178925
C	1.595309	-1.099906	0.972452
H	1.021136	-0.623822	1.759055
N	2.830694	1.977236	-0.477136
C	3.565990	1.074018	-1.281911
C	3.200323	-0.241757	-0.934112
C	4.452659	1.340657	-2.315193
C	4.960476	0.264002	-3.038157
C	4.564065	-1.037533	-2.750209
C	3.679721	-1.295204	-1.709628
H	4.729882	2.355690	-2.550878
H	5.657334	0.453055	-3.847199
H	4.936582	-1.861475	-3.349117
H	3.330486	-2.302836	-1.532058
C	2.843126	3.364195	-0.631540
O	3.660432	3.902267	-1.347047
C	1.784670	4.160657	0.081670
H	0.790260	3.779671	-0.152943
H	1.897684	4.087848	1.163072
H	1.885936	5.194625	-0.246065
C	2.055643	-2.451720	1.373723
C	1.214434	-3.250483	2.158175
C	1.614879	-4.500754	2.600253
C	2.879105	-4.983732	2.277114
C	3.741127	-4.187967	1.534341
C	3.336625	-2.932883	1.094034
H	0.225379	-2.885599	2.411462
H	0.938275	-5.101732	3.198020
H	3.194605	-5.963838	2.617651
H	4.741639	-4.536713	1.302800
H	4.039490	-2.312246	0.555722

ts4-Eg

charge, 2S+1 = 0, 1
--- OPT at ωB97X-D/6-311G(d,p) in SMD ---
E(ele) = -2241.58624461 a.u.
Correction to G = 0.570369 a.u.(RRHO)
Correction to G = 0.578381 a.u.(quasi-RRHO)
imaginary frequency = 338i
--- SP at ωB97X-D/def2-TZVP in SMD ---
E(ele) = -2241.82174506 a.u.

P	-2.219940	0.056545	-0.066253
C	-2.607969	1.300905	1.189940
C	-3.670352	2.186630	1.010240
C	-1.799605	1.392588	2.327336
C	-3.928466	3.155987	1.971340
C	-2.068474	2.364018	3.280710
C	-3.130804	3.244257	3.104673
H	-4.288791	2.134418	0.122484
H	-0.962692	0.711193	2.449421
H	-4.751210	3.846492	1.827753
H	-1.440842	2.435012	4.161500
H	-3.333200	4.004685	3.850444
C	-2.533003	-1.605348	0.583439
C	-2.721318	-1.835761	1.944734
C	-2.617092	-2.667543	-0.322525
C	-2.985185	-3.122106	2.396367
C	-2.869885	-3.951007	0.138522
C	-3.055308	-4.178433	1.497591
H	-2.663487	-1.022715	2.656382
H	-2.494695	-2.494459	-1.385889
H	-3.130737	-3.296816	3.455807
H	-2.925052	-4.772213	-0.566125
H	-3.255254	-5.181902	1.855558
C	-3.401545	0.254958	-1.430492
C	-4.716601	-0.191837	-1.272719
C	-3.009537	0.871226	-2.617327
C	-5.633919	-0.010692	-2.296853
C	-3.933916	1.044348	-3.640269
C	-5.242420	0.607644	-3.480247
H	-5.023102	-0.683368	-0.355527
H	-1.986421	1.206352	-2.744094
H	-6.653105	-0.357837	-2.173769
H	-3.625658	1.518394	-4.564620
H	-5.960041	0.743401	-4.281480
C	-0.574287	0.325575	-0.737529
C	-0.130040	1.644936	-0.725403
H	-0.752458	2.435326	-0.320107
H	0.582703	1.928665	-1.483370
C	0.111028	-0.775477	-1.229779
H	-0.235861	-1.779460	-1.027220
C	1.265470	-0.676021	-2.089595
O	1.733150	0.326272	-2.605055
O	1.800819	-1.902180	-2.303659
C	2.982504	-1.932977	-3.095170
H	2.765679	-1.653204	-4.130072
H	3.740944	-1.259681	-2.693711
H	3.338641	-2.961404	-3.055208
C	1.452317	-0.794196	1.313328
O	0.415041	-0.775228	1.966062
C	2.145862	0.320824	0.699632
C	1.565803	1.577468	0.777903
H	0.816651	1.667217	1.555678
N	2.241578	-1.963062	1.085910
C	3.440473	-1.581328	0.441140
C	3.402922	-0.196799	0.181938
C	4.535288	-2.361893	0.099020
C	5.621953	-1.738127	-0.507192
C	5.613701	-0.369541	-0.752993
C	4.510005	0.403485	-0.413297
H	4.538301	-3.419853	0.305240
H	6.486434	-2.333196	-0.780268
H	6.473742	0.103068	-1.214534
H	4.524202	1.466603	-0.602606
C	1.877689	-3.280602	1.367013
O	2.654493	-4.192696	1.185346
C	0.484447	-3.528760	1.874365
H	0.347229	-4.608153	1.921221
H	0.341518	-3.084008	2.859073
H	-0.256467	-3.076458	1.215991
C	2.207421	2.860929	0.413033
C	2.239898	3.887400	1.359826
C	2.832240	5.109558	1.066305
C	3.387061	5.331114	-0.187915
C	3.338974	4.324041	-1.146917
C	2.751629	3.101796	-0.852338
H	1.804000	3.720065	2.339742
H	2.857121	5.890448	1.818611
H	3.846065	6.285531	-0.421012
H	3.755234	4.493095	-2.134059
H	2.695355	2.324613	-1.608042

4-Eg

charge, 2S+1 = 0, 1
--- OPT at ωB97X-D/6-311G(d,p) in SMD ---
E(ele) = -2241.61753998 a.u.
Correction to G = 0.571054 a.u.(RRHO)
Correction to G = 0.580868 a.u.(quasi-RRHO)
--- SP at ωB97X-D/def2-TZVP in SMD ---
E(ele) = -2241.85338724 a.u.

P	2.308972	-0.145697	0.033030
C	2.268668	1.656919	0.151071
C	1.994357	2.438902	-0.970009
C	2.384121	2.251960	1.411809
C	1.830535	3.809110	-0.825082
C	2.208639	3.620086	1.546855
C	1.928395	4.397562	0.428926
H	1.873052	1.989127	-1.944611
H	2.600386	1.653153	2.288818
H	1.606626	4.412984	-1.696040
H	2.283446	4.076252	2.526483
H	1.782281	5.466101	0.537819
C	2.837643	-0.704903	-1.601284
C	4.072037	-1.337831	-1.762217
C	1.994946	-0.503095	-2.700292
C	4.467838	-1.756567	-3.025376
C	2.406106	-0.926894	-3.956222
C	3.638500	-1.549262	-4.120579
H	4.721117	-1.513529	-0.913065
H	1.022254	-0.028694	-2.563139
H	5.424528	-2.249809	-3.150312
H	1.754672	-0.771529	-4.808566
H	3.951214	-1.879535	-5.104718
C	3.500067	-0.732734	1.261046
C	4.731441	-0.084165	1.391094
C	3.209664	-1.851638	2.040577
C	5.666129	-0.559430	2.298244
C	4.150594	-2.317489	2.950428
C	5.374969	-1.674616	3.077883
H	4.957158	0.789959	0.789952
H	2.253911	-2.354439	1.945259
H	6.620448	-0.056559	2.400195
H	3.921843	-3.182920	3.560763
H	6.105682	-2.040855	3.789872
C	0.700181	-0.834543	0.479593
C	-0.033135	-0.149502	1.593468
H	0.671214	0.280107	2.312497

H	-0.631133	-0.893632	2.118523
C	0.222302	-1.860805	-0.229943
H	0.740380	-2.234955	-1.106218
C	-1.037882	-2.582937	0.107403
O	-1.396598	-2.870088	1.222389
O	-1.672921	-2.942085	-1.003288
C	-2.917607	-3.632127	-0.818722
H	-3.557773	-3.087836	-0.124823
H	-3.376565	-3.671945	-1.803595
H	-2.731268	-4.640549	-0.441653
C	-1.580541	0.541943	-1.355974
O	-0.542510	0.973370	-1.904791
C	-1.910375	0.480094	-0.003043
C	-0.957868	0.978182	1.035173
H	-0.305188	1.686053	0.514818
N	-2.702841	-0.010153	-2.094935
C	-3.708182	-0.364629	-1.170860
C	-3.217854	-0.081891	0.128146
C	-4.966677	-0.906885	-1.374190
C	-5.749787	-1.198009	-0.255464
C	-5.276531	-0.945419	1.029787
C	-4.018389	-0.388385	1.231704
H	-5.321477	-1.105599	-2.373456
H	-6.737059	-1.624200	-0.396768
H	-5.898716	-1.180981	1.887376
H	-3.662975	-0.188335	2.235670
C	-2.767582	-0.271379	-3.452964
O	-3.763513	-0.751953	-3.959091
C	-1.553203	0.027539	-4.296324
H	-1.835587	-0.139891	-5.335254
H	-1.186631	1.039756	-4.138260
H	-0.742215	-0.651131	-4.021003
C	-1.632771	1.769876	2.145133
C	-2.316484	2.937365	1.793226
C	-2.959562	3.707248	2.748844
C	-2.936520	3.324992	4.087936
C	-2.263018	2.169057	4.451476
C	-1.615033	1.399041	3.487302
H	-2.350584	3.227397	0.747959
H	-3.487556	4.606233	2.449363
H	-3.444707	3.922264	4.836961
H	-2.242317	1.854916	5.489692
H	-1.108326	0.493452	3.800404

ts5-Eg (syn)

charge, 2S+1 = 0, 1
--- OPT at ωB97X-D/6-311G(d,p) in SMD ---
E(ele) = -2241.60077904 a.u.
Correction to G = 0.569631 a.u.(RRHO)
Correction to G = 0.579356 a.u.(quasi-RRHO)
imaginary frequency = 311i
--- SP at ωB97X-D/def2-TZVP in SMD ---
E(ele) = -2241.83603420 a.u.

P	-2.282512	-0.111764	0.077727
C	-2.647534	-1.351696	-1.202033
C	-3.699701	-2.258307	-1.065266
C	-1.800900	-1.430287	-2.312711
C	-3.910756	-3.226459	-2.039268
C	-2.024072	-2.396981	-3.283095
C	-3.077371	-3.294444	-3.148248
H	-4.350427	-2.221454	-0.200213
H	-0.964712	-0.743040	-2.405048
H	-4.726294	-3.931301	-1.925736
H	-1.365258	-2.453207	-4.141921
H	-3.243686	-4.052990	-3.904907
C	-2.487530	1.573141	-0.567509
C	-2.489089	1.854379	-1.932006
C	-2.604806	2.615625	0.356854
C	-2.605993	3.168199	-2.366948
C	-2.705079	3.927445	-0.084440
C	-2.707309	4.204073	-1.446796
H	-2.397135	1.059957	-2.660438
H	-2.619634	2.403889	1.420487
H	-2.608800	3.380617	-3.429616
H	-2.785797	4.731925	0.637110
H	-2.789433	5.228702	-1.791269
C	-3.614777	-0.277564	1.313748
C	-4.918011	0.128511	1.015143
C	-3.322055	-0.817219	2.562775
C	-5.921126	-0.018292	1.961450
C	-4.332107	-0.965149	3.507186
C	-5.628356	-0.568300	3.206649
H	-5.146837	0.563515	0.047544
H	-2.301657	-1.101786	2.797177
H	-6.931191	0.300372	1.730976
H	-4.101516	-1.383870	4.479935
H	-6.413775	-0.679536	3.945812
C	-0.729382	-0.344707	0.806531
C	-0.064647	-1.682170	0.845166
H	-0.730475	-2.519330	0.617003
H	0.375933	-1.851064	1.828985
C	0.163056	0.718725	0.969670
H	-0.089326	1.691316	0.554900
C	1.039876	0.863915	2.161494
O	1.223101	0.055441	3.039260
O	1.609889	2.076430	2.145756
C	2.571422	2.337121	3.168668
H	3.335762	1.559948	3.183271
H	3.016559	3.297167	2.914896
H	2.085813	2.392141	4.145927
C	1.464603	0.704971	-1.265851
O	0.570846	0.670445	-2.101007
C	1.737622	-0.231512	-0.199386
C	1.092055	-1.593770	-0.225017
H	0.589891	-1.663797	-1.193859
N	2.453899	1.737696	-1.186167
C	3.455373	1.329566	-0.273967
C	3.055315	0.113793	0.310121
C	4.652807	1.939426	0.063673
C	5.470360	1.297597	0.993817
C	5.096207	0.088210	1.570024
C	3.882387	-0.506637	1.240065
H	4.942086	2.874400	-0.389420
H	6.417587	1.752605	1.261918
H	5.752250	-0.395260	2.285446
H	3.585035	-1.441510	1.697337
C	2.415784	2.975470	-1.828507
O	3.345840	3.747050	-1.741121
C	1.170665	3.326422	-2.598550
H	1.269344	4.365375	-2.910462
H	1.056467	2.678955	-3.468058
H	0.274711	3.194471	-1.989909
C	2.080732	-2.746617	-0.183582
C	2.076702	-3.723209	0.809179
C	3.007274	-4.759673	0.796981
C	3.956550	-4.838653	-0.210773
C	3.966364	-3.873659	-1.213840
C	3.038371	-2.844552	-1.198083
H	1.350825	-3.685322	1.612090
H	2.986257	-5.505683	1.584260
H	4.683646	-5.643142	-0.217637
H	4.703406	-3.921441	-2.008100
H	3.059843	-2.089618	-1.977942

ts5-Eg (anti)

charge, 2S+1 = 0, 1
--- OPT at ωB97X-D/6-311G(d,p) in SMD ---
E(ele) = -2241.59002475 a.u.
Correction to G = 0.571038 a.u.(RRHO)
Correction to G = 0.580630 a.u.(quasi-RRHO)
imaginary frequency = 328i
--- SP at ωB97X-D/def2-TZVP in SMD ---
E(ele) = -2241.82575068 a.u.

P	2.198581	0.332849	0.053396
C	2.123194	0.911655	1.785671
C	1.908594	-0.029715	2.796870
C	2.238794	2.261211	2.119419
C	1.772824	0.379469	4.115027
C	2.109615	2.664637	3.443221
C	1.865852	1.728591	4.439322
H	1.846890	-1.085928	2.564002
H	2.422048	3.005098	1.354438
H	1.595332	-0.358192	4.888779
H	2.197279	3.715793	3.692353
H	1.755535	2.047721	5.469327
C	2.412930	1.785841	-1.006033
C	1.420692	2.771919	-1.038833
C	3.543144	1.909992	-1.812475
C	1.564144	3.868531	-1.875203
C	3.679752	3.013638	-2.645892
C	2.693233	3.990175	-2.678465
H	0.535563	2.685209	-0.417061
H	4.314892	1.149367	-1.796653
H	0.788234	4.624594	-1.899477
H	4.559022	3.105181	-3.272669
H	2.801479	4.847662	-3.332844
C	3.720574	-0.656621	-0.019655
C	4.826404	-0.321570	0.764226
C	3.791881	-1.727572	-0.909986
C	5.998762	-1.055116	0.651859
C	4.967388	-2.460666	-1.013309
C	6.068653	-2.124138	-0.235723
H	4.771870	0.505041	1.464191
H	2.926040	-1.984940	-1.510380
H	6.856575	-0.796103	1.261530
H	5.019403	-3.297044	-1.700455
H	6.984302	-2.698983	-0.317027
C	0.887506	-0.655447	-0.536044
C	0.388733	-1.824437	0.246763
H	1.098682	-2.236025	0.968004
H	0.105322	-2.632173	-0.427498
C	-0.059018	-0.175426	-1.434337
H	-0.044041	0.856725	-1.766884
C	-0.659161	-1.065329	-2.469541
O	-0.409732	-2.230528	-2.653044
O	-1.485445	-0.361362	-3.256088
C	-2.142439	-1.104720	-4.282083
H	-2.867336	-0.422472	-4.722851
H	-1.425496	-1.428183	-5.040611
H	-2.638149	-1.978186	-3.857732
C	-2.864332	-0.812756	-0.710945
O	-3.147083	-1.911316	-1.155079
C	-1.731812	-0.401208	0.091749
C	-0.908203	-1.296698	0.982383
H	-0.539400	-0.645126	1.778674
N	-3.629714	0.377357	-0.995026
C	-3.059726	1.470513	-0.307583
C	-1.950868	0.994330	0.417973
C	-3.444593	2.798418	-0.250147
C	-2.717709	3.660421	0.577887
C	-1.652465	3.195336	1.337107
C	-1.264246	1.857150	1.263664
H	-4.285908	3.151893	-0.825047
H	-3.011562	4.702541	0.637278
H	-1.118884	3.872238	1.996300
H	-0.442437	1.497407	1.870613
C	-4.759326	0.466794	-1.806965
O	-5.243502	1.547338	-2.071405
C	-5.377615	-0.807066	-2.316692
H	-5.603745	-1.490563	-1.497868
H	-6.287634	-0.528130	-2.846595
H	-4.699000	-1.338283	-2.982814
C	-1.652829	-2.415393	1.683288
C	-1.955679	-2.286601	3.039549
C	-2.613831	-3.299060	3.727821
C	-2.976042	-4.465631	3.066100
C	-2.678548	-4.605505	1.715029
C	-2.023149	-3.591290	1.028645
H	-1.675675	-1.375713	3.561151
H	-2.842398	-3.175463	4.781073
H	-3.489102	-5.259501	3.597966
H	-2.963559	-5.509502	1.187558
H	-1.826565	-3.697643	-0.030223

5-Eg

charge, 2S+1 = 0, 1
--- OPT at ωB97X-D/6-311G(d,p) in SMD ---
E(ele) = -2241.62561134 a.u.
Correction to G = 0.569083 a.u.(RRHO)
Correction to G = 0.580118 a.u.(quasi-RRHO)
--- SP at ωB97X-D/def2-TZVP in SMD ---
E(ele) = -2241.86214317 a.u.

P	-2.398481	0.170044	0.192872
C	-2.756849	0.294687	1.973391
C	-2.204145	1.367083	2.678602
C	-3.528584	-0.647788	2.649662
C	-2.430421	1.498148	4.041764
C	-3.751767	-0.513803	4.014381
C	-3.205520	0.557184	4.710517
H	-1.593755	2.095053	2.154901
H	-3.941732	-1.496619	2.118691
H	-1.996342	2.331866	4.581671
H	-4.348231	-1.254194	4.535115

H	-3.378780	0.656407	5.776344
C	-3.252846	-1.371244	-0.370346
C	-4.634865	-1.405551	-0.573829
C	-2.497237	-2.514189	-0.612500
C	-5.249298	-2.572153	-1.008367
C	-3.112308	-3.680909	-1.052633
C	-4.486824	-3.710657	-1.251475
H	-5.233980	-0.518198	-0.396477
H	-1.431228	-2.488009	-0.423042
H	-6.322223	-2.591806	-1.164085
H	-2.516418	-4.567993	-1.236723
H	-4.966568	-4.620056	-1.596519
C	-3.375459	1.437479	-0.700143
C	-3.958534	2.519683	-0.042911
C	-3.474469	1.348547	-2.090987
C	-4.627789	3.499519	-0.766788
C	-4.131947	2.335856	-2.812469
C	-4.710979	3.412636	-2.150844
H	-3.902646	2.599488	1.036531
H	-3.044947	0.500508	-2.614194
H	-5.086396	4.332054	-0.245199
H	-4.196504	2.260130	-3.892004
H	-5.230021	4.180589	-2.713367
C	-0.758367	0.396004	-0.083944
C	-0.185750	0.877100	-1.403916
H	-0.732630	1.716816	-1.846163
H	-0.134119	0.099390	-2.180094
C	0.346822	0.181653	0.929019
H	0.400628	0.976636	1.687702
C	0.363353	-1.107425	1.728216
O	-0.452427	-1.991482	1.698368
O	1.444441	-1.139377	2.519984
C	1.656871	-2.347173	3.251363
H	2.570484	-2.189879	3.821410
H	0.819935	-2.545299	3.923310
H	1.782367	-3.187780	2.565719
C	2.828406	0.850175	0.865550
O	2.825858	1.861175	1.518166
C	1.643748	0.301983	0.083118
C	1.224620	1.334285	-1.011268
H	1.117803	2.280475	-0.470866
N	3.931461	0.000605	0.654603
C	3.519384	-1.130060	-0.109418
C	2.174145	-0.994828	-0.449321
C	4.251793	-2.242230	-0.497105
C	3.594199	-3.229243	-1.229388
C	2.249462	-3.115607	-1.555587
C	1.530162	-1.990314	-1.161777
H	5.292622	-2.339997	-0.235434
H	4.151645	-4.105592	-1.540723
H	1.757715	-3.902709	-2.115590
H	0.479507	-1.888876	-1.403936
C	5.241205	0.208035	1.124675
O	6.089229	-0.633758	0.946172
C	5.541050	1.501088	1.828134
H	5.285619	2.357609	1.202777
H	4.960441	1.593283	2.746386
H	6.606393	1.501052	2.053168
C	2.253963	1.536350	-2.097771
C	2.368893	0.685847	-3.198487
C	3.370622	0.874520	-4.142236
C	4.279400	1.916582	-4.003520
C	4.175905	2.772838	-2.914405
C	3.170790	2.582835	-1.974114
H	1.679376	-0.140535	-3.318950
H	3.443340	0.199534	-4.987957
H	5.062332	2.060472	-4.739818
H	4.876491	3.592341	-2.796493
H	3.094261	3.255419	-1.125259

e-iv

charge, 2S+1 = 0, 1
--- OPT at ωB97X-D/6-311G(d,p) in SMD ---
E(ele) = -629.76298999 a.u.
Correction to G = 0.140649 a.u.(RRHO)
Correction to G = 0.141649 a.u.(quasi-RRHO)
--- SP at ωB97X-D/def2-TZVP in SMD ---
E(ele) = -629.84185098 a.u.

C	-2.823509	2.962530	0.208063
O	-1.759572	3.179715	-0.310325
C	-3.685993	1.754793	0.025996
C	-3.331927	0.751236	-0.771951
H	-2.393305	0.795427	-1.313375
H	-3.957163	-0.124734	-0.903288
N	-3.505210	3.809152	1.115365
C	-4.735640	3.197806	1.502010
C	-4.864806	1.960805	0.858613
C	-5.718986	3.658561	2.364935
C	-6.835055	2.848237	2.566884
C	-6.972021	1.618373	1.932217
C	-5.980363	1.166481	1.069309
H	-5.621774	4.610290	2.861051
H	-7.612981	3.193662	3.238316
H	-7.852441	1.012347	2.110719
H	-6.075344	0.209870	0.567845
C	-3.062780	5.062694	1.581662
O	-3.742894	5.691116	2.356677
C	-1.739511	5.572610	1.086531
H	-1.734134	5.669420	0.000288
H	-1.578395	6.543116	1.552839
H	-0.930427	4.889535	1.348432

ts4-Za'

charge, 2S+1 = 0, 1
--- OPT at ωB97X-D/6-311G(d,p) in SMD ---
E(ele) = -2010.54700850 a.u.
Correction to G = 0.490065 a.u.(RRHO)
Correction to G = 0.498472 a.u.(quasi-RRHO)
imaginary frequency = 230i
--- SP at ωB97X-D/def2-TZVP in SMD ---
E(ele) = -2010.75823147 a.u.

P	-1.606063	-0.372706	0.177093
C	-1.201207	-2.146136	0.311344
C	-1.263289	-2.750818	1.571189
C	-0.791115	-2.894521	-0.790870
C	-0.910463	-4.083118	1.722860
C	-0.441919	-4.230417	-0.632323
C	-0.497622	-4.824391	0.621133
H	-1.580529	-2.180056	2.436412
H	-0.737843	-2.449076	-1.775082
H	-0.957388	-4.541733	2.703540
H	-0.123639	-4.803613	-1.495214
H	-0.221611	-5.865868	0.740917
C	-2.088652	-0.065133	-1.541559
C	-1.103695	-0.098593	-2.527247
C	-3.414967	0.196328	-1.882054
C	-1.454253	0.116939	-3.855161
C	-3.754841	0.401337	-3.210576
C	-2.776921	0.361282	-4.198738
H	-0.062928	-0.286815	-2.277221
H	-4.175384	0.275982	-1.116194
H	-0.683262	0.094360	-4.616836
H	-4.785855	0.609691	-3.472032
H	-3.046956	0.529885	-5.235242
C	-3.005691	-0.169266	1.309235
C	-4.104587	-1.022553	1.184821
C	-2.965264	0.772529	2.333861
C	-5.167407	-0.914073	2.069961
C	-4.026929	0.866455	3.223741
C	-5.128367	0.030494	3.089456
H	-4.129135	-1.777984	0.406386
H	-2.108578	1.426335	2.440920
H	-6.020989	-1.573908	1.968230
H	-3.992124	1.598168	4.022352
H	-5.956458	0.110096	3.784684
C	-0.159058	0.569013	0.753598
C	0.860406	-0.156095	1.278492
H	0.863598	-1.234548	1.335871
H	1.706406	0.361509	1.716194
C	-0.079318	1.985243	0.576399
H	0.698198	2.488583	1.136932
C	-1.180593	2.790975	0.156927
O	-2.291875	2.392055	-0.171069
O	-0.869556	4.109500	0.120287
C	-1.907696	4.974275	-0.325490
H	-1.491661	5.980295	-0.287230
H	-2.208810	4.734304	-1.347865
H	-2.783293	4.908768	0.324879
C	2.588329	-0.089668	-1.209962
O	1.933823	-0.717216	-2.025824
C	2.475989	1.304070	-0.814443
C	1.457956	2.130381	-1.193530
H	0.763182	1.817220	-1.962502
H	1.504511	3.194653	-1.000593
N	3.708283	-0.629954	-0.493441
C	4.290841	0.391984	0.299894
C	3.547813	1.571122	0.124269
C	5.377498	0.348374	1.159818
C	5.708163	1.513383	1.853300
C	4.973821	2.683623	1.695694
C	3.885297	2.718631	0.828846
H	5.945523	-0.559346	1.286577
H	6.556881	1.497386	2.527923
H	5.251065	3.573773	2.249425
H	3.309820	3.629266	0.699905
C	4.133022	-1.961203	-0.496235
O	5.118696	-2.293082	0.125163
C	3.320220	-2.964276	-1.269038
H	3.774855	-3.939482	-1.099617
H	2.280475	-2.965715	-0.937715
H	3.310738	-2.726832	-2.332781

ts4-Zg'

charge, 2S+1 = 0, 1
--- OPT at ωB97X-D/6-311G(d,p) in SMD ---
E(ele) = -2010.54478586 a.u.
Correction to G = 0.490040 a.u.(RRHO)
Correction to G = 0.498405 a.u.(quasi-RRHO)
imaginary frequency = 371i
--- SP at ωB97X-D/def2-TZVP in SMD ---
E(ele) = -2010.75580680 a.u.

P	1.877394	0.032925	0.039629
C	1.997314	-0.325556	-1.729628
C	3.266236	-0.523904	-2.283195
C	0.868603	-0.327725	-2.540480
C	3.393817	-0.757661	-3.644163
C	1.008927	-0.541586	-3.906182
C	2.263962	-0.766017	-4.456227
H	4.153806	-0.484666	-1.660771
H	-0.118435	-0.154083	-2.131387
H	4.376332	-0.920809	-4.071551
H	0.126336	-0.532786	-4.534810
H	2.366366	-0.939873	-5.521639
C	2.440392	-1.341326	1.078280
C	3.144864	-2.418783	0.547868
C	2.171116	-1.284474	2.446183
C	3.591553	-3.428723	1.388349
C	2.620945	-2.299695	3.279921
C	3.333881	-3.369322	2.752827
H	3.319437	-2.492291	-0.518098
H	1.599831	-0.460015	2.857751
H	4.130891	-4.271469	0.971910
H	2.404758	-2.256510	4.340992
H	3.679737	-4.163655	3.404535
C	3.100220	1.370112	0.255877
C	3.144049	2.397334	-0.691632
C	3.965496	1.402298	1.347735
C	4.034950	3.448742	-0.536543
C	4.861289	2.454718	1.493623
C	4.894649	3.478466	0.556348
H	2.484414	2.376734	-1.551865
H	3.952097	0.607952	2.083858
H	4.060912	4.243081	-1.273362
H	5.534976	2.469930	2.342439
H	5.594480	4.298167	0.673014
C	0.295186	0.638703	0.700947
C	0.217962	1.990520	1.018649
H	1.078203	2.643561	0.966261
H	-0.544814	2.283102	1.732020
C	-0.772551	-0.232898	0.899367
H	-1.594497	0.091738	1.522803
C	-0.803719	-1.573553	0.404998
O	0.059839	-2.101819	-0.285751
O	-1.922035	-2.233711	0.771046
C	-2.011073	-3.594974	0.365782
H	-3.016055	-3.916840	0.633476
H	-1.854672	-3.703437	-0.708751
H	-1.270556	-4.203059	0.892352
C	-2.625464	0.781481	-1.227547
O	-2.106709	0.453611	-2.280654
C	-2.335645	1.930696	-0.386083
C	-1.239735	2.734514	-0.550349
H	-0.631193	2.606602	-1.438362

N	-3.747802	0.111730	-0.635521
C	-4.165769	0.831702	0.509244
C	-3.321857	1.944424	0.673289
C	-5.190474	0.569878	1.405156
C	-5.360444	1.446212	2.477632
C	-4.529654	2.547447	2.653735
C	-3.500867	2.801488	1.751162
H	-5.830327	-0.288037	1.274267
H	-6.159352	1.257083	3.185971
H	-4.684181	3.211303	3.497116
H	-2.850680	3.660428	1.881415
C	-4.283609	-1.114463	-1.034269
O	-5.242551	-1.585117	-0.462182
C	-3.637455	-1.824665	-2.193148
H	-3.796399	-1.266864	-3.117486
H	-2.559379	-1.914647	-2.066139
H	-4.104341	-2.806213	-2.265041
H	-1.184621	3.703298	-0.069115

ts4-Ea'

charge, 2S+1 = 0, 1
--- OPT at ωB97X-D/6-311G(d,p) in SMD ---
E(ele) = -2010.54756507 a.u.
Correction to G = 0.490043 a.u.(RRHO)
Correction to G = 0.497982 a.u.(quasi-RRHO)
imaginary frequency = 213i
--- SP at ωB97X-D/def2-TZVP in SMD ---
E(ele) = -2010.75885426 a.u.

P	1.631952	-0.600291	-0.003892
C	1.252828	-1.742676	1.351242
C	0.757810	-3.011483	1.032351
C	1.469985	-1.396970	2.684024
C	0.464432	-3.914093	2.043641
C	1.187097	-2.312244	3.689659
C	0.680627	-3.565478	3.372427
H	0.612652	-3.299557	-0.002944
H	1.859198	-0.422497	2.947064
H	0.073417	-4.892685	1.791823
H	1.358104	-2.038331	4.723927
H	0.454902	-4.274068	4.161033
C	2.655503	0.757460	0.614919
C	3.975657	0.915819	0.195048
C	2.079317	1.679610	1.494650
C	4.722813	1.988485	0.665546
C	2.836703	2.745190	1.959135
C	4.156135	2.899800	1.547026
H	4.420204	0.218928	-0.505085
H	1.043188	1.565333	1.804900
H	5.747254	2.113099	0.334938
H	2.389843	3.459928	2.640378
H	4.741610	3.737421	1.908983
C	2.604822	-1.563046	-1.191246
C	3.641366	-2.378554	-0.728654
C	2.322281	-1.491351	-2.554057
C	4.400237	-3.106829	-1.632324
C	3.085418	-2.227866	-3.451787
C	4.122928	-3.029494	-2.993721
H	3.850059	-2.449372	0.333714
H	1.503965	-0.875872	-2.909545
H	5.203521	-3.739749	-1.273882
H	2.862259	-2.176101	-4.510843
H	4.714407	-3.603175	-3.698266
C	0.182200	0.043438	-0.855210
C	-0.953886	-0.693155	-0.816377
H	-1.035446	-1.601110	-0.232415
H	-1.794741	-0.414245	-1.435344
C	0.399077	1.282307	-1.534205
H	1.328121	1.815309	-1.379215
C	-0.285580	1.593303	-2.763025
O	-1.207388	0.990170	-3.280654
O	0.225096	2.724920	-3.330118
C	-0.401015	3.141477	-4.535108
H	-0.320488	2.376199	-5.311209
H	-1.458854	3.367836	-4.377631
H	0.126238	4.042439	-4.848047
C	-1.887302	1.026325	1.405899
O	-0.943960	0.967329	2.180738
C	-2.043526	1.850868	0.221444
C	-1.045751	2.653209	-0.248930
H	-0.193703	2.873253	0.379534
H	-1.223428	3.341140	-1.066690
N	-3.107311	0.293377	1.560612
C	-4.014516	0.680012	0.539128
C	-3.374217	1.601701	-0.304111
C	-5.315281	0.262944	0.298501
C	-5.970344	0.785024	-0.815904
C	-5.343234	1.692539	-1.664039
C	-4.039771	2.105428	-1.412521
H	-5.800265	-0.441213	0.955595
H	-6.988100	0.471550	-1.020303
H	-5.872385	2.077186	-2.528871
H	-3.541153	2.800158	-2.078330
C	-3.354263	-0.719603	2.490016
O	-4.459202	-1.204682	2.597317
C	-2.194118	-1.213954	3.309140
H	-2.546318	-2.069046	3.884548
H	-1.363940	-1.503495	2.663649
H	-1.816882	-0.435159	3.971507

ts4-Eg'

charge, 2S+1 = 0, 1
--- OPT at ωB97X-D/6-311G(d,p) in SMD ---
E(ele) = -2010.55120716 a.u.
Correction to G = 0.493739 a.u.(RRHO)
Correction to G = 0.500362 a.u.(quasi-RRHO)
imaginary frequency = 260i
--- SP at ωB97X-D/def2-TZVP in SMD ---
E(ele) = -2010.76150227 a.u.

P	1.934384	0.106854	0.001760
C	2.652252	0.519481	-1.607107
C	1.890941	0.296754	-2.758913
C	3.923789	1.084238	-1.704011
C	2.414738	0.628673	-4.000062
C	4.436121	1.415404	-2.952316
C	3.685177	1.186255	-4.097891
H	0.894041	-0.128080	-2.675681
H	4.512254	1.277222	-0.815199
H	1.824333	0.454878	-4.892184
H	5.422843	1.857350	-3.025942
H	4.088400	1.447453	-5.069809
C	1.729050	-1.688957	0.150358
C	1.526682	-2.224211	1.426823
C	1.792440	-2.535113	-0.954871
C	1.372228	-3.592947	1.587364
C	1.648653	-3.905834	-0.783712
C	1.434627	-4.434509	0.482242
H	1.493909	-1.575119	2.294519
H	1.951620	-2.138332	-1.948365
H	1.206518	-4.000857	2.577333
H	1.698053	-4.559338	-1.646619
H	1.316222	-5.504476	0.609799
C	3.136794	0.545150	1.288528
C	4.278116	-0.244329	1.456375
C	2.933886	1.663262	2.094707
C	5.216392	0.095817	2.418985
C	3.876525	1.993436	3.061102
C	5.015433	1.215184	3.220959
H	4.430753	-1.124779	0.841409
H	2.041461	2.266701	1.973574
H	6.101222	-0.516414	2.548009
H	3.714140	2.859392	3.691898
H	5.747393	1.476118	3.976894
C	0.437553	1.059279	0.297713
C	0.413874	2.328353	-0.260372
H	1.251276	2.694407	-0.844361
H	-0.254893	3.058242	0.172949
C	-0.541165	0.453483	1.079801
H	-0.495542	-0.602676	1.303893
C	-1.595223	1.185980	1.731106
O	-1.754023	2.393825	1.770629
O	-2.444189	0.334025	2.363342
C	-3.551075	0.940102	3.016766
H	-3.224216	1.504769	3.895234
H	-4.087229	1.609273	2.342931
H	-4.200277	0.121079	3.324178
C	-1.857243	-0.356685	-1.399491
O	-0.902387	-0.948825	-1.879682
C	-2.125718	1.072005	-1.398815
C	-1.239859	1.979406	-1.900480
H	-0.440601	1.650294	-2.548387
H	-1.502073	3.029431	-1.953845
N	-2.978973	-0.988320	-0.778213
C	-3.950839	-0.002505	-0.469876
C	-3.434887	1.259480	-0.804112
C	-5.220184	-0.143149	0.070364
C	-5.968854	1.013571	0.282280
C	-5.461740	2.272000	-0.029011
C	-4.188145	2.401487	-0.571716
H	-5.608585	-1.118198	0.317713
H	-6.964864	0.923589	0.701711
H	-6.061808	3.156323	0.154793
H	-3.784907	3.379058	-0.809881
C	-3.067545	-2.326868	-0.391547
O	-4.097231	-2.771908	0.065368
C	-1.833465	-3.175219	-0.530781
H	-1.566004	-3.308756	-1.578919
H	-0.978408	-2.702143	-0.046706
H	-2.049544	-4.137242	-0.068176

8

charge, 2S+1 = 0, 1
--- OPT at ωB97X-D/6-311G(d,p) in SMD ---
E(ele) = -575.55053479 a.u.
Correction to G = 0.142290 a.u.(RRHO)
Correction to G = 0.144572 a.u.(quasi-RRHO)
--- SP at ωB97X-D/def2-TZVP in SMD ---
E(ele) = -575.62460138 a.u.

C	1.819527	-0.284780	0.014089
C	2.617494	-1.310604	0.111985
H	2.947508	-1.779709	-0.812652
C	1.026015	0.748423	-0.077887
H	1.421342	1.756734	-0.149575
C	-0.454572	0.653320	-0.091636
O	-1.174579	1.614828	-0.188502
O	-0.894470	-0.601956	0.019359
C	-2.314109	-0.766240	0.017383
H	-2.484514	-1.836104	0.119800
H	-2.766774	-0.230630	0.854063
H	-2.744373	-0.403421	-0.918084
C	3.107373	-1.897676	1.373283
C	4.021076	-2.951796	1.322902
C	2.681225	-1.431955	2.620935
C	4.503357	-3.526382	2.492338
H	4.356373	-3.322573	0.359705
C	3.163544	-2.006196	3.786393
H	1.964574	-0.619029	2.673267
C	4.076879	-3.055291	3.727132
H	5.213166	-4.344128	2.436802
H	2.824068	-1.636115	4.747353
H	4.451606	-3.503061	4.640583

trans-7-Za

charge, 2S+1 = 0, 1
--- OPT at ωB97X-D/6-311G(d,p) in SMD ---
E(ele) = -1436.42806231 a.u.
Correction to G = 0.388120 a.u.(RRHO)
Correction to G = 0.394946 a.u.(quasi-RRHO)
--- SP at ωB97X-D/def2-TZVP in SMD ---
E(ele) = -1436.60152792 a.u.

C	-0.919891	2.321384	0.708237
C	0.400133	1.678736	1.036359
H	0.473319	1.644491	2.132502
C	-1.890268	1.418827	0.580970
H	-1.072081	3.393838	0.691979
C	-3.314735	1.796618	0.433034
O	-3.704254	2.829475	-0.052996
O	-4.119575	0.851796	0.923268
C	-5.522889	1.090327	0.796055
H	-5.811600	1.994640	1.334826
H	-5.799507	1.190649	-0.255142
H	-6.008145	0.219205	1.230928
C	1.657657	2.327606	0.510664
C	1.645222	3.183651	-0.587817
C	2.878939	2.032622	1.117454
C	2.827364	3.728919	-1.074079
H	0.706343	3.424228	-1.074962
C	4.062510	2.568637	0.628586
H	2.901294	1.379090	1.984570
C	4.039465	3.419676	-0.470428
H	2.800410	4.393101	-1.930733
H	5.003435	2.326173	1.109816
H	4.962210	3.841288	-0.852755

C	0.954785	−0.790026	1.413327
O	0.908197	−0.865734	2.614908
C	0.166961	0.205874	0.567952
C	−1.375749	0.007505	0.790337
H	−1.512312	−0.241911	1.849923
N	1.723879	−1.591751	0.557776
C	1.542369	−1.157941	−0.792076
C	0.642002	−0.096839	−0.822726
C	2.125545	−1.645216	−1.953024
C	1.782516	−1.032669	−3.155193
C	0.890479	0.031053	−3.198490
C	0.314492	0.504050	−2.024941
H	2.820309	−2.468189	−1.923090
H	2.228241	−1.399192	−4.072954
H	0.642187	0.493269	−4.146725
H	−0.384459	1.331069	−2.049226
C	2.554355	−2.664395	0.946353
O	3.184012	−3.269972	0.114019
C	2.616762	−3.018614	2.404805
H	3.278883	−3.878247	2.493726
H	2.998420	−2.186169	2.997034
H	1.628372	−3.260517	2.797479
C	−1.969618	−1.113643	−0.029107
C	−2.532803	−0.902344	−1.285775
C	−3.011169	−1.967693	−2.036030
C	−2.928448	−3.264277	−1.541675
C	−2.372383	−3.486266	−0.288325
C	−1.901984	−2.416100	0.462650
H	−2.592766	0.102769	−1.689669
H	−3.445100	−1.785029	−3.012797
H	−3.297714	−4.096748	−2.130000
H	−2.308225	−4.493228	0.108796
H	−1.480368	−2.593918	1.447648

trans-ts3-E

charge, 2S+1 = 0, 1
--- OPT at ωB97X-D/6-311G(d,p) in SMD ---
E(ele) = -1611.77715507 a.u.
Correction to G = 0.394499 a.u.(RRHO)
Correction to G = 0.403136 a.u.(quasi-RRHO)
imaginary frequency = 216i
--- SP at ωB97X-D/def2-TZVP in SMD ---
E(ele) = -1611.93672468 a.u.

P	0.853051	0.364644	0.469828
C	2.133907	0.903762	−0.705324
C	2.732812	2.164416	−0.656510
C	2.456508	0.035050	−1.755051
C	3.637703	2.547666	−1.637729
C	3.357368	0.427585	−2.736486
C	3.948847	1.683603	−2.681208
H	2.506358	2.847330	0.153967
H	2.012856	−0.952758	−1.802337
H	4.103800	3.525079	−1.582942
H	3.598263	−0.254456	−3.543957
H	4.654908	1.986742	−3.446092
C	1.433861	−1.272418	1.015510
C	0.504774	−2.303406	1.147344
C	2.782996	−1.515833	1.295061
C	0.919504	−3.562917	1.567991
C	3.189619	−2.770997	1.721766
C	2.256330	−3.795293	1.859770
H	−0.534886	−2.139727	0.887184
H	3.513793	−0.723430	1.172372
H	0.192785	−4.362252	1.655010
H	4.235750	−2.953962	1.940062
H	2.577459	−4.778479	2.185862
C	0.955899	1.478888	1.909889
C	0.401267	2.756807	1.766670
C	1.461005	1.091583	3.152250
C	0.388045	3.641376	2.836521
C	1.434511	1.976708	4.223066
C	0.905104	3.251976	4.067075
H	−0.023749	3.058901	0.814293
H	1.873315	0.098595	3.288000
H	−0.036255	4.631093	2.711195
H	1.830617	1.666420	5.183430
H	0.887785	3.939201	4.905434
C	−1.114120	0.070061	−0.763982
C	−2.050951	0.552371	0.039343
H	−1.783913	0.924346	1.022890
C	−0.818516	−0.559130	−1.931269
H	−0.295910	−0.036178	−2.723407
C	−1.005690	−1.985478	−2.064811
O	−1.579721	−2.729880	−1.291466
O	−0.458421	−2.444477	−3.219387
C	−0.620473	−3.836412	−3.462363
H	−1.677474	−4.107522	−3.519305
H	−0.136064	−4.028897	−4.419086
H	−0.146182	−4.431094	−2.677917
C	−3.479671	0.675233	−0.309693
C	−4.084968	−0.062156	−1.331638
C	−4.264352	1.567272	0.428597
C	−5.431016	0.112009	−1.619954
H	−3.499236	−0.783913	−1.885983
C	−5.610926	1.739650	0.139157
H	−3.808521	2.134351	1.234484
C	−6.198359	1.014205	−0.890516
H	−5.886826	−0.469034	−2.414123
H	−6.202105	2.439544	0.719454
H	−7.250702	1.143885	−1.117757

trans-3-E

charge, 2S+1 = 0, 1
--- OPT at ωB97X-D/6-311G(d,p) in SMD ---
E(ele) = -1611.79705698 a.u.
Correction to G = 0.397900 a.u.(RRHO)
Correction to G = 0.405860 a.u.(quasi-RRHO)
--- SP at ωB97X-D/def2-TZVP in SMD ---
E(ele) = -1611.96198098 a.u.

P	1.044459	−0.180680	0.062483
C	1.816597	1.215000	−0.793680
C	1.946298	1.169239	−2.184589
C	2.216075	2.351484	−0.091101
C	2.471112	2.258729	−2.863928
C	2.745801	3.435372	−0.778781
C	2.871328	3.390435	−2.161788
H	1.636263	0.287331	−2.733939
H	2.116508	2.394413	0.987156
H	2.567246	2.223264	−3.942664
H	3.058817	4.316412	−0.231002
H	3.283138	4.239672	−2.695131
C	1.777972	−1.665181	−0.679856
C	0.977389	−2.730930	−1.090351
C	3.161562	−1.715019	−0.880650
C	1.563764	−3.847623	−1.673653
C	3.738364	−2.835057	−1.459760
C	2.939387	−3.903744	−1.852770
H	−0.097971	−2.687958	−0.963631
H	3.787111	−0.872806	−0.604873
H	0.937731	−4.672634	−1.992365
H	4.810546	−2.868851	−1.613705
H	3.391173	−4.776976	−2.309479
C	1.470447	−0.066342	1.819136
C	0.592108	0.637137	2.648477
C	2.642758	−0.610204	2.340450
C	0.903398	0.807550	3.989619
C	2.943083	−0.439112	3.685789
C	2.077332	0.271657	4.507600
H	−0.336961	1.031164	2.250380
H	3.316894	−1.179797	1.712241
H	0.218899	1.348584	4.632100
H	3.851471	−0.868652	4.091712
H	2.312656	0.399968	5.558143
C	−0.763117	−0.192050	−0.079167
C	−1.279498	0.794560	−0.876291
H	−0.591082	1.542966	−1.253818
C	−1.349121	−1.176407	0.762087
H	−0.750997	−2.019104	1.088318
C	−2.507635	−0.922434	1.543190
O	−3.190296	0.091744	1.580092
O	−2.809168	−1.995906	2.350444
C	−3.907133	−1.803451	3.222594
H	−3.724789	−0.986677	3.927844
H	−4.825765	−1.583128	2.671764
H	−4.022648	−2.739563	3.770569
C	−2.637328	0.965809	−1.377853
C	−3.639636	−0.012305	−1.297720
C	−2.939731	2.153177	−2.065303
C	−4.877998	0.190634	−1.889678
H	−3.435695	−0.944510	−0.790083
C	−4.185223	2.363563	−2.635295
H	−2.176312	2.921591	−2.147731
C	−5.165262	1.379524	−2.550605
H	−5.633042	−0.585441	−1.820167
H	−4.391916	3.296117	−3.149901
H	−6.142240	1.538743	−2.993835

trans-ts3-Z

charge, 2S+1 = 0, 1
--- OPT at ωB97X-D/6-311G(d,p) in SMD ---
E(ele) = -1611.77706160 a.u.
Correction to G = 0.393370 a.u.(RRHO)
Correction to G = 0.402494 a.u.(quasi-RRHO)
imaginary frequency = 216i
--- SP at ωB97X-D/def2-TZVP in SMD ---
E(ele) = -1611.93691935 a.u.

P	0.824036	0.218743	0.387700
C	2.132429	0.695558	−0.779075
C	2.820499	1.907611	−0.697604
C	2.384636	−0.165464	−1.852444
C	3.749962	2.248974	−1.671873
C	3.316828	0.181096	−2.821047
C	3.998346	1.389255	−2.735324
H	2.643075	2.583605	0.130828
H	1.863324	−1.114507	−1.916799
H	4.284706	3.189099	−1.595601
H	3.507089	−0.495504	−3.646505
H	4.723183	1.660047	−3.494869
C	1.350271	−1.385125	1.070325
C	2.695534	−1.723555	1.234428
C	0.359576	−2.288990	1.453090
C	3.041318	−2.949609	1.786369
C	0.710178	−3.511538	2.011657
C	2.049211	−3.841983	2.179048
H	3.471353	−1.031301	0.925813
H	−0.684715	−2.045539	1.293758
H	4.086889	−3.209359	1.908482
H	−0.064622	−4.211776	2.301964
H	2.321691	−4.800191	2.607372
C	0.935609	1.395533	1.781142
C	0.360733	2.659567	1.608400
C	1.524767	1.076581	3.005272
C	0.400961	3.595564	2.632452
C	1.552112	2.012633	4.032284
C	0.996290	3.272385	3.847091
H	−0.124284	2.908344	0.669682
H	1.960953	0.096639	3.160792
H	−0.042795	4.573634	2.485250
H	2.011192	1.754637	4.980022
H	1.020072	3.999995	4.650601
C	−1.243706	0.255179	−0.746265
C	−2.108489	0.457939	0.238199
H	−1.752138	0.674360	1.240466
C	−1.095566	0.121381	−2.091784
H	−1.207444	0.985607	−2.736831
C	−0.639359	−1.117864	−2.656239
O	−0.308701	−2.115117	−2.035069
O	−0.586220	−1.070776	−4.010348
C	−0.124224	−2.253622	−4.648361
H	0.907558	−2.479718	−4.365737
H	−0.752152	−3.111907	−4.398019
H	−0.177140	−2.053593	−5.718089
C	−3.578199	0.406652	0.109139
C	−4.343564	0.665644	1.251379
C	−4.244119	0.103932	−1.083763
C	−5.730717	0.627152	1.207029
H	−3.841107	0.900016	2.184919
C	−5.629723	0.060749	−1.124947
H	−3.666232	−0.100829	−1.977163
C	−6.380121	0.323709	0.017036
H	−6.304035	0.833140	2.104249
H	−6.128835	−0.178931	−2.057531
H	−7.463268	0.291694	−0.021851

trans-3-Z

charge, 2S+1 = 0, 1
--- OPT at ωB97X-D/6-311G(d,p) in SMD ---
E(ele) = -1611.80704317 a.u.
Correction to G = 0.400116 a.u.(RRHO)
Correction to G = 0.406940 a.u.(quasi-RRHO)
--- SP at ωB97X-D/def2-TZVP in SMD ---
E(ele) = -1611.97088381 a.u.

P	0.736633	-0.034942	0.162192
C	1.849488	0.725878	-1.045682
C	1.343928	1.769824	-1.818948
C	3.175960	0.326474	-1.184612
C	2.173938	2.416720	-2.726729
C	3.998208	0.980268	-2.090280
C	3.499692	2.025796	-2.860398
H	0.305684	2.068108	-1.724892
H	3.562028	-0.509513	-0.614780
H	1.777653	3.222396	-3.333757
H	5.028653	0.663767	-2.203590
H	4.144459	2.529631	-3.571660
C	1.256946	-1.655844	0.783718
C	2.433362	-1.761030	1.526361
C	0.448194	-2.773617	0.591078
C	2.814168	-2.990515	2.047778
C	0.823321	-3.993406	1.135255
C	2.008424	-4.105572	1.853698
H	3.045292	-0.885823	1.715821
H	-0.467274	-2.688434	0.018221
H	3.733775	-3.071870	2.615457
H	0.190420	-4.860986	0.989642
H	2.301284	-5.063413	2.268846
C	0.879469	1.015111	1.652181
C	1.366656	2.319559	1.577259
C	0.466481	0.505600	2.887629
C	1.434607	3.105236	2.721515
C	0.530949	1.296061	4.025236
C	1.015192	2.597107	3.943656
H	1.698594	2.728784	0.631125
H	0.087689	-0.507293	2.960482
H	1.817845	4.116769	2.653613
H	0.203223	0.893948	4.976764
H	1.067802	3.212775	4.834536
C	-0.999178	0.006626	-0.421861
C	-1.858703	0.643533	0.437491
H	-1.447390	1.159211	1.293937
C	-1.269067	-0.535069	-1.702548
H	-2.211582	-0.310192	-2.179108
C	-0.313000	-1.257318	-2.438548
O	0.808563	-1.592011	-2.037681
O	-0.720609	-1.585501	-3.693369
C	0.224069	-2.299209	-4.475467
H	1.133589	-1.713738	-4.634995
H	0.497689	-3.247783	-4.005791
H	-0.264588	-2.488613	-5.431317
C	-3.311221	0.768737	0.331912
C	-3.938147	1.806538	1.044120
C	-4.138295	-0.114536	-0.380390
C	-5.313572	1.972104	1.025356
H	-3.323246	2.494082	1.617425
C	-5.517591	0.047604	-0.389807
H	-3.702044	-0.953117	-0.907142
C	-6.115351	1.093985	0.302082
H	-5.764017	2.789241	1.579000
H	-6.130779	-0.655350	-0.943969
H	-7.191981	1.221258	0.283731

trans-ts3-int

charge, 2S+1 = 0, 1
--- OPT at ωB97X-D/6-311G(d,p) in SMD ---
E(ele) = -1611.78062689 a.u.
Correction to G = 0.395226 a.u.(RRHO)
Correction to G = 0.403456 a.u.(quasi-RRHO)
imaginary frequency = 208i
--- SP at ωB97X-D/def2-TZVP in SMD ---
E(ele) = -1611.93938859 a.u.

P	0.909730	0.284716	-0.025330
C	2.073793	1.318928	-0.982637
C	1.560014	2.318406	-1.810017
C	3.457702	1.141925	-0.899826
C	2.416888	3.140869	-2.531735
C	4.310585	1.961934	-1.625967
C	3.791573	2.963684	-2.439667
H	0.486997	2.448346	-1.900305
H	3.866747	0.360051	-0.269512
H	2.008406	3.913572	-3.172989
H	5.382917	1.817406	-1.557429
H	4.459918	3.601505	-3.007267
C	1.742546	-1.320315	0.105986
C	1.842461	-2.074359	-1.065953
C	2.191533	-1.859923	1.311393
C	2.386383	-3.352181	-1.030814
C	2.740689	-3.133847	1.340250
C	2.834202	-3.883462	0.171767
H	1.493914	-1.664899	-2.007777
H	2.103810	-1.290843	2.229363
H	2.451523	-3.933441	-1.943306
H	3.091754	-3.545270	2.279902
H	3.255322	-4.882354	0.200377
C	0.857405	1.001313	1.644308
C	1.645953	2.081173	2.044331
C	-0.078877	0.457036	2.531213
C	1.510711	2.601324	3.325837
C	-0.197925	0.975587	3.813493
C	0.593417	2.047928	4.211401
H	2.365755	2.516671	1.360427
H	-0.698650	-0.376086	2.210037
H	2.125852	3.439957	3.632446
H	-0.918831	0.545899	4.499765
H	0.491383	2.456114	5.210883
C	-1.224714	-0.188017	-0.853415
C	-1.850031	0.977643	-0.758959
H	-1.296270	1.866518	-0.473869
C	-1.339040	-1.500758	-1.194807
H	-1.322217	-1.805740	-2.234931
C	-1.260087	-2.504797	-0.166656
O	-1.195441	-2.298989	1.034733
O	-1.253824	-3.759553	-0.675031
C	-1.095920	-4.802799	0.277947
H	-0.162440	-4.685909	0.833729
H	-1.929228	-4.824136	0.984680
H	-1.074182	-5.728393	-0.296658
C	-3.294661	1.196048	-0.966946
C	-4.187762	0.185418	-1.339377
C	-3.795606	2.487594	-0.771013
C	-5.536436	0.463528	-1.504583
H	-3.816733	-0.820887	-1.492478
C	-5.145144	2.765330	-0.941161
H	-3.116193	3.281962	-0.477564
C	-6.022136	1.752667	-1.309021
H	-6.214598	-0.333238	-1.790038
H	-5.511527	3.773832	-0.783799
H	-7.077057	1.964970	-1.442553

trans-3-int

charge, 2S+1 = 0, 1
--- OPT at ωB97X-D/6-311G(d,p) in SMD ---
E(ele) = -1611.79936811 a.u.
Correction to G = 0.400039 a.u.(RRHO)
Correction to G = 0.406756 a.u.(quasi-RRHO)
--- SP at ωB97X-D/def2-TZVP in SMD ---
E(ele) = -1611.96221154 a.u.

P	0.715049	0.077074	0.191700
C	1.663280	1.200785	-0.893706
C	1.147199	1.730681	-2.073814
C	3.002033	1.434922	-0.559810
C	1.953892	2.511166	-2.895061
C	3.797597	2.222469	-1.376581
C	3.272486	2.764456	-2.545619
H	0.121032	1.533261	-2.357295
H	3.427193	0.994271	0.335672
H	1.544515	2.917625	-3.812498
H	4.831131	2.404352	-1.105938
H	3.896796	3.375550	-3.187567
C	1.724879	-1.423698	0.124454
C	1.850807	-2.066301	-1.107029
C	2.407371	-1.900333	1.239806
C	2.634891	-3.206957	-1.208293
C	3.203074	-3.031267	1.125395
C	3.310265	-3.689929	-0.093877
H	1.328737	-1.689952	-1.978555
H	2.307887	-1.407618	2.199182
H	2.718776	-3.716114	-2.161268
H	3.731325	-3.404657	1.995048
H	3.924809	-4.579399	-0.176462
C	0.703854	0.768215	1.865063
C	1.212215	2.035411	2.148864
C	0.080767	0.017109	2.867618
C	1.120204	2.540197	3.440109
C	0.001137	0.530040	4.153938
C	0.521546	1.787349	4.441873
H	1.671315	2.635119	1.372509
H	-0.337965	-0.953687	2.619494
H	1.513748	3.526004	3.659105
H	-0.478829	-0.052196	4.931896
H	0.451418	2.184961	5.448176
C	-0.998722	-0.103613	-0.436307
C	-1.605577	1.110303	-0.569872
H	-1.028262	2.000889	-0.331566
C	-1.422559	-1.406383	-0.818706
H	-2.064663	-1.530402	-1.679560
C	-1.164888	-2.513403	0.011271
O	-0.652362	-2.481434	1.136957
O	-1.544415	-3.705060	-0.533904
C	-1.302459	-4.846770	0.270282
H	-0.233996	-4.979213	0.463050
H	-1.824123	-4.782571	1.229165
H	-1.681724	-5.696906	-0.297747
C	-2.973415	1.412226	-0.987828
C	-3.997281	0.459442	-1.112409
C	-3.303551	2.755625	-1.235949
C	-5.284117	0.840995	-1.466597
H	-3.786827	-0.581561	-0.908437
C	-4.586356	3.131317	-1.602111
H	-2.532684	3.514114	-1.135118
C	-5.587759	2.173257	-1.722085
H	-6.057969	0.084807	-1.546229
H	-4.806983	4.176480	-1.791496
H	-6.592667	2.462251	-2.009051

trans-ts3-rotE

charge, 2S+1 = 0, 1
--- OPT at ωB97X-D/6-311G(d,p) in SMD ---
E(ele) = -1611.78588785 a.u.
Correction to G = 0.397894 a.u.(RRHO)
Correction to G = 0.405336 a.u.(quasi-RRHO)
imaginary frequency = 78i
--- SP at ωB97X-D/def2-TZVP in SMD ---
E(ele) = -1611.94967237 a.u.

P	0.828735	0.382608	-0.487202
C	1.471783	1.605369	-1.664301
C	0.600383	2.483206	-2.307699
C	2.845442	1.674356	-1.917252
C	1.102792	3.433133	-3.188853
C	3.339048	2.626154	-2.797174
C	2.468211	3.506844	-3.430983
H	-0.467454	2.418039	-2.134711
H	3.525471	0.980654	-1.434877
H	0.422289	4.110743	-3.690897
H	4.403821	2.676580	-2.992816
H	2.856294	4.246961	-4.121550
C	1.738283	-1.149697	-0.785036
C	1.874238	-1.597837	-2.101275
C	2.213076	-1.925287	0.269043
C	2.487478	-2.813792	-2.356413
C	2.824924	-3.144925	0.005294
C	2.961037	-3.588757	-1.302610
H	1.497926	-1.001871	-2.925466
H	2.102109	-1.592530	1.293482
H	2.587483	-3.161640	-3.377768
H	3.190149	-3.748701	0.827678
H	3.434751	-4.542929	-1.503989
C	1.179623	0.953066	1.197307
C	2.084466	1.980928	1.461399
C	0.505068	0.312802	2.243804
C	2.321633	2.365733	2.774876
C	0.760522	0.701058	3.551794
C	1.664096	1.724439	3.817814
H	2.595130	2.490059	0.652251
H	-0.214148	-0.478208	2.026869
H	3.018711	3.169649	2.981326
H	0.241885	0.205906	4.364455
H	1.851057	2.028249	4.841798
C	-0.979608	0.025358	-0.648695
C	-1.745101	1.021559	-0.163419
H	-1.248971	1.920996	0.202704
C	-1.318133	-1.319527	-1.071646
H	-1.085495	-1.636949	-2.081003
C	-1.382380	-2.297846	-0.071894
O	-1.356299	-2.132531	1.157334
O	-1.480422	-3.576795	-0.578851
C	-1.459855	-4.611369	0.381809

H	-0.517028	-4.628842	0.938981
H	-2.280158	-4.520382	1.099722
H	-1.566117	-5.542714	-0.177536
C	-3.207130	1.098237	-0.063157
C	-4.058138	-0.011177	-0.139249
C	-3.776421	2.365158	0.125739
C	-5.433935	0.156557	-0.060427
H	-3.626453	-0.993812	-0.265391
C	-5.151134	2.528930	0.205298
H	-3.127388	3.232312	0.205726
C	-5.986253	1.421207	0.109004
H	-6.079894	-0.712423	-0.121751
H	-5.570802	3.518824	0.346912
H	-7.061946	1.542499	0.175805

trans-ts3-rotZ

charge, 2S+1 = 0, 1
--- OPT at ωB97X-D/6-311G(d,p) in SMD ---
E(ele) = -1611.79414029 a.u.
Correction to G = 0.400244 a.u.(RRHO)
Correction to G = 0.406795 a.u.(quasi-RRHO)
imaginary frequency = 77i
--- SP at ωB97X-D/def2-TZVP in SMD ---
E(ele) = -1611.95679887 a.u.

P	0.741018	0.125726	0.072602
C	1.733092	1.267779	-0.970858
C	1.299040	2.565720	-1.261381
C	2.991868	0.866339	-1.425518
C	2.092926	3.426081	-2.003881
C	3.783410	1.731705	-2.171705
C	3.336203	3.010668	-2.466340
H	0.344072	2.926115	-0.908472
H	3.368292	-0.122601	-1.201709
H	1.735675	4.426040	-2.220574
H	4.753862	1.397252	-2.519507
H	3.953343	3.684271	-3.049874
C	1.679208	-1.419091	0.052657
C	1.737686	-2.165031	-1.125953
C	2.360667	-1.846080	1.188956
C	2.460895	-3.349762	-1.151610
C	3.089521	-3.025834	1.151081
C	3.135125	-3.780652	-0.015271
H	1.210435	-1.832604	-2.011115
H	2.305498	-1.276098	2.108526
H	2.493426	-3.936518	-2.062122
H	3.612626	-3.361451	2.038947
H	3.697819	-4.707268	-0.038623
C	0.749907	0.825022	1.740639
C	1.666151	1.807327	2.118255
C	-0.182650	0.335581	2.659131
C	1.644848	2.303877	3.415122
C	-0.193676	0.839277	3.952004
C	0.715009	1.822301	4.328914
H	2.391089	2.187801	1.408008
H	-0.864306	-0.452229	2.356647
H	2.353968	3.068792	3.709499
H	-0.915289	0.461130	4.666659
H	0.698774	2.214341	5.339774
C	-0.989047	-0.091092	-0.544565
C	-1.679137	1.075195	-0.644876
H	-1.181096	1.998179	-0.372439
C	-1.363647	-1.413808	-0.928029
H	-1.942630	-1.569038	-1.827781
C	-1.172174	-2.474983	-0.027144
O	-0.743221	-2.380322	1.129977
O	-1.513294	-3.697344	-0.535140
C	-1.350915	-4.790022	0.351576
H	-0.304221	-4.917439	0.642284
H	-1.950063	-4.667071	1.258255
H	-1.689770	-5.670914	-0.195154
C	-3.076668	1.287793	-1.026187
C	-4.032685	0.269552	-1.166725
C	-3.506903	2.612266	-1.212108
C	-5.352030	0.572180	-1.474415
H	-3.741615	-0.760518	-1.014211
C	-4.821669	2.910228	-1.533784
H	-2.789328	3.419993	-1.100280
C	-5.755324	1.888207	-1.668571
H	-6.072256	-0.233734	-1.567762
H	-5.119842	3.943333	-1.677049
H	-6.785045	2.115323	-1.921238

trans-ts3-rot

charge, 2S+1 = 0, 1
--- OPT at ωB97X-D/6-311G(d,p) in SMD ---
E(ele) = -1611.78578524 a.u.
Correction to G = 0.398047 a.u.(RRHO)
Correction to G = 0.405353 a.u.(quasi-RRHO)
imaginary frequency = 137i
--- SP at ωB97X-D/def2-TZVP in SMD ---
E(ele) = -1611.94992878 a.u.

P	-0.271552	-0.342892	0.200372
C	-1.755320	-1.382950	0.325994
C	-3.040872	-0.870420	0.157448
C	-1.582560	-2.734436	0.645483
C	-4.142613	-1.707308	0.290381
C	-2.686206	-3.564523	0.769980
C	-3.967075	-3.051480	0.591238
H	-3.188583	0.178842	-0.068049
H	-0.586663	-3.132817	0.807956
H	-5.139979	-1.303363	0.161192
H	-2.546351	-4.610778	1.015368
H	-4.829091	-3.700670	0.694690
C	0.635503	-0.794667	-1.292800
C	0.328121	-1.927521	-2.044747
C	1.702705	0.028758	-1.666560
C	1.091823	-2.241448	-3.162080
C	2.460379	-0.294727	-2.782585
C	2.158846	-1.429744	-3.526585
H	-0.505385	-2.563266	-1.771241
H	1.929764	0.923114	-1.098328
H	0.849843	-3.121073	-3.747166
H	3.290119	0.341373	-3.066772
H	2.754916	-1.679567	-4.397218
C	-0.805225	1.370984	-0.031646
C	-0.665581	2.298507	0.997008
C	-1.381355	1.746147	-1.249819
C	-1.124694	3.597321	0.810876
C	-1.838417	3.042909	-1.424453
C	-1.713846	3.966803	-0.390339
H	-0.147918	2.025934	1.907065
H	-1.464932	1.029290	-2.060121
H	-1.002713	4.324182	1.605146
H	-2.283438	3.335582	-2.368370
H	-2.065100	4.983108	-0.530279
C	0.826020	-0.529800	1.684853
C	0.129109	-0.729428	2.817978
H	-0.955154	-0.810426	2.748932
C	2.254121	-0.409110	1.451995
H	2.816162	-1.291312	1.170979
C	2.850898	0.848032	1.373375
O	2.323813	1.958431	1.528036
O	4.201314	0.776950	1.070396
C	4.869625	2.018542	1.003360
H	4.456880	2.666099	0.222842
H	4.822770	2.560909	1.952926
H	5.910547	1.787085	0.769211
C	0.619857	-0.877642	4.196118
C	1.938718	-0.624991	4.594565
C	-0.304068	-1.286924	5.166467
C	2.313060	-0.792157	5.919856
H	2.656356	-0.306034	3.850272
C	0.073989	-1.455716	6.490661
H	-1.332263	-1.477888	4.873808
C	1.387580	-1.208666	6.871300
H	3.337612	-0.591133	6.212946
H	-0.656483	-1.776870	7.224915
H	1.688273	-1.335840	7.905541

cis-ts3-E

charge, 2S+1 = 0, 1
--- OPT at ωB97X-D/6-311G(d,p) in SMD ---
E(ele) = -1611.77515020 a.u.
Correction to G = 0.398666 a.u.(RRHO)
Correction to G = 0.405161 a.u.(quasi-RRHO)
imaginary frequency = 203i
--- SP at ωB97X-D/def2-TZVP in SMD ---
E(ele) = -1611.93498438 a.u.

P	-0.375533	0.063222	0.243260
C	-1.969210	0.961202	0.295579
C	-1.973017	2.354197	0.351862
C	-3.179087	0.269030	0.381408
C	-3.171532	3.046500	0.480196
C	-4.374397	0.962395	0.505693
C	-4.371947	2.352507	0.554445
H	-1.039004	2.902166	0.289236
H	-3.189264	-0.815074	0.348485
H	-3.164327	4.129760	0.519310
H	-5.309109	0.416716	0.567133
H	-5.306653	2.893267	0.651702
C	-0.352352	-0.705846	1.914606
C	-0.857558	-1.977875	2.188909
C	0.160648	0.064371	2.959178
C	-0.836791	-2.471281	3.488014
C	0.171770	-0.427830	4.256360
C	-0.322789	-1.699139	4.522570
H	-1.267929	-2.589275	1.393867
H	0.569214	1.048497	2.756908
H	-1.228398	-3.461922	3.690670
H	0.578909	0.179325	5.056760
H	-0.307067	-2.088027	5.534561
C	-0.582967	-1.295013	-0.944098
C	-1.422856	-1.154633	-2.049555
C	0.250436	-2.415484	-0.858564
C	-1.430926	-2.120051	-3.050043
C	0.231760	-3.380410	-1.853417
C	-0.605909	-3.231388	-2.954454
H	-2.065928	-0.288629	-2.140321
H	0.920709	-2.532979	-0.014040
H	-2.080871	-1.993111	-3.908177
H	0.882300	-4.243982	-1.776473
H	-0.610460	-3.981378	-3.737479
C	0.855334	1.464628	-0.913733
C	2.014534	1.776268	-0.344599
H	2.547200	2.601979	-0.812270
C	0.192808	1.629478	-2.104760
H	-0.797080	2.064129	-2.156889
C	0.697361	0.965347	-3.283320
O	1.742798	0.358796	-3.407958
O	-0.180476	1.087621	-4.320392
C	0.203679	0.429581	-5.517729
H	0.340476	-0.642428	-5.353378
H	-0.609288	0.597124	-6.224493
H	1.133061	0.842057	-5.919302
C	2.662570	1.136174	0.810268
C	3.115644	1.907347	1.885282
C	2.845849	-0.247575	0.850437
C	3.684666	1.301411	2.996403
H	2.999689	2.986006	1.853444
C	3.419344	-0.853910	1.961425
H	2.557637	-0.837829	-0.011826
C	3.829180	-0.082627	3.040747
H	4.016806	1.908818	3.831187
H	3.547764	-1.930392	1.981851
H	4.271893	-0.554958	3.910476

cis-3-E

charge, 2S+1 = 0, 1
--- OPT at ωB97X-D/6-311G(d,p) in SMD ---
E(ele) = -1611.79618692 a.u.
Correction to G = 0.400757 a.u.(RRHO)
Correction to G = 0.407200 a.u.(quasi-RRHO)
--- SP at ωB97X-D/def2-TZVP in SMD ---
E(ele) = -1611.96007285 a.u.

P	-0.563381	-0.330486	0.040505
C	-1.532569	0.485476	-1.254885
C	-1.222543	1.796013	-1.617413
C	-2.610963	-0.171621	-1.854321
C	-1.972284	2.433922	-2.597060
C	-3.350304	0.471034	-2.836344
C	-3.028515	1.771806	-3.209724
H	-0.402428	2.313772	-1.131921
H	-2.885601	-1.174455	-1.545319
H	-1.729942	3.452444	-2.875797
H	-4.183573	-0.040679	-3.303416
H	-3.610458	2.273362	-3.974625
C	-1.782814	-0.999148	1.211644
C	-2.975969	-0.308178	1.438260
C	-1.524618	-2.183765	1.900851
C	-3.896775	-0.799595	2.351728
C	-2.451501	-2.668874	2.813656
C	-3.635412	-1.978690	3.039888
H	-3.181876	0.615936	0.911330
H	-0.608055	-2.734459	1.728044

```
H     -4.818108   -0.257072    2.527738
H     -2.245528   -3.589222    3.347357
H     -4.356766   -2.360251    3.753534
C      0.350934   -1.756980   -0.601540
C      1.446959   -2.216984    0.130731
C     -0.020277   -2.399372   -1.780781
C      2.170420   -3.308978   -0.325221
C      0.706948   -3.493350   -2.228040
C      1.802742   -3.946116   -1.503986
H      1.754860   -1.702328    1.033566
H     -0.851277   -2.036650   -2.372696
H      3.032612   -3.650446    0.234945
H      0.423731   -3.983814   -3.151970
H      2.377143   -4.792196   -1.863501
C      0.534737    0.881877    0.832324
C      1.798709    1.139795    0.356831
H      2.350143    1.833663    0.982476
C     -0.124318    1.524877    1.913150
H     -1.035342    1.118390    2.322115
C      0.375090    2.689680    2.554402
O      1.404639    3.312540    2.321704
O     -0.474863    3.109438    3.550421
C     -0.060722    4.266592    4.252565
H     -0.826258    4.448446    5.007985
H      0.907808    4.118508    4.739016
H      0.015398    5.134774    3.591176
C      2.551346    0.629098   -0.788240
C      3.931003    0.411837   -0.617310
C      2.019928    0.359012   -2.056874
C      4.717257   -0.104812   -1.633754
H      4.377214    0.639393    0.345686
C      2.804701   -0.169062   -3.074469
H      0.981803    0.579917   -2.269427
C      4.155828   -0.413733   -2.870153
H      5.775441   -0.271184   -1.460785
H      2.354619   -0.376268   -4.039796
H      4.768114   -0.822393   -3.666202
```

cis-ts3-Z

```
charge, 2S+1    = 0, 1
--- OPT at ωB97X-D/6-311G(d,p) in SMD ---
E(ele)          = -1611.77711702 a.u.
Correction to G = 0.399179 a.u.(RRHO)
Correction to G = 0.405257 a.u.(quasi-RRHO)
imaginary frequency = 223i
--- SP at ωB97X-D/def2-TZVP in SMD ---
E(ele)          = -1611.93471447 a.u.
P     -0.262535   -0.033054    0.187551
C     -1.921170    0.616960   -0.163819
C     -2.338414    1.760981    0.521970
C     -2.754354    0.059386   -1.134082
C     -3.573433    2.333908    0.244111
C     -3.988225    0.632544   -1.406359
C     -4.397537    1.772321   -0.721806
H     -1.699484    2.208418    1.273932
H     -2.436764   -0.816107   -1.687325
H     -3.882704    3.226007    0.776078
H     -4.629424    0.190758   -2.160664
H     -5.358493    2.223131   -0.943926
C     -0.413471   -0.848451    1.822838
C      0.635413   -1.656596    2.272402
C     -1.521496   -0.660673    2.651949
C      0.576054   -2.261285    3.519359
C     -1.572242   -1.260390    3.904726
C     -0.525213   -2.060035    4.343341
H      1.496106   -1.832483    1.640240
H     -2.357842   -0.056916    2.323361
H      1.398240   -2.887992    3.846004
H     -2.440807   -1.104850    4.534734
H     -0.568872   -2.528189    5.320324
C      0.007638   -1.375409   -1.015974
C     -0.150688   -2.727290   -0.708613
C      0.399598   -0.998555   -2.303902
C      0.097800   -3.693257   -1.675558
C      0.636165   -1.970516   -3.267393
C      0.495007   -3.317505   -2.953503
H     -0.467465   -3.032527    0.281362
H      0.492669    0.055684   -2.548109
H     -0.024977   -4.742030   -1.429253
H      0.935115   -1.671644   -4.265788
H      0.688765   -4.074381   -3.705592
```

```
C      1.067137    1.815544   -0.153372
C      2.334543    1.502868    0.066156
H      3.039424    2.278637   -0.233531
C      0.317062    2.909560   -0.511282
H      0.146177    3.702802    0.206225
C     -0.410678    2.921983   -1.741946
O     -0.367948    2.068431   -2.617785
O     -1.203463    4.016518   -1.863638
C     -2.016639    4.053883   -3.027388
H     -2.603080    4.969351   -2.950819
H     -1.410775    4.077498   -3.936883
H     -2.682270    3.187972   -3.069080
C      2.952379    0.288499    0.652682
C      3.342376    0.280682    1.993381
C      3.244362   -0.822218   -0.141155
C      3.996347   -0.819324    2.533293
H      3.123416    1.141841    2.615228
C      3.886829   -1.928748    0.402668
H      2.961622   -0.819807   -1.187103
C      4.266375   -1.929412    1.740348
H      4.288382   -0.812119    3.577513
H      4.094968   -2.788539   -0.224282
H      4.773751   -2.789963    2.162132
```

cis-3-Z

```
charge, 2S+1    = 0, 1
--- OPT at ωB97X-D/6-311G(d,p) in SMD ---
E(ele)          = -1611.79483582 a.u.
Correction to G = 0.400603 a.u.(RRHO)
Correction to G = 0.406511 a.u.(quasi-RRHO)
--- SP at ωB97X-D/def2-TZVP in SMD ---
E(ele)          = -1611.95764151 a.u.
P     -0.366651    0.165377    0.182272
C     -1.547241    1.415750   -0.407100
C     -2.693435    0.988920   -1.078664
C     -1.280177    2.776765   -0.279630
C     -3.586205    1.922326   -1.588296
C     -2.164177    3.703004   -0.813834
C     -3.320818    3.279535   -1.458139
H     -2.886390   -0.068231   -1.224241
H     -0.386221    3.109137    0.234554
H     -4.480499    1.585918   -2.099865
H     -1.950270    4.761197   -0.718110
H     -4.012229    4.008287   -1.866137
C     -0.973161   -0.956007    1.465057
C     -2.330596   -1.194525    1.663347
C     -0.020972   -1.639469    2.217656
C     -2.731446   -2.126420    2.608555
C     -0.431219   -2.569914    3.165261
C     -1.783740   -2.816726    3.357885
H     -3.074400   -0.638998    1.105686
H      1.035211   -1.434451    2.082233
H     -3.788187   -2.306001    2.769489
H      0.310202   -3.094352    3.756761
H     -2.102679   -3.540310    4.099528
C     -0.211581   -0.874866   -1.306682
C     -0.453138   -2.245644   -1.259678
C      0.096545   -0.266013   -2.525711
C     -0.363838   -3.005384   -2.420328
C      0.203755   -1.030811   -3.674370
C     -0.026935   -2.402283   -3.623142
H     -0.702598   -2.729677   -0.323518
H      0.272779    0.802550   -2.571777
H     -0.552748   -4.071863   -2.377949
H      0.468766   -0.556337   -4.611670
H      0.055109   -2.998358   -4.525020
C      1.202459    0.954368    0.708363
C      2.334764    0.911668   -0.068567
H      3.075651    1.659639    0.210924
C      1.145609    1.724761    1.904181
H      2.086500    2.107007    2.280099
C     -0.006976    1.961603    2.673198
O     -1.160531    1.606162    2.408625
O      0.249713    2.654492    3.820482
C     -0.875690    2.900050    4.646985
H     -1.325598    1.967563    4.999025
H     -0.501334    3.470962    5.497105
H     -1.641099    3.478395    4.122257
C      2.818263   -0.026176   -1.087628
C      2.732142   -1.415835   -0.941914
C      3.473252    0.468261   -2.224887
```

```
C      3.212694   -2.272746   -1.922442
H      2.284894   -1.826078   -0.043250
C      3.967247   -0.386146   -3.199196
H      3.573968    1.542275   -2.346995
C      3.827870   -1.763444   -3.059810
H      3.115821   -3.345059   -1.790576
H      4.459774    0.024081   -4.074545
H      4.208450   -2.433152   -3.822959
```

cis-ts3-int

```
charge, 2S+1    = 0, 1
--- OPT at ωB97X-D/6-311G(d,p) in SMD ---
E(ele)          = -1611.77693949 a.u.
Correction to G = 0.399874 a.u.(RRHO)
Correction to G = 0.405669 a.u.(quasi-RRHO)
imaginary frequency = 222i
--- SP at ωB97X-D/def2-TZVP in SMD ---
E(ele)          = -1611.93480819 a.u.
P     -0.049329   -0.363587    0.028149
C     -0.463254    0.725851   -1.363206
C     -0.832510    2.034875   -1.030070
C     -0.289456    0.376075   -2.701524
C     -1.064441    2.963983   -2.034079
C     -0.516742    1.315942   -3.699317
C     -0.909703    2.606596   -3.369489
H     -0.918889    2.319105    0.015977
H      0.042147   -0.618712   -2.972328
H     -1.355614    3.974477   -1.770044
H     -0.376171    1.037520   -4.737726
H     -1.084437    3.337295   -4.151497
C     -1.560604   -0.566932    1.023259
C     -2.791436    0.005379    0.703587
C     -1.439497   -1.327810    2.187874
C     -3.882269   -0.178378    1.541033
C     -2.530962   -1.499127    3.029945
C     -3.752688   -0.922841    2.709048
H     -2.901285    0.601526   -0.193978
H     -0.491837   -1.788622    2.442601
H     -4.836602    0.266654    1.282606
H     -2.421837   -2.080227    3.938290
H     -4.605182   -1.055211    3.366102
C      0.170161   -2.073067   -0.590578
C     -0.747364   -2.656148   -1.469034
C      1.210058   -2.847191   -0.078620
C     -0.596293   -3.978775   -1.859728
C      1.352033   -4.176507   -0.461020
C      0.455857   -4.739522   -1.359443
H     -1.586675   -2.078772   -1.840811
H      1.906542   -2.415178    0.631742
H     -1.308523   -4.420627   -2.547369
H      2.163085   -4.769900   -0.054581
H      0.568467   -5.774402   -1.662558
C      1.423613    0.678961    1.456994
C      2.386612    1.207266    0.715121
H      2.995503    1.940915    1.242526
C      0.955077    0.683824    2.746692
H      1.365576    0.015215    3.493319
C     -0.210403    1.457411    3.056235
O     -0.800389    2.212252    2.295315
O     -0.627260    1.278502    4.335563
C     -1.815867    1.970479    4.689914
H     -2.647224    1.679632    4.043015
H     -2.029358    1.686315    5.720308
H     -1.680815    3.053355    4.625587
C      2.705167    1.026601   -0.717212
C      2.660360    2.140154   -1.562628
C      3.061086   -0.208353   -1.254540
C      2.921062    2.008277   -2.917256
H      2.388251    3.106869   -1.152986
C      3.322801   -0.342171   -2.614437
H      3.151221   -1.066990   -0.602818
C      3.246847    0.763470   -3.449283
H      2.859820    2.876666   -3.563443
H      3.590429   -1.313458   -3.015664
H      3.446741    0.660810   -4.510125
```

cis-3-int

```
charge, 2S+1    = 0, 1
--- OPT at ωB97X-D/6-311G(d,p) in SMD ---
E(ele)          = -1611.79143191 a.u.
```

```
Correction to G = 0.400660 a.u.(RRHO)
Correction to G = 0.406766 a.u.(quasi-RRHO)
--- SP at ωB97X-D/def2-TZVP in SMD ---
E(ele)          = -1611.95320490 a.u.
 P      0.135822    0.195609   -0.108948
  C      1.155474    1.499143   -0.858518
  C      0.778484    2.826820   -0.657688
  C      2.237091    1.195769   -1.679836
  C      1.509730    3.846817   -1.249990
  C      2.952907    2.220703   -2.282959
  C      2.596792    3.545465   -2.061688
  H     -0.070229    3.066114   -0.029119
  H      2.537796    0.167621   -1.837073
  H      1.225530    4.878388   -1.077908
  H      3.798658    1.981303   -2.917132
  H      3.164258    4.344305   -2.526042
  C     -1.470332    0.526629   -0.907039
  C     -2.570692    0.980294   -0.186868
  C     -1.538616    0.463151   -2.303292
  C     -3.743879    1.321327   -0.849794
  C     -2.713766    0.793978   -2.958741
  C     -3.821370    1.217651   -2.230926
  H     -2.509996    1.077941    0.888738
  H     -0.669307    0.170927   -2.882305
  H     -4.596960    1.669933   -0.279503
  H     -2.760856    0.733127   -4.039746
  H     -4.739072    1.480089   -2.745048
  C      0.766027   -1.417434   -0.645391
  C      0.042785   -2.267671   -1.480989
  C      1.997349   -1.823731   -0.121844
  C      0.558221   -3.514975   -1.805904
  C      2.500532   -3.074830   -0.451687
  C      1.785113   -3.919056   -1.293323
  H     -0.933554   -1.983597   -1.851546
  H      2.538207   -1.153113    0.539500
  H     -0.008953   -4.176128   -2.450816
  H      3.453665   -3.390808   -0.043782
  H      2.181084   -4.896663   -1.544949
  C     -0.042283    0.342922    1.720231
  C     -0.965592   -0.406875    2.372613
  H     -1.056351   -0.176856    3.433703
  C      0.697874    1.427315    2.303073
  H      0.249764    1.996444    3.107270
  C      2.068585    1.550793    2.040813
  O      2.748616    0.794176    1.330500
  O      2.655267    2.629263    2.641181
  C      4.033708    2.802518    2.366669
  H      4.621066    1.933365    2.675775
  H      4.209702    2.979953    1.301503
  H      4.344209    3.677188    2.939703
  C     -1.881870   -1.468760    1.896919
  C     -3.266863   -1.319510    2.045693
  C     -1.405053   -2.679172    1.387945
  C     -4.143475   -2.317976    1.648313
  H     -3.657481   -0.401280    2.472894
  C     -2.281971   -3.680609    0.985424
  H     -0.336685   -2.845141    1.326330
  C     -3.653429   -3.501339    1.104511
  H     -5.212209   -2.173750    1.763833
  H     -1.885478   -4.609201    0.589310
  H     -4.336919   -4.283074    0.792099
```

cis-ts3-rotE

```
charge, 2S+1    = 0, 1
--- OPT at ωB97X-D/6-311G(d,p) in SMD ---
E(ele)          = -1611.78142594 a.u.
Correction to G = 0.401017 a.u.(RRHO)
Correction to G = 0.406849 a.u.(quasi-RRHO)
imaginary frequency = 52i
--- SP at ωB97X-D/def2-TZVP in SMD ---
E(ele)          = -1611.94407051 a.u.
 P      0.159851   -0.766927    0.170399
  C     -0.413867    0.203097   -1.248538
  C     -0.922979    1.476132   -0.965929
  C     -0.299957   -0.233888   -2.566093
  C     -1.363518    2.278385   -2.008198
  C     -0.726964    0.588317   -3.600990
  C     -1.270204    1.835869   -3.323007
  H     -0.924198    1.855216    0.057296
  H      0.144385   -1.194226   -2.796133
  H     -1.758804    3.263722   -1.789698
  H     -0.625648    0.252941   -4.626585
  H     -1.603098    2.473359   -4.134519
  C     -1.282803   -1.247882    1.163681
  C     -2.489508   -0.555689    1.103062
  C     -1.139355   -2.315495    2.053517
  C     -3.539946   -0.923004    1.935202
  C     -2.190959   -2.676052    2.880731
  C     -3.392194   -1.977826    2.824533
  H     -2.621749    0.270863    0.417703
  H     -0.205975   -2.864427    2.107200
  H     -4.474720   -0.376941    1.886224
  H     -2.070493   -3.500820    3.573281
  H     -4.212916   -2.258064    3.475047
  C      0.888514   -2.348477   -0.336042
  C      0.157832   -3.239800   -1.126785
  C      2.129275   -2.727039    0.173371
  C      0.690744   -4.481034   -1.440299
  C      2.653121   -3.976579   -0.136185
  C      1.939680   -4.847818   -0.948660
  H     -0.829621   -2.971484   -1.486039
  H      2.681515   -2.050160    0.815871
  H      0.125845   -5.167341   -2.060232
  H      3.617791   -4.267655    0.262521
  H      2.351041   -5.821036   -1.191268
  C      1.251814    0.286377    1.226992
  C      2.186725    1.035251    0.617925
  H      2.705255    1.735710    1.269806
  C      0.803011    0.456772    2.597882
  H      0.758915   -0.396514    3.263862
  C     -0.064330    1.527075    2.859522
  O     -0.418676    2.439382    2.097398
  O     -0.574818    1.486900    4.141716
  C     -1.523330    2.486052    4.447981
  H     -2.416291    2.406709    3.818579
  H     -1.802000    2.326017    5.491315
  H     -1.110866    3.492496    4.330801
  C      2.519566    1.123293   -0.824856
  C      2.201881    2.296859   -1.515973
  C      3.144357    0.087911   -1.518249
  C      2.457598    2.409097   -2.873540
  H      1.720388    3.108800   -0.981931
  C      3.409485    0.203661   -2.879136
  H      3.435707   -0.812684   -0.993584
  C      3.058419    1.359042   -3.562273
  H      2.183703    3.317572   -3.398338
  H      3.894703   -0.613469   -3.401742
  H      3.258056    1.447701   -4.624419
```

cis-ts3-rotZ

```
charge, 2S+1    = 0, 1
--- OPT at ωB97X-D/6-311G(d,p) in SMD ---
E(ele)          = -1611.78645584 a.u.
Correction to G = 0.401952 a.u.(RRHO)
Correction to G = 0.407243 a.u.(quasi-RRHO)
imaginary frequency = 44i
--- SP at ωB97X-D/def2-TZVP in SMD ---
E(ele)          = -1611.94770153 a.u.
 P      0.075694    0.280339   -0.183819
  C      1.188354    1.530695   -0.882392
  C      0.954134    2.882102   -0.622299
  C      2.223345    1.152474   -1.732808
  C      1.777363    3.844205   -1.190710
  C      3.036158    2.121155   -2.305337
  C      2.819139    3.465497   -2.029369
  H      0.144774    3.179963    0.030885
  H      2.413097    0.105799   -1.935506
  H      1.602807    4.891673   -0.974473
  H      3.847597    1.821508   -2.958316
  H      3.461601    4.219899   -2.469865
  C     -1.493433    0.562554   -1.094177
  C     -2.667398   -0.119877   -0.765830
  C     -1.499791    1.414459   -2.203874
  C     -3.823335    0.064454   -1.510174
  C     -2.659830    1.594446   -2.946475
  C     -3.825377    0.925266   -2.600222
  H     -2.689902   -0.807394    0.063729
  H     -0.604284    1.943189   -2.499854
  H     -4.722271   -0.473126   -1.231549
  H     -2.645310    2.262515   -3.799810
  H     -4.730088    1.070098   -3.179856
  C      0.681884   -1.353542   -0.673746
  C      0.049009   -2.101524   -1.664732
  C      1.795505   -1.863518    0.000225
  C      0.520829   -3.371564   -1.971164
  C      2.258548   -3.132502   -0.317066
  C      1.619558   -3.888159   -1.295175
  H     -0.815370   -1.708040   -2.186059
  H      2.284715   -1.251380    0.752522
  H      0.025757   -3.957569   -2.736809
  H      3.120089   -3.533026    0.204504
  H      1.981502   -4.882274   -1.533153
  C     -0.075337    0.462840    1.662324
  C     -0.916495   -0.284239    2.415097
  H     -0.901350   -0.005961    3.468373
  C      0.687259    1.565676    2.196754
  H      0.232390    2.204774    2.941963
  C      2.068995    1.607301    1.982774
  O      2.737699    0.776841    1.347347
  O      2.685889    2.697780    2.535705
  C      4.084638    2.771451    2.332676
  H      4.601504    1.901365    2.748031
  H      4.332937    2.846347    1.269831
  H      4.414484    3.673547    2.850073
  C     -1.863447   -1.375220    2.110600
  C     -3.206070   -1.240036    2.488192
  C     -1.462456   -2.574875    1.515461
  C     -4.128626   -2.239554    2.214729
  H     -3.527671   -0.324473    2.973668
  C     -2.387518   -3.574519    1.237076
  H     -0.419021   -2.729309    1.274100
  C     -3.725055   -3.406895    1.572676
  H     -5.166351   -2.107252    2.501089
  H     -2.054623   -4.491885    0.764038
  H     -4.445770   -4.187007    1.354327
```

cis-ts3-rot

```
charge, 2S+1    = 0, 1
--- OPT at ωB97X-D/6-311G(d,p) in SMD ---
E(ele)          = -1611.78326735 a.u.
Correction to G = 0.401225 a.u.(RRHO)
Correction to G = 0.406660 a.u.(quasi-RRHO)
imaginary frequency = 83i
--- SP at ωB97X-D/def2-TZVP in SMD ---
E(ele)          = -1611.94560554 a.u.
 P     -0.401819   -0.397511   -0.134698
  C     -1.765972   -1.592456   -0.136385
  C     -3.105815   -1.223432   -0.229583
  C     -1.415018   -2.946141   -0.153630
  C     -4.087459   -2.202339   -0.311744
  C     -2.400837   -3.917751   -0.231769
  C     -3.738839   -3.546283   -0.306717
  H     -3.388502   -0.179644   -0.271124
  H     -0.371295   -3.240616   -0.120758
  H     -5.127338   -1.908774   -0.393970
  H     -2.122656   -4.965112   -0.243055
  H     -4.508897   -4.306339   -0.374546
  C     -1.019894    1.300768    0.000947
  C     -1.975305    1.620555    0.970845
  C     -0.453360    2.300823   -0.787488
  C     -2.403013    2.931898    1.108283
  C     -0.883046    3.614539   -0.632032
  C     -1.862693    3.928069    0.299962
  H     -2.377238    0.854592    1.624902
  H      0.358111    2.063773   -1.471999
  H     -3.149416    3.178645    1.854396
  H     -0.436877    4.393696   -1.239017
  H     -2.195605    4.953856    0.412742
  C      0.567722   -0.635558    1.375697
  C      1.639074    0.239671    1.583911
  C      0.262520   -1.604908    2.328739
  C      2.410071    0.120347    2.729880
  C      1.040035   -1.714463    3.475867
  C      2.115623   -0.858949    3.672491
  H      1.862974    1.017443    0.862087
  H     -0.578806   -2.272631    2.190896
  H      3.244033    0.794773    2.883422
  H      0.800341   -2.469894    4.215147
  H      2.722807   -0.949435    4.566268
  C      0.704578   -0.672448   -1.618121
  C      0.099833   -0.865874   -2.798333
  H      0.772581   -1.089732   -3.624583
  C      2.142638   -0.705904   -1.391354
```

H	2.604698	-1.620662	-1.041614
C	2.870645	0.479054	-1.389964
O	2.456709	1.635016	-1.589968
O	4.208088	0.288928	-1.097507
C	5.000430	1.457240	-1.093032
H	4.674631	2.172187	-0.330200
H	6.017191	1.128811	-0.869139
H	4.987921	1.965088	-2.062328
C	-1.345542	-0.835812	-3.135665
C	-1.998342	-2.025214	-3.471783
C	-2.063610	0.358643	-3.166838
C	-3.348505	-2.023520	-3.786564
H	-1.443956	-2.957467	-3.461981
C	-3.416674	0.360842	-3.489949
H	-1.560324	1.291282	-2.944720
C	-4.063859	-0.829323	-3.791543
H	-3.846118	-2.956514	-4.026579
H	-3.961372	1.298377	-3.507781
H	-5.119523	-0.828650	-4.039437

trans-ts4-Za

charge, 2S+1 = 0, 1
--- OPT at ωB97X-D/6-311G(d,p) in SMD ---
E(ele) = -2472.61876033 a.u.
Correction to G = 0.645802 a.u.(RRHO)
Correction to G = 0.655601 a.u.(quasi-RRHO)
imaginary frequency = 357i
--- SP at ωB97X-D/def2-TZVP in SMD ---
E(ele) = -2472.87986167 a.u.

P	-2.038578	0.983804	0.335053
C	-1.943296	2.176553	1.716853
C	-1.789267	3.529812	1.391614
C	-2.016194	1.799801	3.056429
C	-1.687315	4.481974	2.392831
C	-1.918831	2.760917	4.056295
C	-1.749342	4.098240	3.728312
H	-1.743764	3.840855	0.354343
H	-2.155273	0.765407	3.337692
H	-1.561285	5.525306	2.128698
H	-1.977492	2.456002	5.094457
H	-1.670970	4.843929	4.511278
C	-2.778030	-0.542357	0.970548
C	-2.085994	-1.249526	1.956824
C	-3.990044	-1.027299	0.481179
C	-2.617140	-2.431238	2.457709
C	-4.514904	-2.205345	0.993103
C	-3.832204	-2.906194	1.981419
H	-1.112200	-0.917335	2.308054
H	-4.501828	-0.512304	-0.320558
H	-2.066012	-2.984227	3.208930
H	-5.453654	-2.584139	0.606015
H	-4.241705	-3.831866	2.369684
C	-3.134311	1.851681	-0.819992
C	-4.427698	2.174919	-0.409383
C	-2.659067	2.297647	-2.050187
C	-5.252098	2.915603	-1.244806
C	-3.483925	3.050707	-2.874369
C	-4.780638	3.353385	-2.476940
H	-4.791552	1.859373	0.562638
H	-1.649393	2.061145	-2.364360
H	-6.259559	3.158389	-0.927769
H	-3.111683	3.398019	-3.831026
H	-5.423401	3.937629	-3.125612
C	-0.378203	0.760680	-0.381705
C	0.580861	1.561749	0.168971
H	0.318170	2.097258	1.072345
C	-0.159404	-0.259945	-1.372797
H	0.809035	-0.261923	-1.847577
C	-1.216708	-0.760272	-2.211226
O	-2.416296	-0.693349	-1.981471
O	-0.738114	-1.379568	-3.305773
C	-1.703226	-2.003556	-4.146075
H	-2.342629	-1.256792	-4.624141
H	-1.132290	-2.543780	-4.899514
H	-2.323666	-2.699073	-3.579707
C	1.935363	1.889192	-0.277304
C	2.800325	2.451813	0.674913
C	2.402906	1.775411	-1.593519
C	4.081402	2.857484	0.339784
H	2.452769	2.566560	1.697233
C	3.680808	2.195926	-1.931137
H	1.759021	1.385974	-2.371106
C	4.529358	2.730449	-0.969892
H	4.732576	3.267579	1.103227
H	4.019228	2.094913	-2.956221
H	5.532939	3.040285	-1.238058
C	1.948027	-0.979269	1.605952
O	1.136948	-0.901749	2.521720
C	1.786807	-1.533558	0.283402
C	0.527446	-1.979673	-0.112910
H	-0.212506	-1.810168	0.656976
N	3.319118	-0.567362	1.712848
C	3.967390	-0.800700	0.475676
C	3.036401	-1.371319	-0.416350
C	5.261872	-0.507145	0.083495
C	5.621227	-0.756479	-1.241207
C	4.698812	-1.257316	-2.151095
C	3.401595	-1.557518	-1.748087
H	5.963095	-0.081115	0.782623
H	6.632613	-0.533145	-1.562226
H	4.987323	-1.411822	-3.185226
H	2.680763	-1.923649	-2.469241
C	3.922080	0.038023	2.814655
O	5.105222	0.304580	2.807295
C	3.062524	0.359059	4.008492
H	2.660269	-0.551274	4.454052
H	3.695370	0.883653	4.723331
H	2.204241	0.971011	3.730044
C	0.171850	-3.154323	-0.934149
C	-1.176857	-3.534535	-0.975245
C	-1.583507	-4.660114	-1.674072
C	-0.647559	-5.440222	-2.344849
C	0.697054	-5.094608	-2.284122
C	1.104628	-3.969047	-1.579951
H	-1.913684	-2.930175	-0.457593
H	-2.633985	-4.929867	-1.691838
H	-0.961227	-6.321081	-2.894163
H	1.439424	-5.712696	-2.777208
H	2.160121	-3.745020	-1.508292

trans-ts4-Zg

charge, 2S+1 = 0, 1
--- OPT at ωB97X-D/6-311G(d,p) in SMD ---
E(ele) = -2472.61415909 a.u.
Correction to G = 0.645297 a.u.(RRHO)
Correction to G = 0.655375 a.u.(quasi-RRHO)
imaginary frequency = 406i
--- SP at ωB97X-D/def2-TZVP in SMD ---
E(ele) = -2472.87524496 a.u.

P	-2.200529	0.574309	0.059968
C	-2.248022	1.148794	1.787038
C	-2.350811	0.229822	2.832272
C	-2.144332	2.513780	2.072828
C	-2.350252	0.673731	4.148597
C	-2.143495	2.949243	3.390156
C	-2.248361	2.030390	4.428500
H	-2.434750	-0.830807	2.631191
H	-2.060652	3.237018	1.270015
H	-2.428387	-0.046376	4.954259
H	-2.059900	4.008305	3.603463
H	-2.249068	2.372793	5.456985
C	-3.127985	-0.980034	0.013451
C	-2.495205	-2.157154	0.409006
C	-4.457807	-0.999451	-0.403092
C	-3.203113	-3.352477	0.392149
C	-5.155450	-2.198586	-0.412935
C	-4.530760	-3.375399	-0.015849
H	-1.451582	-2.169462	0.712279
H	-4.945304	-0.096710	-0.749081
H	-2.704177	-4.265826	0.695409
H	-6.186967	-2.213952	-0.745019
H	-5.078521	-4.310943	-0.031951
C	-3.024890	1.849576	-0.921191
C	-2.464657	2.263661	-2.127766
C	-4.216234	2.421563	-0.465869
C	-3.098626	3.244794	-2.877963
C	-4.846843	3.395462	-1.225812
C	-4.287451	3.806930	-2.430891
H	-1.540836	1.821724	-2.479076
H	-4.646463	2.116850	0.481931
H	-2.659525	3.568972	-3.813871
H	-5.771234	3.837518	-0.873175
H	-4.778988	4.572536	-3.020390
C	-0.460382	0.463937	-0.469457
C	0.436171	1.300893	0.253334
H	0.013383	1.824310	1.099180
C	-0.063724	-0.421185	-1.438909
H	0.993105	-0.538604	-1.626006
C	-0.940439	-1.186551	-2.307702
O	-2.090222	-0.908187	-2.588219
O	-0.309171	-2.258199	-2.812931
C	-1.079542	-3.101998	-3.665520
H	-1.423428	-2.555353	-4.546175
H	-0.410358	-3.907614	-3.961304
H	-1.944877	-3.502759	-3.132575
C	1.503217	2.096561	-0.394655
C	2.182991	3.048374	0.383878
C	1.860226	2.007267	-1.746926
C	3.204583	3.823087	-0.144466
H	1.907585	3.179329	1.423870
C	2.870154	2.795358	-2.279849
H	1.355247	1.311187	-2.401681
C	3.565039	3.694992	-1.481112
H	3.717880	4.533818	0.494430
H	3.124572	2.690803	-3.329074
H	4.366301	4.296051	-1.896302
C	1.342611	-2.385701	0.423017
O	0.398707	-2.841950	1.060479
C	2.034065	-1.134227	0.610553
C	1.472857	-0.208152	1.494351
H	0.544703	-0.588684	1.915952
N	2.020320	-3.093681	-0.619607
C	3.125180	-2.322319	-1.048978
C	3.149453	-1.115562	-0.317269
C	4.053728	-2.593404	-2.043528
C	5.019385	-1.628779	-2.320896
C	5.039315	-0.418965	-1.634596
C	4.106060	-0.153879	-0.640580
H	4.016458	-3.523844	-2.587015
H	5.754817	-1.826160	-3.092966
H	5.779697	0.333686	-1.882522
H	4.104617	0.810997	-0.149883
C	1.590628	-4.271299	-1.232268
O	2.249797	-4.794170	-2.105144
C	0.278287	-4.862924	-0.790070
H	0.317741	-5.164629	0.256547
H	-0.525837	-4.132896	-0.881358
H	0.087751	-5.725119	-1.428309
C	2.162951	0.690432	2.443216
C	3.550206	0.811458	2.524452
C	4.136205	1.672766	3.445350
C	3.349791	2.417863	4.313631
C	1.965249	2.273450	4.274619
C	1.384976	1.415377	3.355695
H	4.182355	0.213390	1.882671
H	5.216820	1.754182	3.485459
H	3.810141	3.091336	5.027970
H	1.338195	2.828013	4.964567
H	0.305799	1.307562	3.330504

trans-ts4-Ea

charge, 2S+1 = 0, 1
--- OPT at ωB97X-D/6-311G(d,p) in SMD ---
E(ele) = -2472.61113259 a.u.
Correction to G = 0.646848 a.u.(RRHO)
Correction to G = 0.656166 a.u.(quasi-RRHO)
imaginary frequency = 319i
--- SP at ωB97X-D/def2-TZVP in SMD ---
E(ele) = -2472.87227949 a.u.

P	-1.915536	-1.428669	-0.097741
C	-1.630012	-2.372485	-1.619376
C	-0.863106	-3.535858	-1.621462
C	-2.189428	-1.905209	-2.813002
C	-0.654071	-4.223062	-2.808798
C	-1.968549	-2.592915	-3.997039
C	-1.201505	-3.752005	-3.995196
H	-0.420564	-3.907996	-0.707903
H	-2.792214	-1.004299	-2.822079
H	-0.055025	-5.125915	-2.804870
H	-2.397973	-2.221520	-4.919909
H	-1.031128	-4.289559	-4.921187
C	-3.646754	-0.898567	-0.180371
C	-4.633939	-1.820611	-0.543048

C	-3.993445	0.416927	0.122933
C	-5.962804	-1.427929	-0.582514
C	-5.327731	0.803579	0.070437
C	-6.309275	-0.115196	-0.275077
H	-4.364829	-2.839829	-0.799225
H	-3.229645	1.144313	0.372578
H	-6.727599	-2.143584	-0.860619
H	-5.593067	1.829708	0.295435
H	-7.348402	0.191680	-0.313652
C	-1.714917	-2.512859	1.339221
C	-0.419314	-2.906527	1.685096
C	-2.796346	-2.899690	2.129206
C	-0.209626	-3.693052	2.807126
C	-2.578267	-3.692163	3.249913
C	-1.290457	-4.087562	3.588992
H	0.412603	-2.581630	1.070231
H	-3.801973	-2.578380	1.886473
H	0.799476	-3.982394	3.078358
H	-3.419194	-3.991077	3.864638
H	-1.125953	-4.696506	4.470588
C	-0.894695	0.066433	0.042061
C	-0.577745	0.382280	1.321716
H	-0.856487	-0.352485	2.071420
C	-0.645045	0.682342	-1.247463
H	-0.648494	-0.024234	-2.066824
C	-1.266201	1.919941	-1.662417
O	-1.804922	2.776505	-0.979352
O	-1.196060	2.046825	-3.010083
C	-1.622710	3.293933	-3.541068
H	-2.691801	3.449392	-3.374051
H	-1.066970	4.119779	-3.092489
H	-1.415821	3.246891	-4.609542
C	0.081781	1.536247	1.947489
C	-0.047131	2.856188	1.515686
C	0.827686	1.281569	3.107908
C	0.563786	3.888399	2.219986
H	-0.663152	3.071378	0.652274
C	1.443949	2.310173	3.799999
H	0.933326	0.259400	3.458505
C	1.311143	3.622867	3.358938
H	0.444399	4.910179	1.874650
H	2.034160	2.087005	4.681225
H	1.790014	4.430753	3.901122
C	2.163718	-0.980783	-0.077646
O	1.528170	-1.859205	-0.655532
C	2.243337	0.422268	-0.379364
C	1.586061	0.854673	-1.532454
H	1.419557	0.043413	-2.230943
N	2.935733	-1.212563	1.101304
C	3.475101	0.020843	1.539090
C	3.080017	1.033348	0.639167
C	4.315575	0.267161	2.612350
C	4.789436	1.564494	2.787688
C	4.427382	2.575557	1.905769
C	3.576988	2.319368	0.836503
H	4.586176	-0.526838	3.291077
H	5.445788	1.780667	3.623321
H	4.805156	3.581417	2.053350
H	3.320201	3.119823	0.158333
C	3.224081	-2.453288	1.669940
O	3.649533	-2.533866	2.802535
C	3.050768	-3.686748	0.817148
H	2.010185	-4.011613	0.797317
H	3.352324	-3.518671	-0.215548
H	3.655204	-4.469685	1.274952
C	1.733690	2.167046	-2.196864
C	2.013677	2.186614	-3.565611
C	2.155376	3.386564	-4.249083
C	2.000714	4.593584	-3.577493
C	1.697299	4.587393	-2.220316
C	1.561077	3.385734	-1.539597
H	2.118873	1.247179	-4.098067
H	2.381075	3.378516	-5.309845
H	2.105649	5.532574	-4.109737
H	1.554561	5.522333	-1.689374
H	1.291233	3.386669	-0.492474

trans-ts4-Eg

charge, 2S+1 = 0, 1
--- OPT at ωB97X-D/6-311G(d,p) in SMD ---
E(ele) = -2472.60875516 a.u.
Correction to G = 0.647435 a.u.(RRHO)
Correction to G = 0.656678 a.u.(quasi-RRHO)
imaginary frequency = 362i
--- SP at ωB97X-D/def2-TZVP in SMD ---
E(ele) = -2472.86829551 a.u.

P	2.230313	0.153819	0.118843
C	2.560798	-0.664306	1.706201
C	3.540112	-1.655383	1.786666
C	1.808974	-0.330041	2.837678
C	3.771469	-2.301578	2.994436
C	2.049991	-0.982570	4.037953
C	3.029612	-1.966325	4.118986
H	4.116505	-1.937389	0.914614
H	1.030689	0.421937	2.758994
H	4.530171	-3.073337	3.050180
H	1.463942	-0.722708	4.911940
H	3.210606	-2.475742	5.058792
C	2.719102	1.897907	0.213095
C	2.869786	2.620308	-0.974820
C	3.014755	2.502133	1.433884
C	3.290904	3.941537	-0.934165
C	3.450961	3.819811	1.464614
C	3.584129	4.541159	0.285219
H	2.679919	2.150724	-1.933644
H	2.907773	1.954835	2.360868
H	3.397003	4.498432	-1.857451
H	3.680239	4.284022	2.416516
H	3.918525	5.571779	0.314930
C	3.389802	-0.565679	-1.082862
C	2.978818	-1.587935	-1.936363
C	4.718661	-0.131069	-1.104747
C	3.889983	-2.166994	-2.810740
C	5.622576	-0.715561	-1.979255
C	5.208599	-1.732563	-2.833583
H	1.955031	-1.938609	-1.924869
H	5.049215	0.661680	-0.443109
H	3.561491	-2.958875	-3.473471
H	6.650842	-0.373788	-1.995594
H	5.916692	-2.184659	-3.518799
C	0.549974	-0.223810	-0.479996
C	0.032507	-1.450998	0.045543
H	0.572833	-1.787324	0.924505
C	-0.004229	0.705654	-1.312569
H	0.520799	1.636619	-1.484390
C	-1.301224	0.658781	-1.987363
O	-2.042421	-0.285859	-2.148256
O	-1.583877	1.885702	-2.475045
C	-2.830822	2.010465	-3.156705
H	-2.962785	3.075867	-3.336556
H	-2.801797	1.469845	-4.106894
H	-3.649855	1.627165	-2.548504
C	-0.329957	-2.636872	-0.757030
C	-0.460998	-2.653355	-2.150141
C	-0.466956	-3.858818	-0.079922
C	-0.756480	-3.830765	-2.825669
H	-0.338702	-1.740088	-2.712960
C	-0.768357	-5.031674	-0.751758
H	-0.350544	-3.878596	0.998731
C	-0.924140	-5.024012	-2.133886
H	-0.859358	-3.810634	-3.905588
H	-0.884957	-5.954072	-0.193288
H	-1.163255	-5.938338	-2.665701
C	-1.226556	1.706555	1.177563
O	-0.126413	1.707679	1.730958
C	-2.077509	0.587527	0.883371
C	-1.652336	-0.698833	1.262077
H	-0.938773	-0.673236	2.079273
N	-1.898256	2.889664	0.734846
C	-3.182491	2.528712	0.270344
C	-3.314041	1.125914	0.339130
C	-4.211380	3.345867	-0.176171
C	-5.407440	2.746423	-0.559406
C	-5.565720	1.367057	-0.484625
C	-4.528863	0.556068	-0.039807
H	-4.080481	4.414884	-0.217879
H	-6.222284	3.369529	-0.911195
H	-6.506899	0.913431	-0.775856
H	-4.678546	-0.510994	0.016915
C	-1.381549	4.184687	0.697560
O	-2.066883	5.118884	0.340546
C	0.057576	4.378783	1.082669
H	0.703601	3.756821	0.464021
H	0.290701	5.432403	0.935511
H	0.235677	4.085344	2.116370
C	-2.527429	-1.890004	1.285877
C	-2.638850	-2.626078	2.467158
C	-3.432662	-3.765433	2.524086
C	-4.116207	-4.193662	1.392466
C	-3.991876	-3.481438	0.204159
C	-3.198170	-2.345596	0.147827
H	-2.102129	-2.297088	3.351937
H	-3.515729	-4.318938	3.453229
H	-4.732250	-5.085373	1.432648
H	-4.497605	-3.824283	-0.691666
H	-3.055721	-1.816637	-0.787481

trans-4-Za

charge, 2S+1 = 0, 1
--- OPT at ωB97X-D/6-311G(d,p) in SMD ---
E(ele) = -2472.65287224 a.u.
Correction to G = 0.648431 a.u.(RRHO)
Correction to G = 0.658523 a.u.(quasi-RRHO)
--- SP at ωB97X-D/def2-TZVP in SMD ---
E(ele) = -2472.91318389 a.u.

P	1.560358	1.009725	0.238055
C	2.237307	-0.060336	-1.045494
C	3.436841	-0.742674	-0.835124
C	1.615814	-0.096827	-2.294520
C	4.003689	-1.465624	-1.876320
C	2.194434	-0.815748	-3.327996
C	3.385539	-1.501311	-3.119979
H	3.925971	-0.720062	0.131089
H	0.661831	0.390389	-2.453126
H	4.925654	-2.010019	-1.709238
H	1.690790	-0.871565	-4.284745
H	3.820512	-2.081745	-3.924940
C	2.074985	0.445390	1.881412
C	2.116041	-0.919785	2.171392
C	2.403966	1.382696	2.865685
C	2.466494	-1.335590	3.449360
C	2.756587	0.952708	4.136012
C	2.783367	-0.405936	4.430482
H	1.859851	-1.674646	1.428719
H	2.389866	2.443509	2.652473
H	2.486156	-2.396429	3.669650
H	3.011253	1.682750	4.895357
H	3.057164	-0.738084	5.425745
C	2.271056	2.664512	-0.006957
C	1.663578	3.775010	0.589217
C	3.444490	2.820619	-0.742824
C	2.236005	5.029924	0.447783
C	4.007657	4.083300	-0.882441
C	3.406408	5.184731	-0.288271
H	0.745885	3.657298	1.156280
H	3.914637	1.967534	-1.217487
H	1.762517	5.888814	0.908404
H	4.915271	4.203148	-1.462065
H	3.846738	6.168695	-0.402613
C	-0.242184	1.206990	0.139439
C	-0.659554	2.093815	-0.777662
H	0.080034	2.532239	-1.443970
C	-1.151334	0.473606	1.108534
H	-2.160052	0.767626	0.811736
C	-0.993470	1.087185	2.498207
O	-0.918010	2.280283	2.690921
O	-0.967422	0.188937	3.468646
C	-0.870599	0.689926	4.804450
H	-0.763856	-0.188589	5.436683
H	-0.004103	1.343536	4.906663
H	-1.777155	1.237846	5.068539
C	-2.026565	2.590473	-1.018769
C	-2.870187	2.989133	0.021078
C	-2.471078	2.712226	-2.338842
C	-4.150963	3.452707	-0.256091
H	-2.509953	2.965997	1.043110
C	-3.752108	3.165821	-2.610834
H	-1.817458	2.415604	-3.151841
C	-4.598753	3.530767	-1.568382
H	-4.796214	3.760508	0.558908
H	-4.091800	3.234388	-3.637973
H	-5.600332	3.886908	-1.781349
C	0.260318	-2.647983	-0.393744

O	1.011413	-3.163123	0.454552
C	-0.709691	-1.643925	-0.270670
C	-1.077138	-1.076049	1.070365
H	-0.277656	-1.381672	1.743629
N	0.282451	-3.043514	-1.793048
C	-0.712159	-2.319789	-2.477133
C	-1.349022	-1.473291	-1.534326
C	-1.084825	-2.340774	-3.810757
C	-2.126145	-1.504883	-4.223159
C	-2.775612	-0.679927	-3.310211
C	-2.396902	-0.659756	-1.971361
H	-0.579985	-2.993132	-4.506218
H	-2.434015	-1.514968	-5.263048
H	-3.591631	-0.044780	-3.640389
H	-2.918594	-0.013495	-1.276570
C	1.153966	-3.938011	-2.393922
O	1.121729	-4.136718	-3.594422
C	2.147935	-4.662036	-1.524638
H	2.743131	-5.296422	-2.181209
H	2.778226	-3.957948	-0.982376
H	1.639623	-5.260778	-0.768184
C	-2.339718	-1.731027	1.625723
C	-2.192833	-2.913564	2.354588
C	-3.296121	-3.598353	2.841388
C	-4.579287	-3.112540	2.609092
C	-4.739992	-1.941891	1.881689
C	-3.629851	-1.258179	1.394519
H	-1.193533	-3.303469	2.519492
H	-3.155205	-4.515642	3.403217
H	-5.444519	-3.645304	2.987991
H	-5.734528	-1.556274	1.684218
H	-3.795470	-0.352661	0.820931

trans-ts5-Za (syn)

charge, 2S+1 = 0, 1
--- OPT at ωB97X-D/6-311G(d,p) in SMD ---
E(ele) = -2472.62919632 a.u.
Correction to G = 0.645661 a.u.(RRHO)
Correction to G = 0.656352 a.u.(quasi-RRHO)
imaginary frequency = 300i
--- SP at ωB97X-D/def2-TZVP in SMD ---
E(ele) = -2472.88966241 a.u.

P	2.052457	-0.791322	0.207818
C	1.849214	-1.645034	1.803497
C	1.444612	-2.983570	1.794416
C	2.084137	-1.002434	3.016636
C	1.261551	-3.663174	2.990112
C	1.920087	-1.695082	4.209564
C	1.503565	-3.020169	4.198836
H	1.274407	-3.496807	0.854279
H	2.391097	0.034319	3.042103
H	0.936931	-4.696963	2.975620
H	2.108977	-1.190303	5.149808
H	1.367789	-3.553292	5.133015
C	3.110835	0.667503	0.431224
C	2.592011	1.752757	1.143217
C	4.393605	0.740094	-0.113351
C	3.360844	2.894931	1.313546
C	5.154300	1.888612	0.061833
C	4.639221	2.964954	0.773811
H	1.587403	1.698287	1.554174
H	4.796992	-0.085567	-0.686437
H	2.951597	3.736869	1.859322
H	6.148556	1.943703	-0.366547
H	5.233169	3.862912	0.901748
C	3.026464	-1.998527	-0.759142
C	4.162492	-2.590887	-0.200911
C	2.613948	-2.359378	-2.037465
C	4.888645	-3.521231	-0.929500
C	3.342883	-3.294493	-2.763778
C	4.479440	-3.871900	-2.212903
H	4.476058	-2.331183	0.805045
H	1.724877	-1.904307	-2.455867
H	5.768796	-3.979480	-0.493405
H	3.016902	-3.575376	-3.758721
H	5.044635	-4.603750	-2.779154
C	0.526097	-0.444239	-0.552997
C	-0.629550	-1.058656	-0.046139
H	-0.537205	-1.531576	0.929957
C	0.248290	0.761186	-1.415261
H	-0.342418	0.444367	-2.278203
C	1.441939	1.471121	-2.023083
O	1.691076	2.641995	-1.934763
O	2.188620	0.614296	-2.740191
C	3.333026	1.167164	-3.391374
H	3.947692	1.725509	-2.684247
H	3.884785	0.317182	-3.789841
H	3.025801	1.830452	-4.202926
C	-1.734501	-1.667222	-0.813694
C	-2.796261	-2.235275	-0.100331
C	-1.761997	-1.746957	-2.208499
C	-3.874441	-2.809398	-0.754889
H	-2.778080	-2.210127	0.983991
C	-2.835109	-2.333250	-2.865894
H	-0.925877	-1.376677	-2.789452
C	-3.902368	-2.853774	-2.143641
H	-4.696024	-3.218346	-0.178061
H	-2.835022	-2.386924	-3.949203
H	-4.745105	-3.300063	-2.659423
C	-1.322140	0.841714	1.710317
O	-0.268509	1.055089	2.302234
C	-1.603769	0.961811	0.309411
C	-0.652419	1.733802	-0.567488
H	0.038467	2.253533	0.101376
N	-2.531748	0.401379	2.352346
C	-3.579471	0.439551	1.400889
C	-3.034891	0.810415	0.157127
C	-4.926525	0.145526	1.550264
C	-5.741160	0.228478	0.422725
C	-5.219018	0.589235	-0.815219
C	-3.867467	0.878158	-0.956166
H	-5.323653	-0.139006	2.511651
H	-6.798297	0.006854	0.519938
H	-5.870208	0.642815	-1.680737
H	-3.466606	1.150991	-1.923598
C	-2.671672	-0.019662	3.672708
O	-3.758260	-0.329746	4.113837
C	-1.430905	-0.105534	4.519975
H	-1.729393	-0.516674	5.483557
H	-0.678200	-0.740795	4.051279
H	-0.974170	0.875837	4.646675
C	-1.358041	2.815652	-1.373172
C	-1.498490	2.796293	-2.757963
C	-2.198386	3.799968	-3.421554
C	-2.769402	4.844896	-2.710407
C	-2.628740	4.881471	-1.326888
C	-1.929898	3.879549	-0.672086
H	-1.069231	1.995162	-3.348494
H	-2.295121	3.759951	-4.501269
H	-3.317542	5.625026	-3.226831
H	-3.068697	5.691862	-0.755954
H	-1.834839	3.908143	0.408944

trans-ts5-Za (anti)

charge, 2S+1 = 0, 1
--- OPT at ωB97X-D/6-311G(d,p) in SMD ---
E(ele) = -2472.62272420 a.u.
Correction to G = 0.647349 a.u.(RRHO)
Correction to G = 0.657338 a.u.(quasi-RRHO)
imaginary frequency = 259i
--- SP at ωB97X-D/def2-TZVP in SMD ---
E(ele) = -2472.88340457 a.u.

P	-2.091175	0.605133	0.248662
C	-2.026081	0.283034	2.043304
C	-1.779509	1.317369	2.947510
C	-2.068383	-1.039335	2.498703
C	-1.611470	1.035654	4.297329
C	-1.896813	-1.311608	3.848497
C	-1.673847	-0.275584	4.749728
H	-1.705825	2.343527	2.609206
H	-2.207372	-1.853208	1.795131
H	-1.417969	1.844836	4.991554
H	-1.929159	-2.338413	4.193645
H	-1.536068	-0.493197	5.802729
C	-3.518136	-0.265264	-0.464134
C	-4.537669	-0.799568	0.319237
C	-3.628224	-0.274271	-1.855946
C	-5.650620	-1.364910	-0.289834
C	-4.743278	-0.839514	-2.457015
C	-5.753599	-1.388298	-1.674904
H	-4.468487	-0.787688	1.399996
H	-2.841156	0.153719	-2.466730
H	-6.438031	-1.788951	0.322215
H	-4.821939	-0.850753	-3.537881
H	-6.623662	-1.831254	-2.146116
C	-2.559973	2.355710	0.039011
C	-3.610911	2.850136	0.819189
C	-2.018489	3.155188	-0.966906
C	-4.079072	4.139816	0.623508
C	-2.491012	4.448952	-1.155705
C	-3.513564	4.944830	-0.359870
H	-4.070631	2.229002	1.580022
H	-1.237674	2.778527	-1.613435
H	-4.889474	4.514916	1.237424
H	-2.055857	5.064597	-1.934057
H	-3.878835	5.954299	-0.510395
C	-0.638706	0.250660	-0.678168
C	0.387848	1.199254	-0.598899
H	0.291624	1.974428	0.161651
C	-0.156413	-1.155965	-0.949939
H	0.472666	-1.158902	-1.839970
C	-1.214390	-2.219286	-1.133055
O	-1.827243	-2.743481	-0.234387
O	-1.389220	-2.537360	-2.414417
C	-2.348379	-3.567364	-2.673695
H	-2.418850	-3.636550	-3.757403
H	-2.003254	-4.515143	-2.256299
H	-3.316704	-3.309559	-2.242286
C	1.288202	1.635294	-1.675589
C	2.219614	2.644105	-1.391795
C	1.190710	1.169766	-2.990073
C	3.043434	3.152400	-2.382955
H	2.300954	3.019200	-0.376397
C	2.008708	1.687453	-3.984450
H	0.451168	0.419071	-3.242534
C	2.940017	2.675001	-3.686284
H	3.768029	3.920662	-2.137919
H	1.918208	1.315257	-4.998839
H	3.580696	3.073613	-4.464902
C	2.938603	-0.409447	-0.523826
O	3.048713	-0.913674	-1.634402
C	1.844529	-0.477525	0.394046
C	0.770440	-1.505563	0.278375
H	0.141909	-1.417138	1.166840
N	3.928243	0.452064	0.072797
C	3.500205	0.808291	1.372789
C	2.247357	0.200745	1.599880
C	4.111423	1.586343	2.341892
C	3.461688	1.739290	3.569978
C	2.240832	1.120823	3.817153
C	1.627500	0.346379	2.836166
H	5.064419	2.053887	2.149526
H	3.929213	2.342197	4.340580
H	1.761130	1.238653	4.783432
H	0.676406	-0.136057	3.031494
C	5.079828	0.942365	-0.539105
O	5.786885	1.748921	0.029476
C	5.417183	0.438914	-1.915184
H	5.573260	-0.640844	-1.904123
H	4.601348	0.627292	-2.612434
H	6.325462	0.951711	-2.229962
C	1.275406	-2.938282	0.259668
C	1.253280	-3.672573	1.446135
C	1.699119	-4.986721	1.484300
C	2.168167	-5.596275	0.326270
C	2.192158	-4.875848	-0.861363
C	1.752435	-3.557289	-0.896458
H	0.881411	-3.204007	2.352172
H	1.675994	-5.537023	2.418859
H	2.514304	-6.623811	0.350491
H	2.563243	-5.338803	-1.769584
H	1.813821	-2.995342	-1.819140

trans-5-Za

charge, 2S+1 = 0, 1
--- OPT at ωB97X-D/6-311G(d,p) in SMD ---
E(ele) = -2472.66247038 a.u.
Correction to G = 0.646648 a.u.(RRHO)
Correction to G = 0.658621 a.u.(quasi-RRHO)
--- SP at ωB97X-D/def2-TZVP in SMD ---
E(ele) = -2472.92415067 a.u.

P	2.291545	-0.394039	0.206126
C	2.959113	-0.559154	1.917628

C	2.186089	-0.091990	2.975996
C	4.189917	-1.169447	2.178465
C	2.638111	-0.227190	4.284800
C	4.643477	-1.294942	3.483267
C	3.864533	-0.825903	4.538246
H	1.243863	0.400553	2.768166
H	4.791796	-1.552564	1.360552
H	2.029971	0.138852	5.104201
H	5.600417	-1.765334	3.680113
H	4.216734	-0.931103	5.558573
C	3.324580	0.856004	-0.622357
C	4.405641	1.467477	0.011004
C	2.984204	1.238558	-1.922642
C	5.141887	2.441118	-0.653603
C	3.724033	2.207394	-2.583776
C	4.804926	2.809469	-1.949786
H	4.665256	1.202010	1.028486
H	2.123012	0.787460	-2.402641
H	5.976957	2.916427	-0.151687
H	3.449395	2.500596	-3.590454
H	5.380430	3.571012	-2.464261
C	2.695732	-2.045743	-0.474283
C	2.438261	-3.182564	0.293876
C	3.165866	-2.193717	-1.777556
C	2.627312	-4.447721	-0.242847
C	3.368645	-3.461726	-2.307835
C	3.092762	-4.589189	-1.544486
H	2.073944	-3.084522	1.309950
H	3.380704	-1.322957	-2.385886
H	2.401707	-5.322833	0.355197
H	3.741726	-3.566943	-3.320385
H	3.240573	-5.578253	-1.963500
C	0.672150	0.037291	-0.032825
C	-0.480393	-0.842103	0.450352
H	-0.477165	-0.951011	1.544732
C	0.199671	1.493168	-0.130283
H	0.104352	1.821661	-1.169835
C	1.020594	2.565644	0.562501
O	1.282405	2.604841	1.739708
O	1.401906	3.514293	-0.297670
C	2.186214	4.575328	0.246875
H	3.071070	4.180220	0.748171
H	2.476930	5.192330	-0.601316
H	1.598416	5.161730	0.956320
C	-0.627205	-2.246104	-0.107786
C	-1.121860	-3.251782	0.721758
C	-0.312113	-2.571964	-1.426068
C	-1.300105	-4.548046	0.250401
H	-1.363900	-3.015884	1.754011
C	-0.489375	-3.861898	-1.902372
H	0.102855	-1.808322	-2.070634
C	-0.983751	-4.857250	-1.065533
H	-1.683060	-5.315548	0.914485
H	-0.228930	-4.095299	-2.928950
H	-1.116405	-5.867530	-1.437074
C	-2.895694	-0.317697	1.068933
O	-2.847245	-0.396528	2.270293
C	-1.727332	0.049874	0.164473
C	-1.186461	1.453707	0.543135
H	-1.003925	1.405826	1.621197
N	-4.031159	-0.534005	0.270032
C	-3.680020	-0.373923	-1.103957
C	-2.329986	-0.042794	-1.204440
C	-4.481110	-0.502167	-2.229600
C	-3.891728	-0.290077	-3.473138
C	-2.546930	0.035257	-3.589461
C	-1.759735	0.159854	-2.449382
H	-5.523911	-0.758656	-2.141797
H	-4.502447	-0.386621	-4.363791
H	-2.108080	0.188407	-4.568775
H	-0.706154	0.396405	-2.530736
C	-5.324080	-0.849482	0.731236
O	-6.219030	-1.037511	-0.057495
C	-5.544198	-0.932913	2.215017
H	-5.282519	0.005443	2.705570
H	-6.598719	-1.157737	2.366636
H	-4.923459	-1.709612	2.662718
C	-2.129686	2.604850	0.286781
C	-2.260446	3.216481	-0.960660
C	-3.178362	4.240352	-1.156319
C	-3.983855	4.671069	-0.109044
C	-3.860908	4.073614	1.139119
C	-2.939114	3.052560	1.332781
H	-1.648860	2.890242	-1.792536
H	-3.266337	4.700913	-2.134151
H	-4.701383	5.469073	-0.264636
H	-4.479791	4.404759	1.965823
H	-2.842726	2.591263	2.310587

References

(1) Dudding, T.; Kwon, O.; Mercier, E. Theoretical Rationale for Regioselection in Phosphine-Catalyzed Allenoate Additions to Acrylates, Imines, and Aldehydes. *Org. Lett.* **2006**, *8*, 3643–3646.

(2) Mercier, E.; Fonovic, B.; Henry, C.; Kwon, O.; Dudding, T. Phosphine triggered [3+2] allenoate-acrylate annulation: a mechanistic enlightenment. *Tetrahedron Lett.* **2007**, *48*, 3617–3620.

(3) Liang, Y.; Liu, S.; Xia, Y.; Li, Y.; Yu, Z. X. Mechanism, regioselectivity, and the kinetics of phosphine-catalyzed [3+2] cycloaddition reactions of allenoates and electron-deficient alkenes. *Chem. Eur. J.* **2008**, *14*, 4361–4373.

(4) Xia, Y.; Liang, Y.; Chen, Y.; Wang, M.; Jiao, L.; Huang, F.; Liu, S.; Li, Y.; Yu, Z.-X. An Unexpected Role of a Trace Amount of Water in Catalyzing Proton Transfer in Phosphine-Catalyzed (3 + 2) Cycloaddition of Allenoates and Alkenes. *J. Am. Chem. Soc.* **2007**, *129*, 3470–3471.

(5) Huang, G.-T.; Lankau, T.; Yu, C.-H. A Computational Study: Reactivity Difference between Phosphine- and Amine-Catalyzed Cycloadditions of Allenoates and Enones. *J. Org. Chem.* **2014**, *79*, 1700–1711.

(6) Pracht, P.; Bohle, F.; Grimme, S. Automated exploration of the low-energy chemical space with fast quantum chemical methods. *Phys. Chem. Chem. Phys.* **2020**, *22*, 7169–7192.

(7) Pracht, P.; Grimme, S.; Bannwarth, C.; Bohle, F.; Ehlert, S.; Feldmann, G.; Gorges, J.; Müller, M.; Neudecker, T.; Plett, C.; others CREST—A program for the exploration of low-energy molecular chemical space. *J. Chem. Phys.* **2024**, *160*, 114110.

(8) Ehlert, S.; Stahn, M.; Spicher, S.; Grimme, S. Robust and efficient implicit solvation model for fast semiempirical methods. *J. Chem. Theory Comput.* **2021**, *17*, 4250–4261.

(9) Grimme, S.; Bannwarth, C.; Shushkov, P. A robust and accurate tight-binding quantum chemical method for structures, vibrational frequencies, and noncovalent interactions of large molecular systems parametrized for all spd-block elements (Z= 1–86). *J. Chem. Theory Comput.* **2017**, *13*, 1989–2009.

(10) Bannwarth, C.; Ehlert, S.; Grimme, S. GFN2-xTB—An accurate and broadly parametrized self-consistent tight-binding quantum chemical method with multipole electrostatics and density-dependent dispersion contributions. *J. Chem. Theory Comput.* **2019**, *15*, 1652–1671.

(11) Chai, J.-D.; Head-Gordon, M. Long-range corrected hybrid density functionals with damped atom–atom dispersion corrections. *Phys. Chem. Chem. Phys.* **2008**, *10*, 6615–6620.

(12) Hariharan, P. C.; Pople, J. A. The influence of polarization functions on molecular orbital hydrogenation energies. *Theor. Chim. Acta.* **1973**, *28*, 213–222.

(13) Weigend, F.; Ahlrichs, R. Balanced basis sets of split valence, triple zeta valence and quadruple zeta valence quality for H to Rn: Design and assessment of accuracy. *Phys. Chem. Chem. Phys.* **2005**, *7*, 3297–3305.

(14) Marenich, A. V.; Cramer, C. J.; Truhlar, D. G. Universal solvation model based on solute electron density and on a continuum model of the solvent defined by the bulk dielectric constant and atomic surface tensions. *J. Phys. Chem. B* **2009**, *113*, 6378–6396.

(15) Grimme, S. Supramolecular binding thermodynamics by dispersion-corrected density functional theory. *Chem. Eur. J.* **2012**, *18*, 9955–9964.

(16) Becke, A. D.; Edgecombe, K. E. A simple measure of electron localization in atomic and molecular systems. *J. Chem. Phys.* **1990**, *92*, 5397–5403.

(17) Johnson, E. R.; Keinan, S.; Mori-Sánchez, P.; Contreras-García, J.; Cohen, A. J.; Yang, W. Revealing noncovalent interactions. *J. Am. Chem. Soc.* **2010**, *132*, 6498–6506.

(18) Lu, T.; Chen, F. Multiwfn: a multifunctional wavefunction analyzer. *J. Comput. Chem.* **2012**, *33*, 580–592.

(19) Lu, T.; Chen, Q. Shermo: A general code for calculating molecular thermochemistry properties. *Comput. Theor. Chem.* **2021**, *1200*, 113249.

(20) Frisch, M. J.; Trucks, G. W.; Schlegel, H. B.; Scuseria, G. E.; Robb, M. A.; Cheeseman, J. R.; Scalmani, G.; Barone, V.; Mennucci, B.; Petersson, G. A.; Nakatsuji, H.; Caricato, M.; Li, X.; Hratchian, H. P.; Izmaylov, A. F.; Bloino, J.; Zheng, G.; Sonnenberg, J. L.; Hada, M.; Ehara, M.; Toyota, K.; Fukuda, R.; Hasegawa, J.; Ishida, M.; Nakajima, T.; Honda, Y.; Kitao, O.; Nakai, H.; Vreven, T.; Montgomery, J. A., Jr.; Peralta, J. E.; Ogliaro, F.; Bearpark, M.; Heyd, J. J.; Brothers, E.; Kudin, K. N.; Staroverov, V. N.; Kobayashi, R.; Normand, J.; Raghavachari, K.; Rendell, A.; Burant, J. C.; Iyengar, S. S.; Tomasi, J.; Cossi, M.; Rega, N.; Millam, J. M.; Klene, M.; Knox, J. E.; Cross, J. B.; Bakken, V.; Adamo, C.; Jaramillo, J.; Gomperts, R.; Stratmann, R. E.; Yazyev, O.; Austin, A. J.; Cammi, R.; Pomelli, C.; Ochterski, J. W.; Martin, R. L.; Morokuma, K.; Zakrzewski, V. G.; Voth, G. A.; Salvador, P.; Dannenberg, J. J.; Dapprich, S.; Daniels, A. D.; Farkas, O.; Foresman, J. B.; Ortiz, J. V.; Cioslowski, J.; Fox, D. J. Gaussian 09 Revision E.01. Gaussian Inc. Wallingford CT 2009.

(21) Zheng, J.; Xu, X.; Truhlar, D. G. Minimally augmented Karlsruhe basis sets. *Theor. Chem. Acc.* **2011**, *128*, 295–305.

(22) Becke, A. D. Density-functional exchange-energy approximation with correct asymptotic behavior. *Phys. Rev. A* **1988**, *38*, 3098–3010.

(23) Lee, C.; Yang, W.; Parr, R. G. Development of the Colle-Salvetti correlation-energy formula into a functional of the electron density. *Phys. Rev. B* **1988**, *37*, 785–789.

(24) Vosko, S.; Wilk, L.; Nusair, M. Accurate spin-dependent electron liquid correlation energies for local spin density calculations: a critical analysis. *Can. J. Phys.* **1980**, *58*, 1200–1211.

(25) Becke, A. D. Density-functional thermochemistry. III. The role of exact exchange. *J. Chem. Phys.* **1993**, *98*, 5648–5652.

(26) Grimme, S.; Antony, J.; Ehrlich, S.; Krieg, H. A consistent and accurate ab initio parametrization of density functional dispersion correction (DFT-D) for the 94 elements H-Pu. *J. Chem. Phys.* **2010**, *132*.

(27) Zhao, Y.; Truhlar, D. G. The M06 suite of density functionals for main group thermochemistry, thermochemical kinetics, noncovalent interactions, excited states, and transition elements: two new functionals and systematic testing of four M06-class functionals and 12 other function. *Theor. Chem. Acc.* **2007**, *120*, 215–241.

(28) Riplinger, C.; Sandhoefer, B.; Hansen, A.; Neese, F. Natural triple excitations in local coupled cluster calculations with pair natural orbitals. *J. Chem. Phys.* **2013**, *139*, 134101.

(29) Guo, Y.; Riplinger, C.; Becker, U.; Liakos, D. G.; Minenkov, Y.; Cavallo, L.; Neese, F. Communication: An improved linear scaling perturbative triples correction for the domain based local pair-natural orbital based singles and doubles coupled cluster method [DLPNO-CCSD(T)]. *J. Chem. Phys.* **2018**, *148*, 011101.

(30) Neese, F.; Wennmohs, F.; Becker, U.; Riplinger, C. The ORCA quantum chemistry program package. *J. Chem. Phys.* **2020**, *152*, 224108.

(31) Peng, C.; Bernhard Schlegel, H. Combining synchronous transit and quasi-newton methods to find transition states. *Isr. J. Chem.* **1993**, *33*, 449–454.

(32) Wiberg, K. B. Application of the pople-santry-segal CNDO method to the cyclopropylcarbinyl and cyclobutyl cation and to bicyclobutane. *Tetrahedron* **1968**, *24*, 1083–1096.
